# Worldwide trends and future projections of fungal skin disease burden: a comprehensive analysis from the Global Burden of Diseases study 2021

**DOI:** 10.3389/fpubh.2025.1580221

**Published:** 2025-06-04

**Authors:** Deng Li, Siqi Fan, Haochen Zhao, Jiayi Song, Linfen Guo, Wei Li, Xuewen Xu, Qingfeng Li

**Affiliations:** ^1^Department of Plastic and Burns Surgery, West China Hospital, Sichuan University, Chengdu, China; ^2^Department of Urology, West China Hospital, Sichuan University, Chengdu, China; ^3^Department of Plastic and Reconstructive Surgery, Shanghai Ninth People's Hospital Affiliated to Shanghai Jiaotong University School of Medicine, Shanghai, China

**Keywords:** fungal skin diseases, disability-adjusted life-years, Global Burden of Disease, incidence, prevalence, trend, age-period-cohort

## Abstract

**Background:**

Fungal skin diseases represent pervasive global health concerns, predominantly arising from dermatophytes, yeasts, and molds.

**Objective:**

This study aimed to estimate the disease burden associated with fungal skin diseases in 2021. Additionally, it sought to analyze trends from 1990 to 2021 and forecast future patterns.

**Methods:**

This observational study first utilized data from the Global Burden of Disease (GBD) database covering the years 1990 to 2021. We specifically used data from GBD 2021 to evaluate the global incidence, prevalence, and disability-adjusted life years (DALYs), disaggregated by age, gender, socio-demographic index (SDI), and GBD regions. Linear regression models were then employed to identify temporal trends, estimating the annual percentage change. Cluster analysis examined disparities across 45 GBD regions. To forecast future disease burden, we applied the age-period-cohort model and the autoregressive integrated moving average model.

**Conclusion:**

In 2021, there were approximately 1.73 billion global cases of fungal skin diseases. Males had higher age-standardized rates for incidence, prevalence, and DALYs compared to females. Age-specific analyses showed that although younger groups experienced the highest incidence rates, ASRs increased with age, especially among older populations. Regionally, low and middle SDI areas faced the greatest burden, with Asia having the highest incidence and Oceania the lowest. Projections suggest significant increases in incidence, prevalence, and DALYs, notably in middle- and low-income regions. These results highlight meaningful spatiotemporal disparities in fungal skin diseases and emphasize the need for strategic allocation of resources to mitigate these challenges and reduce the growing burden across various global populations.

## 1 Introduction

Fungal infections, specifically dermatomycoses and fungal skin diseases, form a distinct category of skin disorders primarily caused by dermatophytes, yeasts, and molds. As of 2017, these fungal skin conditions became the most prevalent skin disorders worldwide, representing 10.09% of all skin diseases (1, 2). In a 2016 global comparison of 328 diseases and injuries, fungal skin diseases were found to have the fourth highest incidence, with a staggering 2.1 billion cases reported. This prevalence underscores the significant socioeconomic impact they impose (2).

Fungal skin diseases are caused by diverse pathogens with distinct ecological niches. Superficial infections such as tinea corporis in tropical South Asia and Sub-Saharan Africa are predominantly driven by dermatophytes (3), which thrive in humid climates. In contrast, deep infections such as chromoblastomycosis in rural Brazil (4), and mycetoma in Sudan (5) are linked to soil-borne molds, reflecting occupational exposure to contaminated environments. High-income regions face unique challenges: *Candida auris* outbreaks in ICUs and azole-resistant *Aspergillus fumigatus* in agricultural settings underscore the role of healthcare and environmental selection pressures (6). Beyond geographic location and climate, other factors such as the host’s immune status (7), the pathogenicity of infectious agents (2, 8), and access to medical services also influence the incidence of fungal infections among populations (9).

Recent studies have highlighted the escalating threat of antifungal resistance. For instance, the emergence of *Trichophyton indotineae*—a terbinafine-resistant dermatophyte strain originating from South Asia—has led to near-epidemic outbreaks globally (10, 11). Concurrently, the global spread of *Candida auris* and azole-resistant *Aspergillus fumigatus* underscores the urgency of monitoring resistance patterns (12). Furthermore, host genetic predispositions (e.g., TLR2/4 polymorphisms) and immunological vulnerabilities (e.g., HIV coinfection or diabetes) have been increasingly recognized as critical modulators of disease susceptibility (7, 8). These factors collectively complicate therapeutic management and necessitate updated epidemiological frameworks.

Advances in diagnostic technologies, such as PCR-based assays and MALDI-TOF mass spectrometry, have improved species-level identification and antifungal susceptibility profiling (13, 14). However, these tools remain underutilized in high-burden regions, highlighting a critical implementation gap. Meanwhile, epidemiological studies have identified hotspots of fungal transmission linked to urbanization, overcrowding, and occupational exposures—factors increasingly relevant in rapidly developing economies (15–17). These studies highlight the profound implications these diseases have on public health, notably affecting daily life quality and imposing financial strains. The impact is often measured using disability-adjusted life years (DALYs), which combine the potential years lost to early mortality with years lived with disability. Additionally, the socio-demographic index (SDI) provides an encompassing metric of development by accounting for factors such as income, education, and fertility rates across various regions.

Despite progress in researching fungal skin diseases, several challenges remain unresolved. One major issue is the unequal representation in data collection globally, especially in low- and middle-income countries (18), hindering the accurate assessment of the global disease burden.

This study leverages updated GBD 2021 data to address these gaps, providing a comprehensive analysis of global trends from 1990 to 2021 and projecting disease trajectories through 2046. Previous studies have used Joinpoint regression analysis to review temporal trends in these diseases (19). Building on this work, we elucidate the evolving demographic and geographic patterns of fungal skin diseases by integrating age-period-cohort and ARIMA models, emphasizing the synergistic roles of socioeconomic inequity, environmental factors, and healthcare infrastructure. Our findings aim to inform targeted prevention strategies and advocate for equitable resource allocation in an era of escalating antifungal resistance and climatic volatility.

## 2 Materials and methods

### 2.1 Data source

This study utilizes data sourced from the GBD 2021 database. The GBD initiative, which commenced in 1991, has provided comprehensive empirical evaluations of global health statuses over the past 30 years (20, 21). Each iteration of the study has broadened in scope and intricacy, incorporating a wider array of causes and risks, expanding geographical coverage, and refining age-specific analyses. For the 2021 iteration, data from 328,938 unique sources were analyzed, resulting in the generation of over 60.7 billion estimates. These estimates addressed 25 distinct age groups, from birth to 95 years and older, across 204 countries and territories (22). The territories were further categorized into 21 geographic regions and stratified into five Socio-Demographic Index (SDI) categories—low, low-middle, middle, high-middle, and high SDI—as well as into seven broader super-regions. Separate estimates were generated for males, females, and overall populations by an international team of collaborators dedicated to rigorous data evaluation and analysis (23).

This section elaborates on the methodologies for data collection and estimation utilized by the GBD 2021. Detailed descriptions of these methodologies, including data search and selection processes, have been thoroughly documented in previous GBD publications. The procedures for data collection and analysis adhered closely to the Guidelines for Accurate and Transparent Health Estimates Reporting (GATHER) (24). The study employed DALYs to quantify the burden of diseases, calculated as the cumulative years lost to illness, disability, or premature mortality in a specific demographic.

The specific methodologies of GBD 2021, along with comprehensive analyses and model specifications, are detailed in other publications. The standard life expectancy utilized in this study was derived from the lowest age-specific mortality rates recorded in any country.

### 2.2 Statistical analysis

To analyze temporal trends in fungal skin diseases globally from 1990 to 2021, we estimated the Estimated Annual Percentage Change (EAPC). The detailed methodology for EAPC calculation has been extensively documented in previous studies. In summary, a regression model was employed to capture the yearly percentage variation in the Age-Standardized Rate (ASR) (25), using the natural logarithm of the fitted rate equation: y = *α* + *β*x + ɛ, where y denotes ln (ASR) and x represents the calendar year. The EAPC of ASR is computed as 100 × (exp(β) - 1). These analyses were conducted using the R programming language (R Core Team, version 3.5.3, Vienna, Austria), with statistical significance assessed at a two-sided *p*-value threshold of < 0.05 (26).

We implemented an Age-Period-Cohort (APC) model to forecast the burden of fungal skin diseases (incidence, prevalence, and DALYs) across age groups from 2022 to 2046. This approach quantifies three temporal drivers: (1) age effects (biological susceptibility and lifetime exposure accumulation), (2) period effects (population-level changes like diagnostic guidelines revisions), and (3) cohort effects (generational risks from early-life antifungal stewardship programmes) (27). The log-linear model was specified as: Y = *μ* + *α*(age) + *β*(period) + *γ*(cohort) + *ε*, where α, β, γ represent partial regression coefficients for each temporal dimension, μ is the intercept, and ε accommodates overdispersion in Poisson-distributed outcomes (28).

For sensitivity analysis, we contrasted APC projections with seasonal Autoregressive Integrated Moving Average (ARIMA) Model (1, 2) forecasts (29), with autoregressive (lag = 2) and moving average (lag = 1) terms selected via autocorrelation function minimization (30).

## 3 Results

### 3.1 Global disease burden attributable to fungal skin diseases in 2021

In 2021, there were approximately 1,729,224,009 cases of fungal skin diseases (95% uncertainty intervals (UI): 1,562,709,709 to 1,894,666,053) (Table 1). Notably, the incidence rates were similar across both genders, as illustrated in Supplementary Figure S1. However, an examination of prevalence and DALYs revealed that males experienced higher values than females in terms of absolute case numbers and ASRs. Specifically, incidence, prevalence, and DALYs were 1.01, 1.07, and 1.09 times greater in males compared to females. Likewise, the ASIR ASPR and ASDR were 1.06, 1.11, and 1.12 times higher in males, respectively.

**Table 1:** Temporal trends of fungal skin diseases by gender, SDI and GBD regions, 1990 to 2021.

Characteristics	2021	Prevalence			DALYs		1990–2021		
	Incidence						ASIR	ASPR	ASDR
	Numbers (95% UI)	ASR	Numbers (95% UI)	ASR	Numbers (95% UI)	ASR	(95% CI)	(95% CI)	(95% CI)
		No. ×10–5 (95%UI)		No. ×10–5 (95%UI)		No. ×10–5 (95%UI)	EAPC_CI	EAPC_CI	EAPC_CI
Global									
Both	1,729,224,009 (1562709709–1,894,666,053)	21668.4 (19601.19–23729.17)	616,532,287 (558724637–679,274,726)	7789.55 (7059.28–8583.54)	3,429,497 (1407894–7,044,259)	43.39 (17.79–89.1)	0.11 (0.1–0.12)	0.19 (0.18–0.21)	0.21 (0.19–0.22)
Female	858,570,870 (775052838–940,484,924)	21066.02 (19076.14–23048.66)	297,390,154 (269989615–326,373,246)	7376.39 (6691.79–8118.92)	1,642,990 (675685–3,361,323)	40.92 (16.78–83.77)	0.11 (0.1–0.11)	0.17 (0.16–0.18)	0.19 (0.18–0.2)
Male	870,653,138 (786153592–954,661,669)	22238.63 (20103.72–24325.69)	319,142,132 (287775972–352,445,711)	8188.82 (7401.08–9037.07)	1,786,507 (732065–3,683,464)	45.8 (18.77–94.26)	0.12 (0.11–0.13)	0.21 (0.19–0.22)	0.22 (0.2–0.24)
SDI region									
High-middle SDI	260,188,514 (234220160–287,174,277)	16920.08 (15266.5–18644.11)	81,602,182 (74395167–90,346,743)	5354.46 (4863.19–5911.78)	448,114 (183348–921,094)	29.66 (12.03–61.42)	−0.17 (−0.18--0.17)	−0.16 (−0.16−−0.15)	−0.15 (−0.15--0.14)
High SDI	243,866,476 (220333079–269,297,424)	16147.92 (14687.3–17628.18)	77,663,743 (71961405–85,232,930)	5117.27 (4725.72–5561.42)	420,160 (173757–852,117)	28.19 (11.64–57.15)	-0.15 (−0.15--0.14)	−0.16 (−0.17--0.16)	−0.17 (−0.17--0.16)
Low-middle SDI	394,534,381 (353702982–434,592,656)	22004.64 (19797.44–24106.7)	140,711,272 (125727190–157,977,912)	7731.22 (6958.71–8612.32)	787,326 (322644–1,623,830)	42.94 (17.65–88.57)	0.01 (0–0.01)	0.01 (0.01–0.02)	0.02 (0.02–0.03)
Low SDI	325,796,367 (287974642–368,388,493)	31124.75 (27943.94–34383.12)	142,839,638 (123058640–163,253,386)	12866.92 (11521.34–14298.53)	807,825 (331736–1,696,213)	71.8 (29.26–149.06)	−0.05 (−0.07--0.04)	−0.18 (−0.19−−0.16)	-0.16 (−0.18--0.15)
Middle SDI	503,290,634 (454024197–554,675,359)	20125.73 (18206.98–22198.06)	173,183,772 (155963056–192,835,881)	6941.77 (6276.73–7692.35)	963,136 (393633–1,990,445)	38.62 (15.78–79.68)	0.03 (0.02–0.04)	0.05 (0.04–0.06)	0.06 (0.05–0.06)
Regions									
High-income Asia Pacific	58,497,384 (51388140–65,973,659)	19223.06 (17249.74–21450.51)	18,137,114 (16398804–20,304,408)	5921.2 (5359.76–6562.53)	97,977 (40319–197,988)	32.82 (13.36–66.18)	−0.04 (−0.04--0.04)	−0.05 (−0.06−−0.05)	-0.05 (−0.05--0.04)
Central Asia	15,899,318 (14300610–17,629,393)	18048.07 (16114.29–19997.58)	4,840,575 (4368567–5,413,076)	5502.46 (4989.41–6130.45)	26,874 (10846–55,460)	30.35 (12.34–61.99)	0 (0–0)	0 (0–0)	0 (0–0)
East Asia	236,950,733 (212978978–263,127,313)	14227.76 (12819.42–15818.22)	71,809,813 (64761791–79,801,046)	4346.89 (3907.34–4830.3)	398,723 (162212–830,635)	24.28 (9.83–50.71)	−0.15 (−0.15--0.14)	−0.18 (−0.19--0.17)	−0.18 (−0.19--0.17)
South Asia	340,937,797 (303802441–377,408,907)	19696.89 (17580.28–21752.46)	112,096,656 (98968557–126,418,264)	6425.74 (5692.87–7177.08)	624,823 (254045–1,282,714)	35.59 (14.54–73.31)	−0.11 (−0.11--0.1)	−0.18 (−0.19--0.17)	−0.18 (−0.19--0.17)
Southeast Asia	193,137,286 (172966376–215,997,244)	27579.96 (24668.34–30727.02)	76,956,034 (68120672–86,649,851)	10853.49 (9663.93–12128.52)	431,548 (172611–897,329)	60.72 (24.38–125.8)	−0.02 (−0.02−−0.02)	-0.02 (−0.02--0.02)	−0.01 (−0.02--0.01)
Australasia	9,670,089 (8787276–10,688,314)	24492.01 (22211.11–26796.32)	3,551,173 (3401679–3,705,444)	8674.33 (8253.11–9133.01)	19,229 (7927–38,968)	47.67 (19.51–98.06)	−0.02 (−0.03−−0.02)	−0.02 (−0.02--0.02)	-0.02 (−0.02--0.02)
Caribbean	12,791,954 (11443688–14,243,213)	25655.03 (22966.31–28573.75)	4,231,139 (3838371–4,683,834)	8490.95 (7688.73–9420.81)	23,271 (9624–48,117)	46.84 (19.43–97.05)	−0.01 (−0.01−−0.01)	−0.01 (−0.01–0)	-0.01 (−0.02--0.01)
Central Europe	28,988,989 (25732360–32,200,694)	18152.72 (16232.24–20130.03)	8,797,948 (8015062–9,865,405)	5547.72 (5046.84–6164.82)	47,583 (19403–96,013)	30.52 (12.41–62.55)	0 (0–0)	0 (0–0)	0.01 (0.01–0.01)
Eastern Europe	48,985,877 (43277150–54,790,606)	18374.98 (16424.36–20390.18)	14,925,815 (13583984–16,793,845)	5640.64 (5132.74–6279.89)	80,814 (32830–163,988)	30.95 (12.69–63.75)	0 (0–0)	0 (0–0)	0.01 (0–0.01)
Western Europe	143,367,679 (127506138–160,700,549)	23177.13 (20787.65–25596.06)	47,550,475 (43317751–53,001,043)	7634.36 (6923.31–8402.41)	257,296 (106636–533,552)	42.18 (17.37–86.38)	−0.03 (−0.03−−0.02)	−0.02 (−0.03--0.01)	-0.02 (−0.02--0.01)
Andean Latin America	19,833,548 (17793929–22,026,926)	30930.16 (27772.91–34537.99)	7,803,891 (6972788–8,667,501)	12185.3 (10928.53–13484.34)	43,352 (17759–89,416)	67.51 (27.8–139.33)	−0.05 (−0.05−−0.05)	−0.05 (−0.06--0.05)	-0.05 (−0.05--0.05)
Central Latin America	47,243,257 (42479108–52,194,135)	18823.54 (16934.01–20811.19)	14,252,467 (12956015–15,754,044)	5684.18 (5172.88–6293.48)	78,631 (32252–162,385)	31.33 (12.92–64.32)	0.08 (0.02–0.15)	0.1 (0.02–0.17)	0.1 (0.02–0.17)
Southern Latin America	14,555,969 (12965731–16,156,392)	19065.44 (17057–21112.96)	4,460,228 (4044420–4,938,312)	5844.86 (5296.87–6447.14)	24,408 (10057–49,802)	32.18 (13.23–65.53)	−0.04 (−0.04--0.03)	−0.05 (−0.05--0.04)	−0.05 (−0.06--0.05)
Tropical Latin America	62,653,211 (56393206–69,558,553)	26302.46 (23768.2–29078.3)	20,410,381 (18525634–22,553,587)	8597.1 (7778.7–9470.52)	111,686 (46262–230,126)	47.16 (19.61–96.35)	−0.03 (−0.03--0.03)	−0.04 (−0.04--0.04)	−0.03 (−0.04--0.03)
North Africa and Middle East	59,785,973 (53911475–65,710,812)	11122.47 (10049.39–12248.76)	17,854,562 (16208416–19,697,750)	3311.44 (3029.03–3657.63)	98,647 (40348–201,213)	18.09 (7.39–36.68)	−0.09 (−0.1--0.09)	−0.12 (−0.13--0.11)	−0.13 (−0.14--0.12)
High-income North America	41,256,177 (38055027–44,314,078)	8438.69 (7866.31–8989.68)	12,239,743 (11750935–12,805,812)	2506.94 (2407.04–2606.08)	65,545 (27199–132,567)	13.67 (5.6–28.02)	−0.01 (−0.03–0)	−0.04 (−0.05--0.03)	−0.06 (−0.08--0.05)
Oceania	2,329,919 (2081626–2,608,322)	19063.29 (17141.24–21170.12)	739,478 (652407–832,720)	5973.01 (5353.77–6665.95)	4,155 (1687–8,565)	33.16 (13.45–68.35)	0 (0–0)	0 (0–0.01)	0.01 (0.01–0.01)
Central Sub-Saharan Africa	37,889,970 (32068840–44,945,141)	29742.7 (26265.95–33437.93)	17,734,503 (13834922–21,856,841)	12157.24 (10215.64–14241.13)	100,816 (41085–218,359)	68.05 (27.91–145.3)	−0.12 (−0.17--0.08)	−0.44 (−0.47--0.4)	−0.42 (−0.45--0.39)
Eastern Sub-Saharan Africa	153,079,011 (134806215–174,216,560)	38643.84 (34630.18–42795.3)	68,902,444 (59550670–78,967,916)	16407.63 (14685.94–18312.69)	390,277 (162032–811,951)	91.58 (37.52–191.62)	−0.11 (−0.12--0.1)	−0.3 \(−0.32--0.28)	−0.29 (−0.31--0.27)
Southern Sub-Saharan Africa	21,931,573 (19503932–24,407,963)	29868.53 (26669.82–33040.36)	7,487,199 (6627186–8,370,290)	10149.03 (9087.13–11253.98)	41,566 (16769–86,104)	55.83 (22.63–115.35)	−0.02 (−0.04–0)	−0.02 (−0.04--0.01)	−0.04 (−0.06--0.02)
Western Sub-Saharan Africa	179,438,295 (158387265–202,499,099)	39705.22 (35574.63–43841.03)	81,750,648 (71321336–93,056,770)	17516.41 (15769.24–19474.17)	462,278 (188745–975,268)	97.72 (40.06–202.83)	−0.04 (−0.06--0.03)	−0.09 (−0.11−−0.07)	-0.07 (−0.09--0.05)

No., number; DALYs, disability-adjusted-life-years; ASIR, age-standardized incidence rate; ASPR, age-standardized prevalence rate; ASDR, age-standardized DALY rate; UI: uncertainty intervals; CI: confidence interval; SDI: socio-demographic index; GBD: global burden of diseases; EAPC: estimated annual percentage change.

In terms of age distribution, the global ASRs demonstrated a notable increase across age groups ranging from 25 to 95+, peaking in the 95 + age group with an ASR for DALYs of 232.32 per 100,000 (95% uncertainty interval [UI]: 90.88–497.8) in 2021. Conversely, for individuals from birth to 24 years old, the global age-standardized rates (ASRs) initially rose, peaking in the 5–9 age group, and then began to decrease.

**Figure 1:**
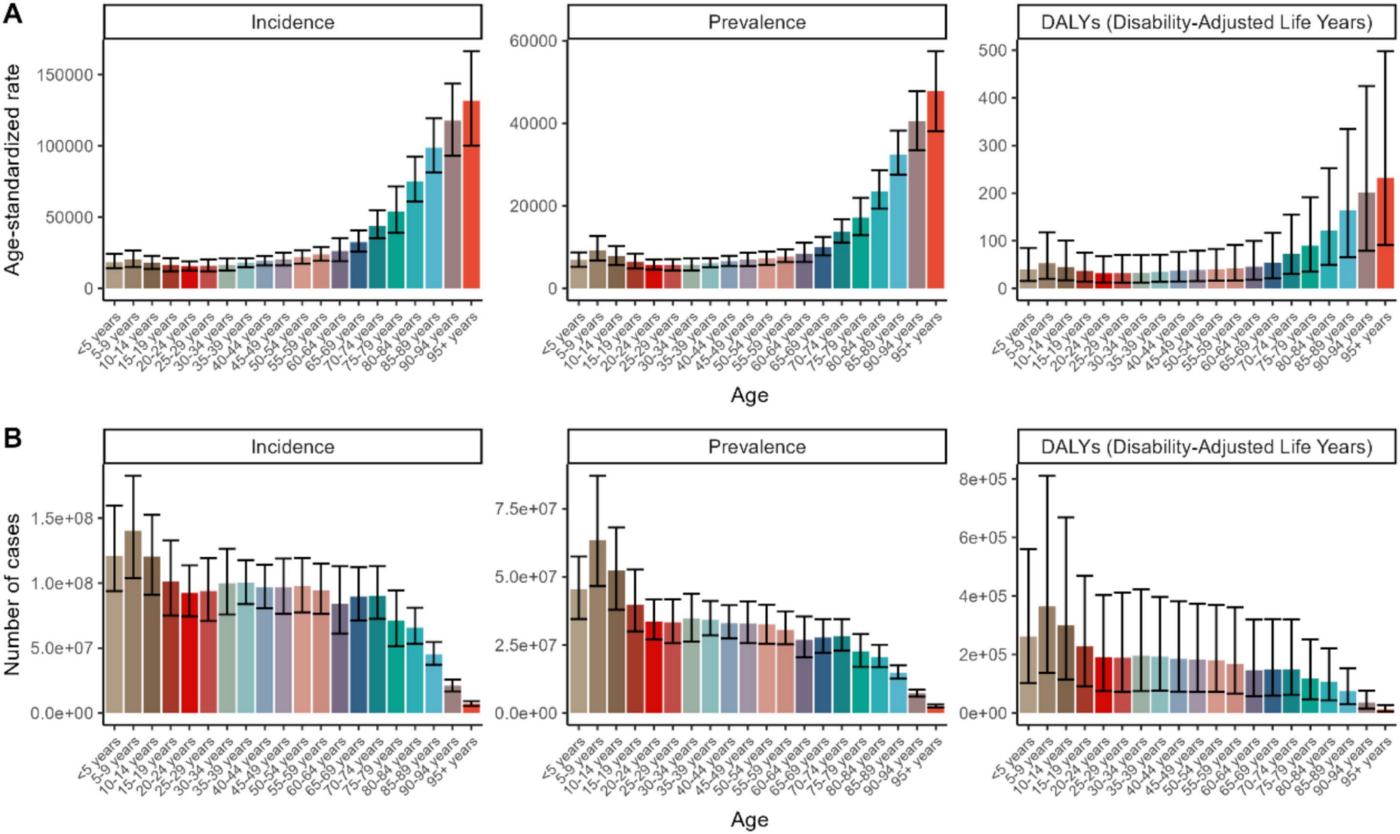
The age distribution of ASRs **(A)** and numbers **(B)** of fungal skin diseases-related incidence, prevalence and DALYs in 2021.

However, when considering the number of cases, a different pattern emerged. The younger age group (5–9 years) exhibited the highest incidence (140,377,507 cases, 95% UI: 103,902,413-182,566,892) and prevalence (63,559,849 cases, 95% UI: 46,563,538-87,068,326), with a gradual decrease observed in older age groups. This trend was also reflected in the DALYs, which were significantly higher among the younger demographic (Figure 1).

At the SDI region level, the high SDI regions exhibited the lowest ASRs for incidence and prevalence in 2021, with values of 16,148 (95% uncertainty interval [UI]: 14,687-17,628) and 5,117 (95% UI: 4,726-5,561) per 100,000, respectively. However, the ASRs increased significantly towards the low SDI regions. A similar pattern was observed for DALYs, with the highest burden noted in the low SDI regions (ASR of 71.8, 95% UI: 29.26–149.06) (Table 1).

The middle SDI region reported the highest number of incidence cases at 503,290,634 (95% UI: 454,024,197-554,675,359), prevalence cases at 173,183,772 (95% UI: 155,963,056-192,835,881), and DALYs cases at 963,136 (95% UI: 393,633-1,990,445). Conversely, the high SDI region had the lowest number of cases, followed by the high-middle SDI region. Notably, the low SDI region had fewer incidence cases but more DALYs and prevalence cases compared to the low-middle SDI region.

**Figure 2:**
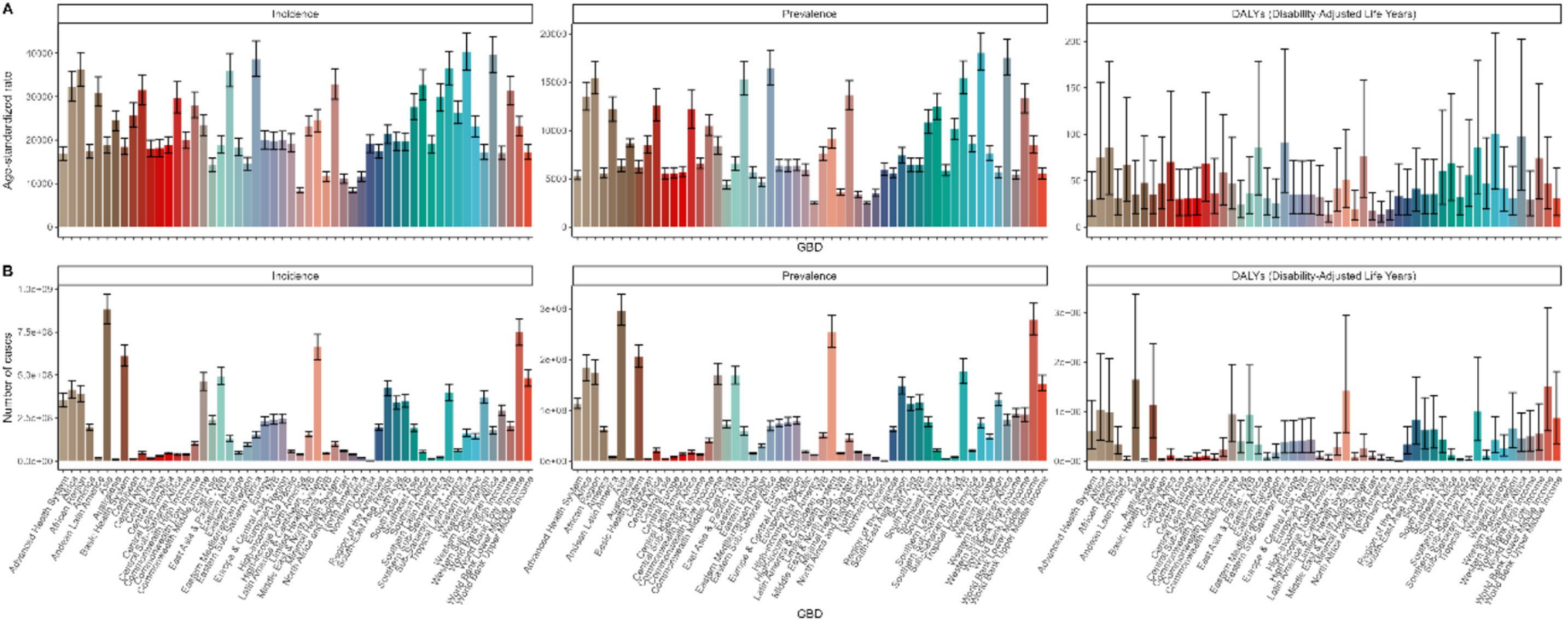
GBD Region-specific analysis of fungal skin diseases burden in 2021. ASRs **(A)** and number of cases **(B)** of incidence, prevalence, and DALYs across different GBD regions.

Among the 45 GBD regions in 2021, Asia ranked first in terms of fungal skin disease-related incidence (883,292,233, 95% uncertainty interval [UI]: 796,351,242-972,024,863), prevalence (296,194,797, 95% UI: 266,859,176-329,067,591), and DALYs (1,647,941, 95% UI: 675,987-3,379,970). Conversely, Oceania ranked last for incidence (2,329,919, 95% UI: 2,081,626-2,608,322), prevalence (739,478, 95% UI: 652,407-832,720), and DALYs (4,155, 95% UI: 1,687-8,565) (Figure 2).

Regarding the corresponding ASRs, Western Africa emerged as the top GBD region for incidence (40,345, 95% UI: 36,125-44,592), prevalence (18,033, 95% UI: 16,222-20,064), and DALYs (101, 95% UI: 41–209). In contrast, High-income North America ranked lowest for incidence (8,439, 95% UI: 7,866-8,990), prevalence (2,506.94, 95% UI: 2,407-2,606), and DALYs (13.67, 95% UI: 6–28). Further, Western Sub-Saharan Africa and Eastern Sub-Saharan Africa reported elevated incidence and DALYs compared to most other GBD regions. Additionally, Sub-Saharan Africa – WB and Eastern Sub-Saharan Africa exhibited higher prevalence rates than the majority of other GBD regions.

### 3.2 Global distribution in 2021

Supplementary Table S1 provides the global distribution of fungal skin diseases varied significantly across different regions and health metrics in 2021. Ethiopia reported the highest ASIR of 47,245 (95% uncertainty interval [UI]: 42,242-52,965), followed by Nigeria and Mali. Additionally, Mali exhibited the highest ASRs for prevalence and DALYs, with values of 23,697 (95% UI: 20,973-26,339) and 133 (95% UI: 54–274), respectively.

In 2021, the United States recorded the lowest ASRs for both incidence (8,401; 95%UI: 7,814–8,916) and disability-adjusted life years (DALYs) (14; 95% UI: 6–28), with Canada and Greenland following. For prevalence, Canada had the lowest ASR (2,490; 95% UI: 2,245–2,782), followed by the United States. Contrastingly, India reported the highest numbers in 2019 for incidence (265,162,118; 95% UI: 235,600,132–294,116,264), prevalence (87,230,762; 95% UI: 77,093,668–98,204,047), and DALYs (485,519; 95% UI: 197,480–994,783), with China and Nigeria next in line. Remarkably, the numbers for incidence, prevalence, and DALYs cases were nearly negligible in Tokelau, followed by Niue.

### 3.3 Global trends from 1990 to 2021

Supplementary Figure S2 presents the results of a cluster analysis based on the EAPC values of ASIR, ASPR and ASDR of fungal skin diseases from 1990 to 2021. The analysis reveals distinct trends among various regions.

Specifically, regions such as Southern Africa, Western Europe, Southern Sub-Saharan Africa, and North America exhibit significant decreases in ASRs, denoted by purple branches. In contrast, several regions, including Eastern Sub-Saharan Africa, Eastern Africa, Central Africa, and Central Sub-Saharan Africa, demonstrate significant increases, indicated by blue branches. Despite these notable variances, regions like the Americas (including both North and South America) show localized stability or minor decreases, as highlighted in green.

The majority of regions worldwide exhibit an increase ranging from 0 to 200% across all three metrics, as predominantly indicated by the light blue shading in all maps (Supplementary Figure S3). Notable exceptions to this general trend include Eastern Europe and parts of Southern Africa. Specifically, Eastern Europe demonstrates substantial decreases in these metrics, while parts of Southern Africa show increases of up to 600–800%.

**Figure 3:**
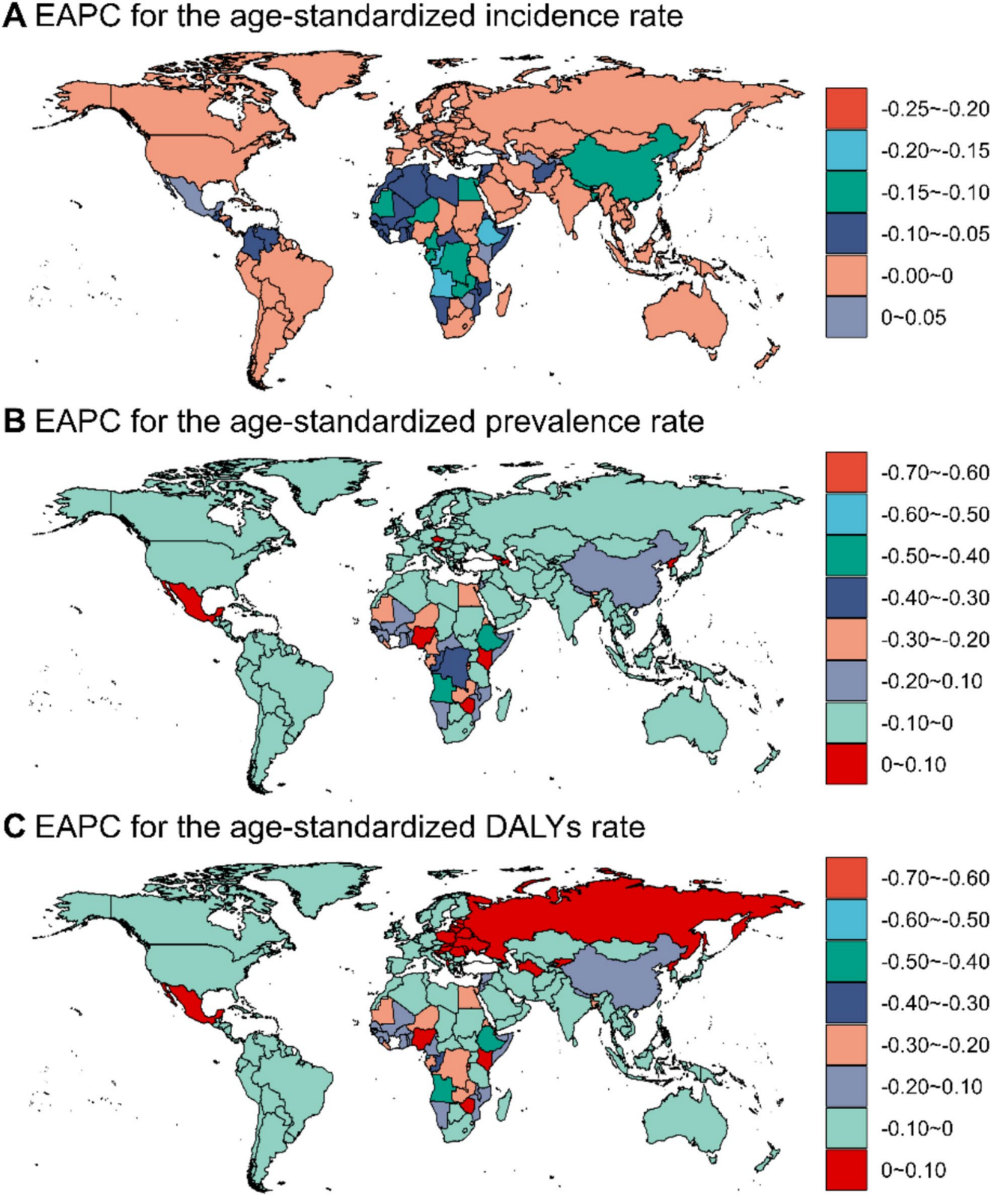
The global distribution of EAPCs for age-standardized incidence, prevalence and DALYs rates of fungal skin diseases from 1990 to 2021. **(A)** ASIR; **(B)** ASPR; **(C)** ASDR.

Figure 3 and Table 1 illustrates the global distribution of estimated annual percentage changes (EAPCs) for ASIR, ASPR, and ASDR of fungal skin diseases from 1990 to 2021.

Figure 3A displays significant regional variability, with notable declines observed in parts of North America, Europe, and East Asia. Conversely, several African and South American nations exhibit increases in ASIR. Figure 3B (ASPR) generally shows a more widespread decline across various regions, including North America, parts of Europe, and regions in Asia, with isolated increases noted in certain African and Latin American countries. In contrast, Figure 3C (ASDR) highlights substantial increases in Eastern Europe and parts of Central Asia, which contrast sharply with decreases observed in other global regions.

### 3.4 Prediction of future trends of fungal skin diseases

According to projections using the APC model for 2022–2046, both males and females are expected to see a rise in the number of incidence, prevalence, and DALYs cases. This increase is accompanied by a corresponding growth in ASRs across all categories. Specifically, the anticipated growth rates for incidence, prevalence, and DALYs cases are 34.3, 32.0, and 30.1%, respectively. There are distinct gender-specific trends observed, with males consistently displaying higher ASRs across all metrics compared to females.

**Figure 4:**
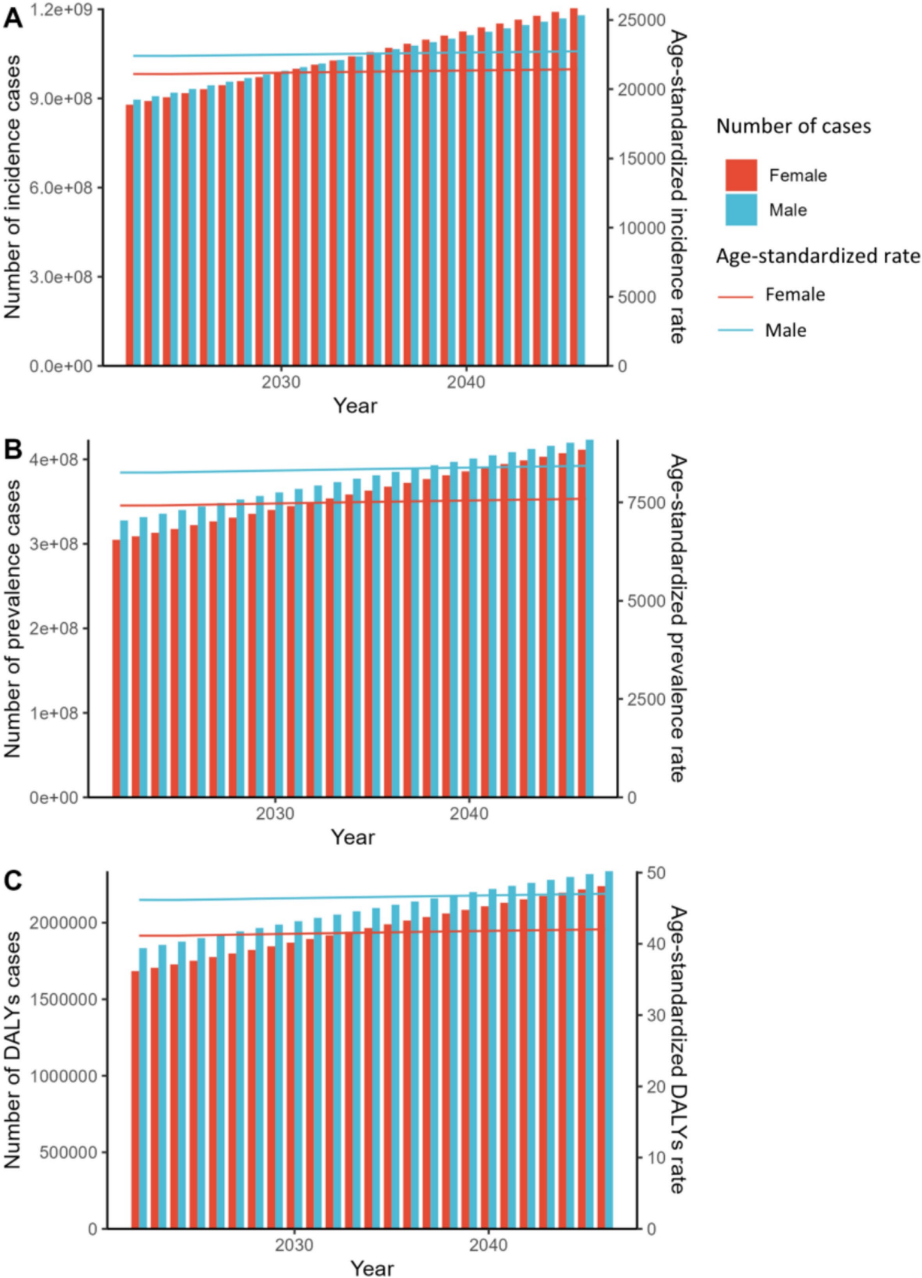
Prediction by age-Period-Cohort (APC) model for global trends in ASR and number of cases of fungal skin diseases from 2022 to 2046. **(A)** ASIR and number of incidence cases; **(B)** ASR and number of prevalence cases; **(C)** ASDR and number of DALYs cases.

According to Figure 4, the number of prevalence and DALYs cases is projected to continue being higher in males than in females in the future. Specifically, the number of prevalence and DALYs cases is forecasted to increase by 35.0 and 33.0% for females, and by 29.1 and 27.4% for males, respectively. Notably, females are expected to experience a faster growth rate in the number of prevalence and DALYs cases, outpacing that of males. However, the trend in incidence cases will be more intricate. From 2022 to 2033, more incidence cases are forecasted in males than in females, but this trend is expected to reverse from 2034 to 2046. In general, females are expected to see a faster relative increase in incidence cases by 37.0% compared to males (31.7%) during this timeframe.

**Figure 5:**
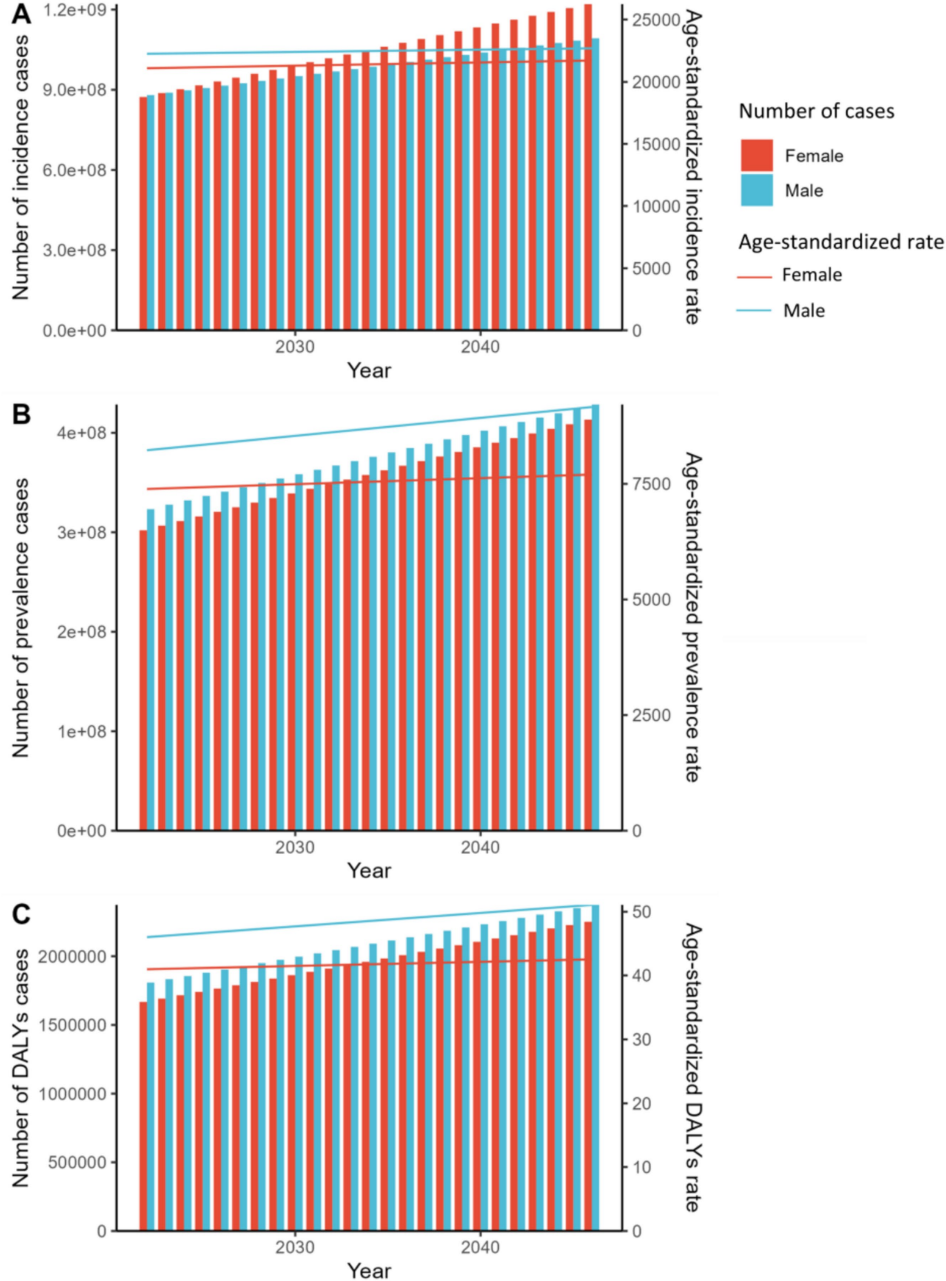
Prediction by ARIMA Model for global trends in ASR and number of cases of fungal skin diseases from 2022 to 2046. **(A)** ASIR and number of incidence cases; **(B)** ASR and number of prevalence cases; **(C)** ASDR and number of DALYs cases.

In line with the APC model findings, the ARIMA model also predicts an upward trend in incidence, prevalence, and DALYs cases for both genders, as well as corresponding ASRs (Figure 5). Globally, incidence and DALYs cases are estimated to rise by 37.2 and 38.6% from 2022 to 2050. Unlike the APC model’s predictions, the ARIMA model forecasts that the number of incidence cases in females will exceed that of males by 2024. Moreover, prevalence cases are anticipated to increase by 40.4%, predominantly impacting males.

## 4 Discussion

This study comprehensively analyzes the global burden of fungal skin disease from 1990 to 2021, highlighting an increasing trend of worldwide fungal skin disease, accounting for approximately 8% of total DALYs owing to skin disease.

The increasing global burden of fungal skin diseases over the past three decades stems from multifaceted environmental, demographic, and healthcare challenges. Climate change has created favorable conditions for fungal proliferation and adaptation, with rising temperatures and humidity enhancing pathogen survival and resistance mechanisms (31). Demographic shifts, particularly population aging in regions like East Asia, have amplified susceptibility due to age-related immune decline and comorbidities (32). Healthcare disparities persist in low-resource settings, where limited diagnostic capacity and fragmented surveillance systems delay detection and exacerbate transmission (33). Rapid urbanization and population growth have intensified overcrowded living conditions, facilitating fungal spread in densely populated areas (34). Concurrently, expanding immunocompromised populations, driven by HIV/AIDS and immunosuppressive therapies, have heightened vulnerability to severe and opportunistic fungal infections (4). Paradoxically, hygiene practices, including prolonged use of protective equipment during the COVID-19 pandemic, disrupted skin integrity, increasing risks of superficial infections (35). Diagnostic and therapeutic gaps remain critical, as inadequate access to mycological testing and emerging antifungal resistance undermine treatment efficacy (33). Finally, ecological disruptions, such as pollution-induced microbiome alterations and zoonotic spillover risks, underscore evolving interactions between pathogens, hosts, and environments (36).

Fungal skin diseases result from a complex interplay of pathogen virulence, host susceptibility, and environmental factors. Host genetic factors play a crucial role in infection outcomes. Our study shows gender-specific disparities in ASIR, potentially linked to biological factors, such as androgen-influenced sebum composition, and behavioral risks (37), like occupational exposure in males (38). Environmental conditions also influence disease patterns—tropical climates support thermophilic species such as *Aspergillus fumigatus* (39), whereas overcrowding in low-SDI regions promotes the spread of anthropophilic species like Trichophyton tonsurans (40). Importantly, climate change is altering fungal distributions, for instance, the suitable habitats of *Colletotrichum* spp. expand northward with rising temperatures in southern China, which may indirectly increase the risk of human contact - based skin infections through agricultural activities (31, 41).

In 2021, males exhibited higher ASIR, ASPR and ASDR compared to females, likely driven by occupational exposures, hormonal influences, and skin physiology, emphasizing the need for gender-sensitive prevention strategies. Age-stratified analyses further identify distinct risk profiles: the older adult (≥80 years) experience disproportionately high burden due to immunosenescence and comorbidities, while children under 9 years old face early-life susceptibility linked to hygiene practices and immature immune defenses. These dual demographic peaks underscore the necessity of age-tailored interventions, ranging from microbiome-modulating probiotics in geriatric care to school-based screening programs in pediatric populations.

The global burden of fungal skin diseases demonstrates distinct regional patterns shaped by climatic and socioeconomic drivers. Although tropical climates in South Asia and Sub-Saharan Africa naturally favor fungal growth through sustained humidity, our analysis identifies poverty-related factors as equally critical. Low SDI regions exhibit the highest ASRs, underscoring how poverty amplifies exposure through overcrowded living conditions, inadequate footwear usage, and limited access to clean water and healthcare (33). South Asia and Sub-Saharan Africa’s surveillance gaps likely mask true disease prevalence, as resource-limited systems prioritize acute infections over dermatological monitoring—a cycle that perpetuates underfunding of skin health programs (42). Cultural practices like shared bathing facilities may further interact with environmental and economic factors to sustain transmission (43). Middle-SDI regions bear the greatest absolute caseload, driven by rapid urbanization concentrating populations in informal settlements with poor sanitation, combined with healthcare systems that enable passive case reporting without effective interventions (44).

While Sub-Saharan Africa is emblematic of the interplay between poverty and fungal burden, the continent’s cultural and ecological diversity demands a nuanced subregional perspective. In general, tinea capitis, tinea corporis, tinea pedis, and onychomycosis are the main manifestations, and immunosuppressed patients (e.g., HIV-infected patients) are prone to develop severe infections. In sub-Saharan Africa, where HIV is highly prevalent, the problem of dual infection is a concern (45), in addition, tinea capitis is high in school-age children in this area (46), and the impact of forest degradation and wood fuel use on sanitary conditions exacerbates the risk of infection (47); North Africa has a high incidence but limited diagnostic resources; In places such as Ethiopia in East Africa, the high infection rate is closely related to hot and humid environments, poor sanitation and animal contact (48). In South Africa, tinea capitis is the most common subtype, followed by tinea corporis and onychomycosis (43), Terbinafine-resistant strains of ringworm have been reported in parts of South Africa, leading to an increase in cases of refractory infection (49).

Effective mitigation requires region-specific strategies that bridge biomedical and structural interventions. In low-SDI settings, scaling up point-of-care diagnostics and community-led hygiene initiatives could reduce transmission. Moreover, leveraging digital health tools for improved disease monitoring and resource allocation can help bridge existing healthcare gaps, particularly in high-prevalence, resource-constrained regions. High-SDI regions can prioritize antifungal stewardship and hospital decontamination protocols to curb nosocomial outbreaks. Emerging therapies, such as antifungal peptides and nanoparticle-based drug delivery systems enhancing drug penetration, offer promise but require validation in real-world settings (50, 51).

This study’s limitations, including the aggregation of superficial and deep fungal infections, highlight the need for pathogen-specific subtyping in future research. Molecular tools like whole-genome sequencing could disentangle the epidemiology of dermatophytes versus invasive molds, as demonstrated in recent clade analyses of *C. auris* (52). Furthermore, the studies available for comparison across various GBD regions might be limited by the geographic scope, leading to certain populations being over- or under-represented relative to their actual population size. Additionally, the ability to report on the worldwide incidence of fungal diseases might be hindered by factors such as the absence of mandatory reporting for these diseases, suboptimal performance of diagnostic tests, and low levels of clinician awareness in particular areas. Future research should aim to bridge this gap, ensuring more comprehensive data collection in regions heavily burdened by fungal skin diseases. Longitudinal studies assessing climate-driven shifts in fungal biogeography and resistance patterns will be critical to informing adaptive public health policies.

## 5 Conclusion

This study demonstrates an escalating global burden of fungal skin diseases, characterized by significant projections of increased incidence, prevalence, and DALYs until 2046. These findings emphasize the imperative need for intensified global health strategies, particularly in regions with low SDI and tropical areas. Targeted interventions for older adults and very young individuals, as well as strategic resource allocation, are crucial to mitigate the growing impact of fungal skin diseases.

## Data Availability

Publicly available datasets were analyzed in this study. This data can be found here: https://vizhub.healthdata.org/gbd-results/.

## Glossary

GBD: Global Burden of Diseases
DALYs: Disability-Adjusted Life Years
ASR: Age-Standardized Rate
ASIR: Age-Standardized Incidence Rate. This is the number of new cases of a disease in a standardized population, adjusted for age differences. It allows for comparisons of how often a disease occurs between populations with different age structures, usually expressed per 100,000 people
ASPR: Age-Standardized Prevalence Rate. This shows the proportion of people affected by a disease at a specific point in time, adjusted for age. It includes both new and existing cases, and is also measured per 100,000 people, which helps compare how widespread a disease is across populations of varying age structures
ASDR: Age-Standardized DALY Rate. This represents the rate of Disability-Adjusted Life Years (DALYs), which combines the years lost due to early death and the years lived with disability from a disease. It is age-adjusted and expressed per 100,000 people, giving an estimate of the overall burden of the disease in different populations
SDI: Socio-Demographic Index
GATHER: the Guidelines for Accurate and Transparent Health Estimates Reporting
APC: Age-Period-Cohort
ARIMA: Autoregressive Integrated Moving Average. An autoregressive integrated moving average model is a form of regression analysis that gauges the strength of one dependent variable relative to other changing variables. The model’s goal is to predict future securities or financial market moves by examining the differences between values in the series instead of through actual values.
EAPC: Estimated Annual Percentage Change
CI: Confidence Interval
UI: Uncertainty Interval
