# Supplementary material for: Worldwide trends and future projections of fungal skin disease burden: a comprehensive analysis from the Global Burden of Diseases study 2021
Source: Front Public Health. 2025 Jun 4;13:1580221. doi: 10.3389/fpubh.2025.1580221 (PMC12174046; doi:10.3389/fpubh.2025.1580221)
Supplement: Supplementary file 1 [file Data_Sheet_1.ZIP › Supplementary information/Supplementary Figures and Tables-fungal.docx]

**Supplementary Figures and Tables**

**Worldwide Trends and Future Projections of Fungal Skin Disease Burden: A Comprehensive Analysis from the Global Burden of Diseases Study 2021**

**Supplementary Figures**

**Supplementary Figure S1** Numbers and ASRs of incidence, prevalence and DALYs attributable to fungal skin diseases by sex in 2021.

**
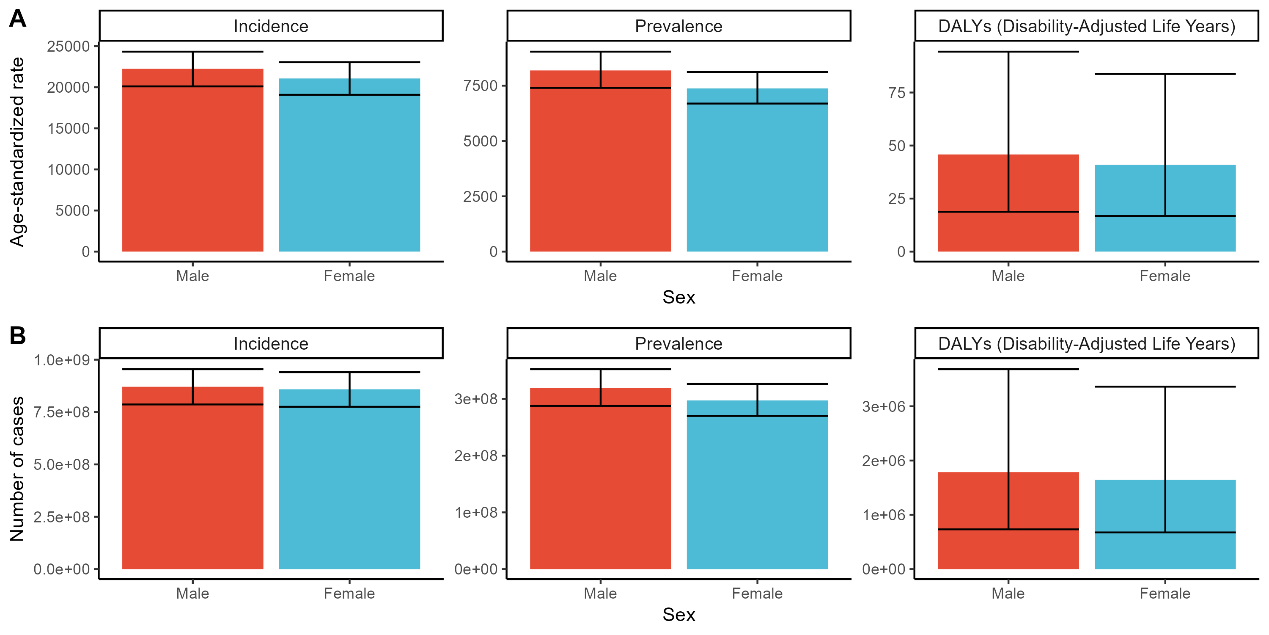
**

**Supplementary Figure S2** Cluster analysis of ASRs for incidence, prevalence and DALYs of fungal skin diseases from 1990 to 2021 based on the EAPC values. Trend patterns categorize regions: significant increase (blue), minor increase (purple), stable or minor decrease (green), and considerable decrease (red).

**
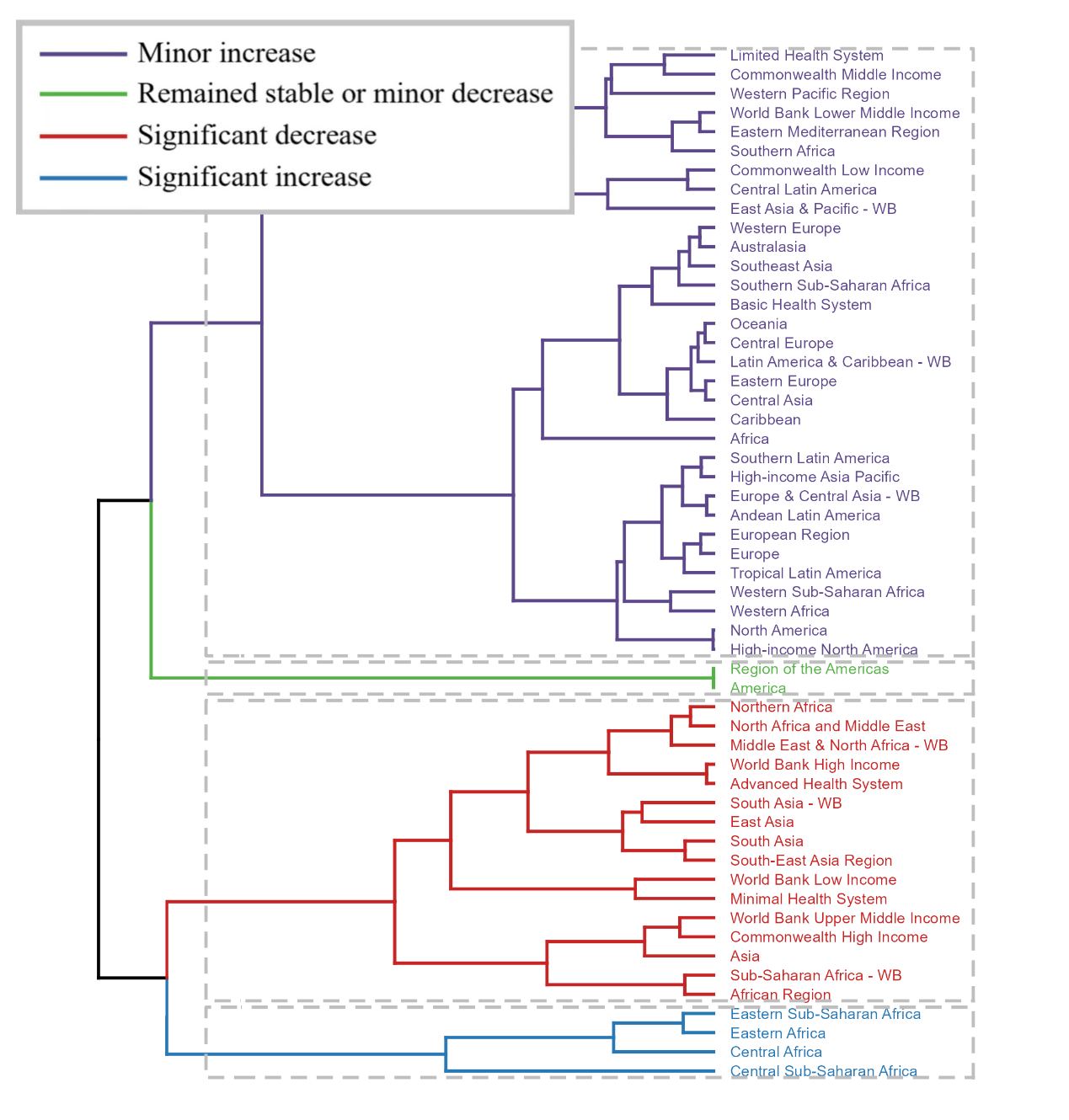
**

**Supplementary Figure S3** Changes in incidence, prevalence and DALYs cases of fungal skin diseases across countries and territories from 1990 to 2021. (A) incidence; (B) prevalence; (C) DALYs.

**
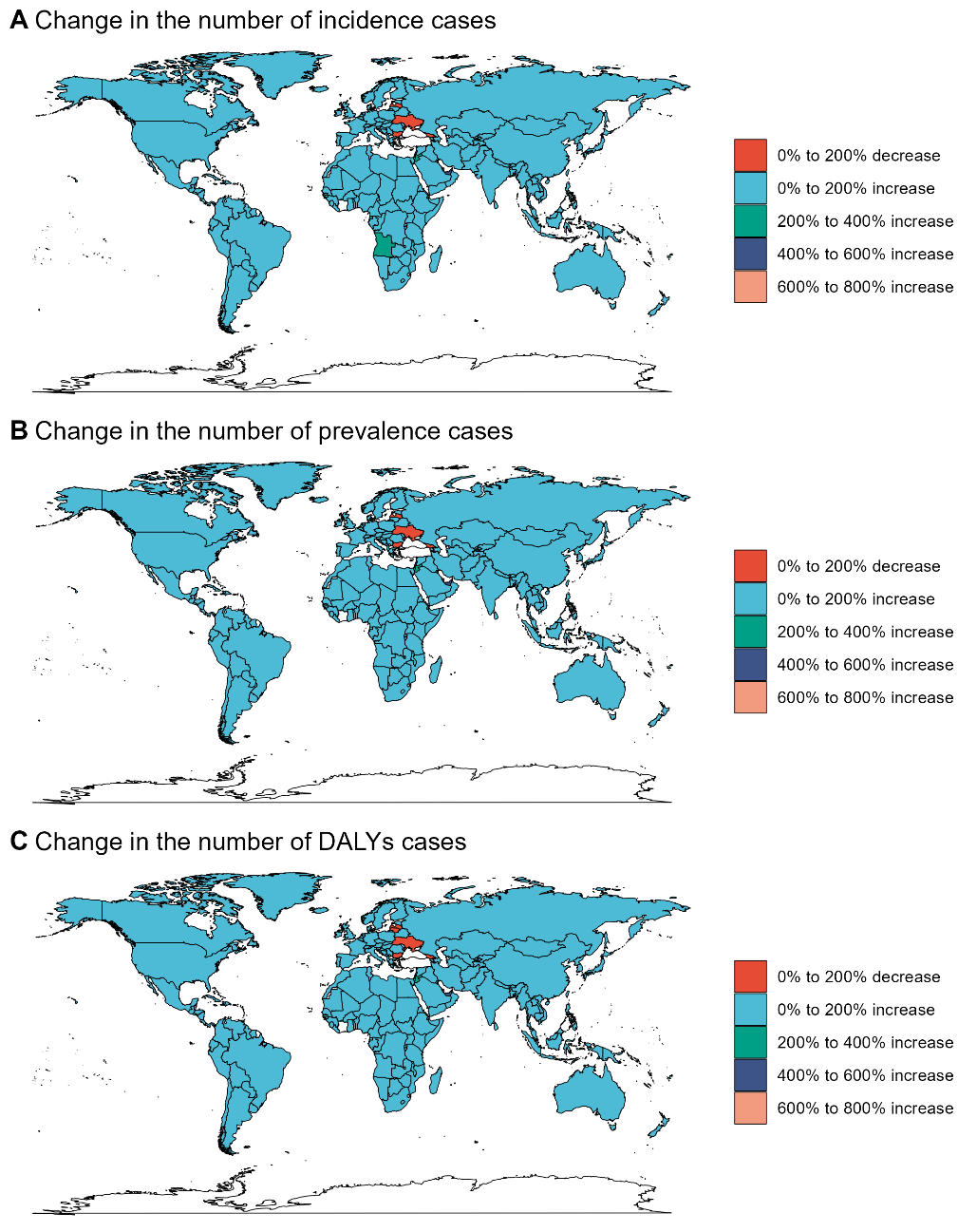
**

**Supplementary Figure S4** SDI-specific Incidence, Prevalence, and DALYs of fungal skin diseases in 2021. ASRs (A) and number of cases (B) across different SDI levels.


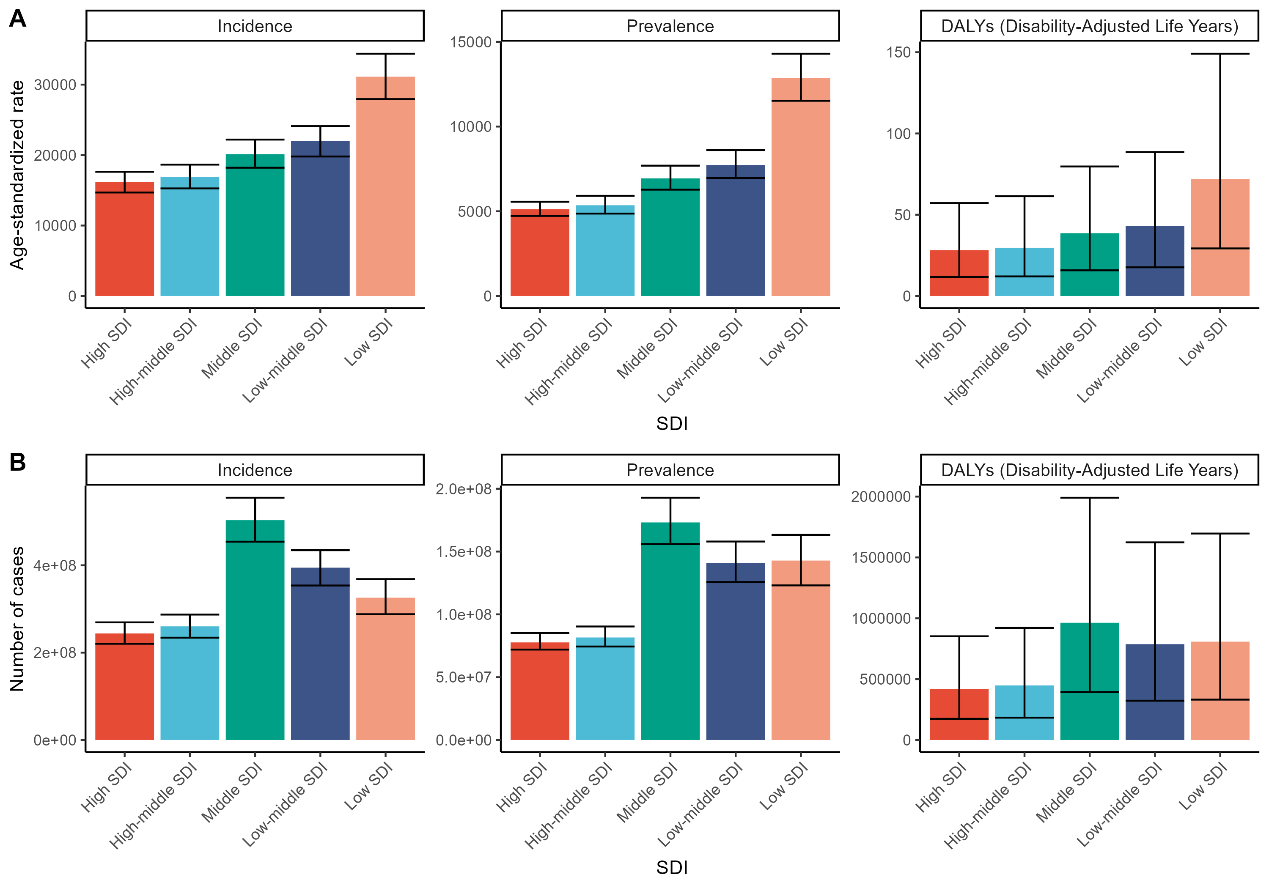


**Supplementary Figure S5** The global distribution of ASRs and and numbers of fungal skin diseases-related incidence, prevalence and DALYs in 2021.


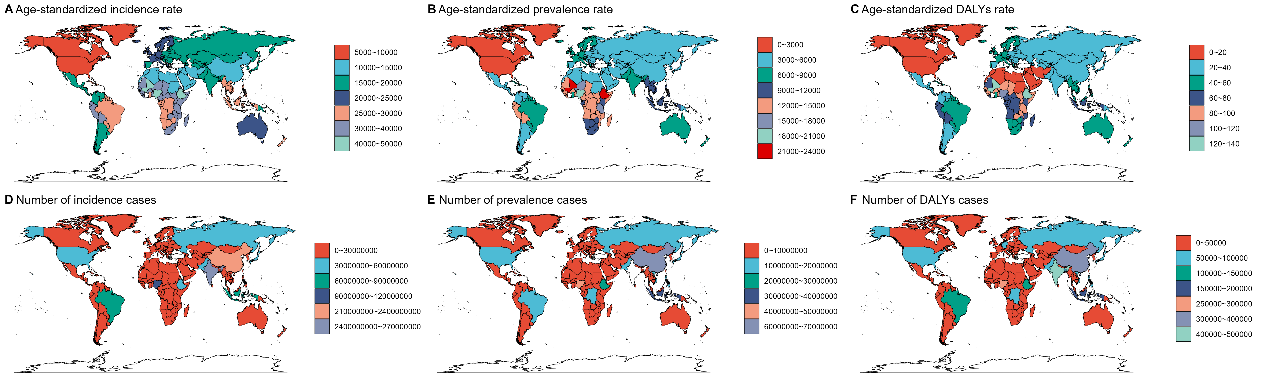


**Supplementary Figure S6** Global trends in numbers and age-standardized rates for (A) incidence, (B) prevalence, and (C) DALYs of fungal skin diseases from 1990 to 2021. Abbreviations: DALYs, disability-adjusted life years.


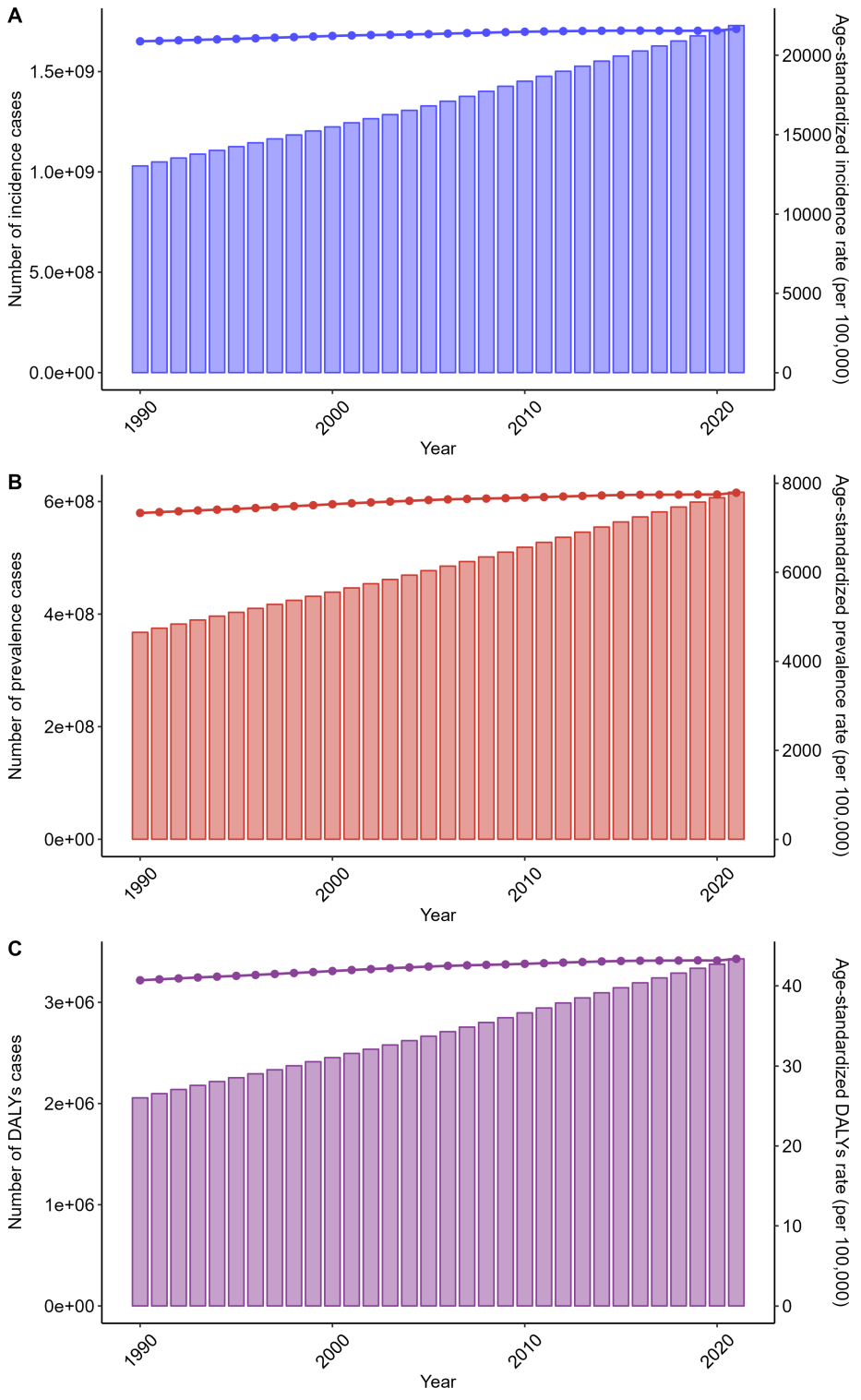


**Supplementary Figure S7** Global trends in (A) age-standardized rates and (B) numbers of incidence, prevalence, and DALYs of fungal skin diseases by age from 1990 to 2021.


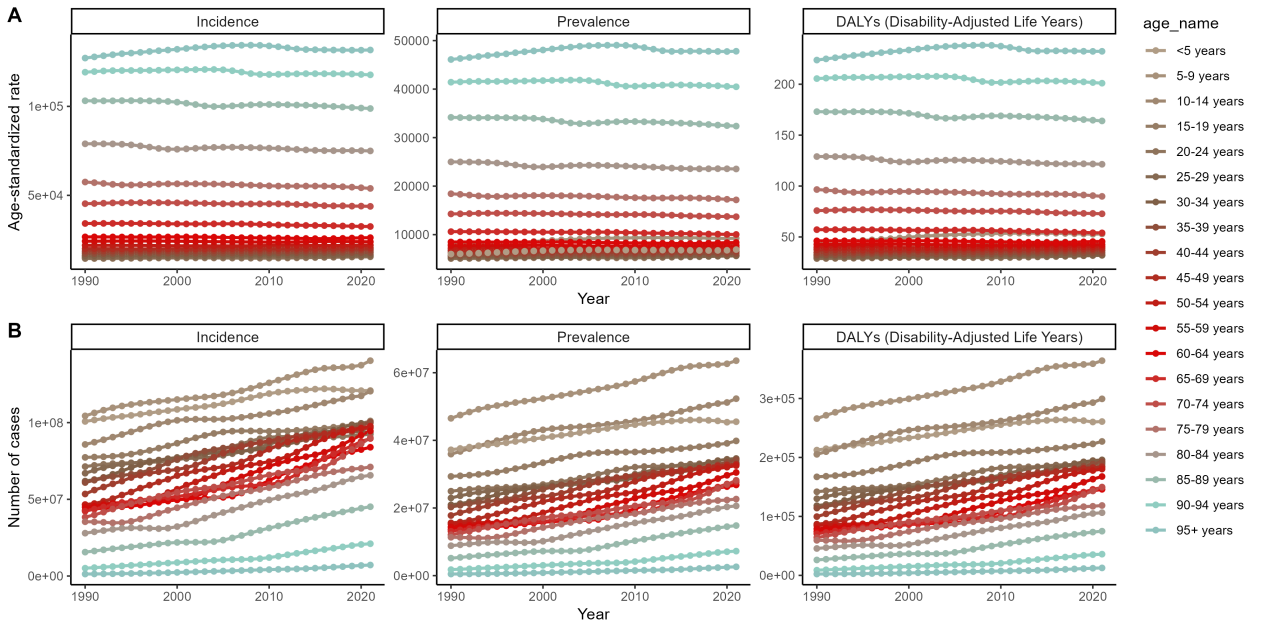


**Supplementary Figure S8** Global trends in (A) age-standardized rates and (B) numbers of incidence, prevalence, and DALYs of fungal skin diseases by gender from 1990 to 2021.


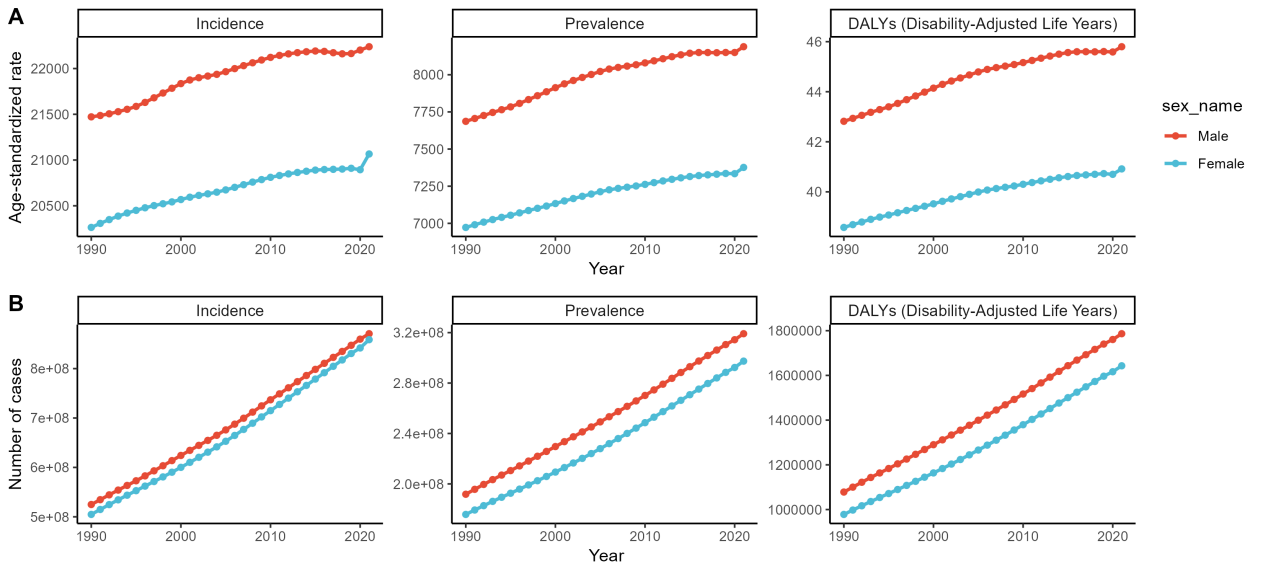


**Supplementary Figure S9** Global trends in (A) age-standardized rates and (B) numbers for incidence, prevalence, and DALYs of fungal skin diseases by SDI level from 1990 to 2021. Abbreviations: DALYs, disability-adjusted life years; SDI, socio-demographic index.


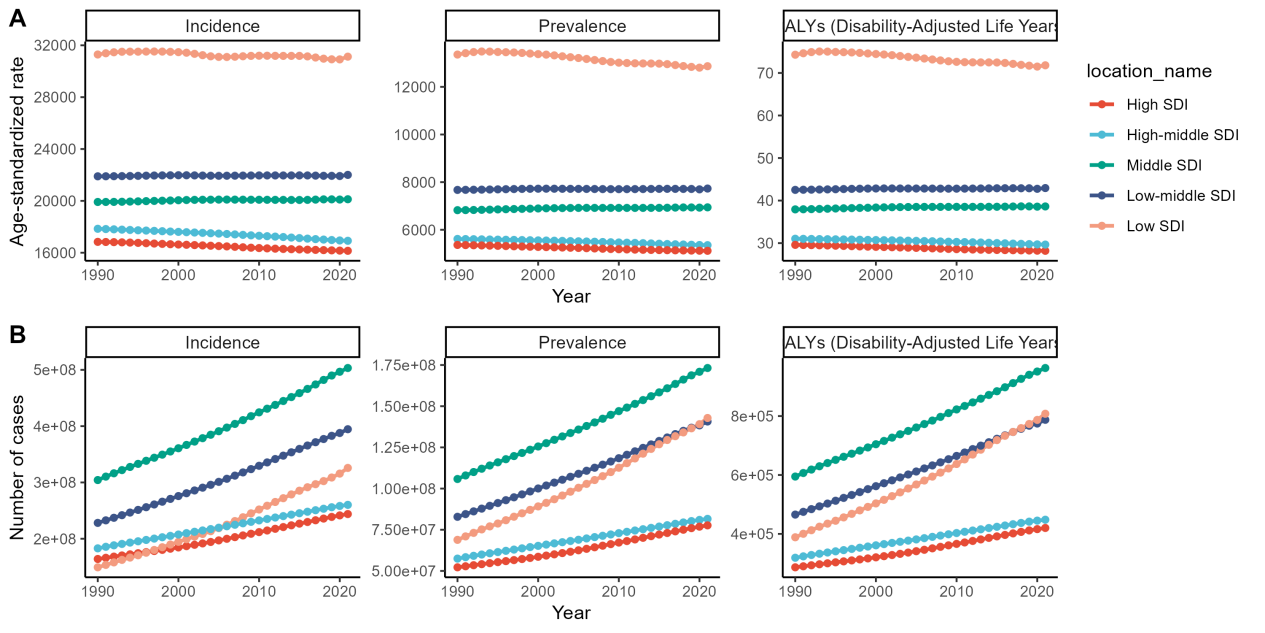


**Supplementary Tables**

**Supplementary Table S1** Geographic Disparities in ASIR, ASPR, and ASDR of fungal skin diseases in 2021 and temporary trends, 1990-2021.

|  | 1990 |  |  |  |  |  | 2021 |  |  |  |  |  | 1990-2021 |  |  |
| --- | --- | --- | --- | --- | --- | --- | --- | --- | --- | --- | --- | --- | --- | --- | --- |
|  | Incidence |  | Prevalence |  | DALYs |  | Incidence |  | Prevalence |  | DALYs |  | ASIR | ASPR | ASDR |
| Characteristics | Numbers (95% UI) | ASR | Numbers (95% UI) | ASR | Numbers (95% UI) | ASR | Numbers (95% UI) | ASR | Numbers (95% UI) | ASR | Numbers (95% UI) | ASR | (95% CI) | (95% CI) | (95% CI) |
|  |  | No. ×10-5 (95%UI) |  | No. ×10-5 (95%UI) |  | No. ×10-5 (95%UI) |  | No. ×10-5 (95%UI) |  | No. ×10-5 (95%UI) |  | No. ×10-5 (95%UI) | EAPC_CI | EAPC_CI | EAPC_CI |
| Global | 1029725109 (927052499-1132464184) | 20886.86 (18919.67-22868.75) | 367581342 (331514113-409319905) | 7334.21 (6652.51-8063.66) | 2056431 (842538-4248966) | 40.72 (16.77-83.84) | 1729224009 (1562709709-1894666053) | 21668.4 (19601.19-23729.17) | 616532287 (558724637-679274726) | 7789.55 (7059.28-8583.54) | 3429497 (1407894-7044259) | 43.39 (17.79-89.1) | 0.11 (0.1-0.12) | 0.19 (0.18-0.21) | 0.21 (0.19-0.22) |
| Sex |  |  |  |  |  |  |  |  |  |  |  |  |  |  |  |
| Female | 504827218 (455605510-554136942) | 20263.46 (18354.22-22181.69) | 175784255 (159284599-194590264) | 6972.54 (6327.69-7663.52) | 978161 (401084-2008760) | 38.58 (15.89-79.12) | 858570870 (775052838-940484924) | 21066.02 (19076.14-23048.66) | 297390154 (269989615-326373246) | 7376.39 (6691.79-8118.92) | 1642990 (675685-3361323) | 40.92 (16.78-83.77) | 0.11 (0.1-0.11) | 0.17 (0.16-0.18) | 0.19 (0.18-0.2) |
| Male | 524897891 (469857515-577845984) | 21472.06 (19440.71-23519.13) | 191797086 (171524373-214573712) | 7686.5 (6974.66-8470.74) | 1078270 (441454-2240206) | 42.82 (17.62-88.48) | 870653138 (786153592-954661669) | 22238.63 (20103.72-24325.69) | 319142132 (287775972-352445711) | 8188.82 (7401.08-9037.07) | 1786507 (732065-3683464) | 45.8 (18.77-94.26) | 0.12 (0.11-0.13) | 0.21 (0.19-0.22) | 0.22 (0.2-0.24) |
| Age |  |  |  |  |  |  |  |  |  |  |  |  |  |  |  |
| <5 years | 100776145 (78636317-132312846) | 16255.84 (12684.55-21342.92) | 37203602 (28212242-47153793) | 6001.18 (4550.82-7606.21) | 212453 (82231-455984) | 34.27 (13.26-73.55) | 120927681 (93928342-159813892) | 18373.27 (14271.1-24281.49) | 45508896 (34537845-57508564) | 6914.44 (5247.54-8737.62) | 260874 (101892-560076) | 39.64 (15.48-85.1) | 0.28 (0.2-0.36) | 0.34 (0.24-0.44) | 0.36 (0.26-0.45) |
| 5-9 years | 104386958 (76959419-135889840) | 17888.85 (13188.58-23287.52) | 46515657 (33515957-63353057) | 7971.42 (5743.65-10856.85) | 265871 (99198-596240) | 45.56 (17-102.18) | 140377507 (103902413-182566892) | 20431.81 (15122.9-26572.44) | 63559849 (46563538-87068326) | 9251.08 (6777.28-12672.71) | 364101 (136766-810147) | 52.99 (19.91-117.92) | 0.51 (0.43-0.58) | 0.57 (0.47-0.67) | 0.58 (0.48-0.68) |
| 10-14 years | 85767278 (65217780-107916317) | 16010.84 (12174.71-20145.57) | 35932752 (26185061-46654632) | 6707.84 (4888.17-8709.38) | 205268 (78019-461197) | 38.32 (14.56-86.1) | 120481012 (91065535-152565032) | 18072.98 (13660.46-22885.81) | 52318399 (37905098-68156101) | 7848.12 (5686.03-10223.89) | 299360 (114858-668601) | 44.91 (17.23-100.29) | 0.46 (0.41-0.51) | 0.59 (0.54-0.64) | 0.59 (0.54-0.65) |
| 15-19 years | 77349904 (57013870-101556991) | 14891.49 (10976.38-19551.87) | 29337542 (22173830-38763398) | 5648.1 (4268.93-7462.77) | 167089 (66981-349321) | 32.17 (12.9-67.25) | 101087950 (75073726-132786628) | 16200.51 (12031.43-21280.59) | 39837408 (30014548-52678389) | 6384.41 (4810.18-8442.32) | 227132 (90643-469541) | 36.4 (14.53-75.25) | 0.25 (0.21-0.29) | 0.35 (0.29-0.4) | 0.35 (0.3-0.41) |
| 20-24 years | 71341479 (57371181-87651561) | 14497.71 (11658.73-17812.18) | 24964261 (20157006-31016561) | 5073.13 (4096.22-6303.05) | 141769 (55456-299597) | 28.81 (11.27-60.88) | 92458948 (74386700-113736932) | 15483.16 (12456.78-19046.37) | 33545236 (27090404-41668246) | 5617.48 (4536.55-6977.76) | 190707 (74691-402710) | 31.94 (12.51-67.44) | 0.16 (0.13-0.19) | 0.25 (0.2-0.3) | 0.26 (0.21-0.31) |
| 25-29 years | 67012279 (50497994-85122463) | 15139.92 (11408.89-19231.51) | 22959632 (17660416-28701510) | 5187.21 (3989.98-6484.46) | 130060 (49862-282546) | 29.38 (11.27-63.84) | 93914049 (70852546-119215048) | 15962.46 (12042.72-20262.84) | 33334860 (25651243-41749019) | 5665.89 (4359.91-7096.03) | 188984 (72157-411583) | 32.12 (12.26-69.96) | 0.14 (0.11-0.16) | 0.24 (0.2-0.27) | 0.24 (0.21-0.27) |
| 30-34 years | 61870064 (47092322-78057587) | 16052.53 (12218.36-20252.47) | 20918470 (15836293-26477044) | 5427.41 (4108.81-6869.62) | 118197 (45426-254257) | 30.67 (11.79-65.97) | 99984977 (75920320-126487219) | 16540.65 (12559.6-20924.96) | 34635018 (26249464-43793312) | 5729.72 (4342.49-7244.79) | 195866 (74571-422005) | 32.4 (12.34-69.81) | 0.1 (0.09-0.12) | 0.19 (0.17-0.21) | 0.2 (0.18-0.22) |
| 35-39 years | 60935899 (51072200-71417818) | 17299.32 (14499.08-20275.07) | 20284961 (16973802-24363655) | 5758.77 (4818.76-6916.69) | 114159 (45067-235170) | 32.41 (12.79-66.76) | 100341674 (83821382-117831221) | 17890.49 (14944.99-21008.8) | 34239110 (28486112-41134237) | 6104.69 (5078.95-7334.06) | 192791 (76165-397311) | 34.37 (13.58-70.84) | 0.11 (0.1-0.12) | 0.19 (0.17-0.21) | 0.2 (0.18-0.22) |
| 40-44 years | 53477780 (44511245-62879476) | 18667.09 (15537.21-21948.87) | 17783109 (14717796-21313443) | 6207.42 (5137.43-7439.72) | 99637 (39469-206473) | 34.78 (13.78-72.07) | 96751021 (80620447-114140311) | 19340.5 (16116.01-22816.62) | 32995589 (27345610-39566406) | 6595.81 (5466.38-7909.31) | 184917 (73330-382237) | 36.96 (14.66-76.41) | 0.11 (0.09-0.12) | 0.18 (0.16-0.2) | 0.18 (0.17-0.2) |
| 45-49 years | 46564128 (36615488-57184437) | 20053.85 (15769.25-24627.71) | 15603103 (12176011-19372282) | 6719.81 (5243.86-8343.09) | 86938 (34022-177436) | 37.44 (14.65-76.42) | 96728845 (76290522-118927771) | 20428.28 (16111.89-25116.5) | 32927765 (25659402-41011286) | 6954.05 (5419.04-8661.22) | 183588 (72058-373556) | 38.77 (15.22-78.89) | 0.07 (0.06-0.09) | 0.13 (0.1-0.15) | 0.13 (0.11-0.16) |
| 50-54 years | 46563918 (36920937-56848248) | 21905.06 (17368.72-26743.12) | 15282223 (11918669-18643970) | 7189.22 (5606.9-8770.68) | 84617 (34075-172803) | 39.81 (16.03-81.29) | 97699790 (77555728-119298531) | 21958.81 (17431.27-26813.3) | 32540998 (25440448-39789715) | 7313.85 (5717.94-8943.06) | 180323 (72410-368720) | 40.53 (16.27-82.87) | 0 (-0.02-0.02) | 0.05 (0.03-0.08) | 0.06 (0.03-0.09) |
| 55-59 years | 44899018 (36186259-54575230) | 24243.54 (19539.02-29468.27) | 14373224 (11850614-17640628) | 7760.92 (6398.82-9525.18) | 78980 (30989-170656) | 42.65 (16.73-92.15) | 94532641 (76257337-115156382) | 23888.29 (19270.14-29099.88) | 30502018 (25218636-37375840) | 7707.82 (6372.72-9444.83) | 167668 (66271-360694) | 42.37 (16.75-91.15) | -0.06 (-0.09--0.04) | -0.04 (-0.07--0.01) | -0.03 (-0.06-0) |
| 60-64 years | 42812596 (31202604-57603281) | 26656.37 (19427.65-35865.48) | 13628317 (10388319-18047661) | 8485.39 (6468.07-11237) | 74189 (28890-162487) | 46.19 (17.99-101.17) | 83953524 (61257555-113027393) | 26231.55 (19140.12-35315.78) | 26822786 (20447217-35433145) | 8380.87 (6388.8-11071.2) | 145977 (57221-319015) | 45.61 (17.88-99.68) | -0.12 (-0.15--0.1) | -0.12 (-0.15--0.09) | -0.11 (-0.14--0.09) |
| 65-69 years | 42272684 (33770573-52717536) | 34198.58 (27320.38-42648.47) | 13121756 (10500695-16394301) | 10615.49 (8495.06-13262.98) | 70665 (28219-152841) | 57.17 (22.83-123.65) | 89646919 (71312792-112216509) | 32499.35 (25852.75-40681.42) | 27683322 (22059955-34485085) | 10035.93 (7997.31-12501.75) | 148996 (59282-320893) | 54.01 (21.49-116.33) | -0.17 (-0.19--0.15) | -0.18 (-0.2--0.16) | -0.18 (-0.2--0.15) |
| 70-74 years | 38375668 (30921017-48193197) | 45328.54 (36523.26-56924.81) | 12078651 (9798073-14745987) | 14267.05 (11573.28-17417.65) | 64233 (26853-137015) | 75.87 (31.72-161.84) | 90184664 (72564997-113044246) | 43813.16 (35253.24-54918.71) | 28182763 (22857973-34500040) | 13691.64 (11104.77-16760.67) | 149772 (62528-319527) | 72.76 (30.38-155.23) | -0.14 (-0.16--0.11) | -0.16 (-0.18--0.13) | -0.15 (-0.18--0.13) |
| 75-79 years | 35392854 (25687153-47034044) | 57497.45 (41730.06-76409.15) | 11342502 (8526500-14494799) | 18426.46 (13851.72-23547.52) | 59448 (23514-127238) | 96.58 (38.2-206.7) | 71082869 (51322788-94373594) | 53897.86 (38914.98-71557.81) | 22635198 (16973048-28967187) | 17162.91 (12869.64-21964.07) | 118523 (46819-252032) | 89.87 (35.5-191.1) | -0.14 (-0.16--0.11) | -0.16 (-0.19--0.14) | -0.16 (-0.19--0.14) |
| 80-84 years | 27937144 (22767708-34471354) | 78972.19 (64359.33-97442.97) | 8842446 (7276317-10742333) | 24995.66 (20568.55-30366.22) | 45620 (18700-94361) | 128.96 (52.86-266.74) | 65662246 (53382313-81056011) | 74971.4 (60950.5-92547.59) | 20615072 (16918110-25085794) | 23537.74 (19316.65-28642.29) | 106328 (43539-220759) | 121.4 (49.71-252.06) | -0.16 (-0.19--0.13) | -0.19 (-0.22--0.16) | -0.19 (-0.22--0.16) |
| 85-89 years | 15586901 (12839856-18784244) | 103148.87 (84969.85-124307.82) | 5164640 (4402572-6043038) | 34177.85 (29134.74-39990.8) | 26154 (10410-53519) | 173.08 (68.89-354.17) | 45168779 (37206923-54571146) | 98790.48 (81376.78-119354.79) | 14801119 (12582441-17474459) | 32372.13 (27519.57-38219.1) | 74965 (29776-153156) | 163.96 (65.12-334.97) | -0.13 (-0.16--0.11) | -0.17 (-0.19--0.14) | -0.16 (-0.19--0.14) |
| 90-94 years | 5107940 (4063286-6209792) | 119199.92 (94821.66-144912.96) | 1775127 (1474750-2078627) | 41424.72 (34415.07-48507.26) | 8808 (3440-18494) | 205.54 (80.27-431.57) | 21061857 (16666450-25701571) | 117733.9 (93163.97-143669.48) | 7241521 (5981188-8548914) | 40479.46 (33434.31-47787.67) | 35962 (14089-76005) | 201.02 (78.75-424.86) | -0.07 (-0.1--0.05) | -0.1 (-0.13--0.07) | -0.1 (-0.12--0.07) |
| 95+ years | 1294472 (990418-1620389) | 127147.57 (97282.35-159160.26) | 469367 (379330-559701) | 46102.88 (37259.14-54975.76) | 2278 (894-4848) | 223.72 (87.85-476.22) | 7177054 (5463961-9066458) | 131681.61 (100250.5-166347.61) | 2605359 (2077390-3130430) | 47802.04 (38115.09-57435.82) | 12662 (4953-27132) | 232.32 (90.88-497.8) | 0.09 (0.04-0.13) | 0.09 (0.03-0.15) | 0.09 (0.04-0.15) |
| SDI region |  |  |  |  |  |  |  |  |  |  |  |  |  |  |  |
| High-middle SDI | 182944942 (164586505-202660631) | 17846.79 (16107.86-19680.59) | 57487860 (52138040-63358073) | 5615.42 (5120.44-6196.25) | 319040 (129267-667097) | 31.03 (12.64-64.11) | 260188514 (234220160-287174277) | 16920.08 (15266.5-18644.11) | 81602182 (74395167-90346743) | 5354.46 (4863.19-5911.78) | 448114 (183348-921094) | 29.66 (12.03-61.42) | -0.17 (-0.18--0.17) | -0.16 (-0.16--0.15) | -0.15 (-0.15--0.14) |
| High SDI | 164047851 (148679463-180005645) | 16838.08 (15269.79-18422.36) | 52201565 (48117591-56853285) | 5363.56 (4940.22-5831.08) | 286599 (118648-583420) | 29.59 (12.17-60.11) | 243866476 (220333079-269297424) | 16147.92 (14687.3-17628.18) | 77663743 (71961405-85232930) | 5117.27 (4725.72-5561.42) | 420160 (173757-852117) | 28.19 (11.64-57.15) | -0.15 (-0.15--0.14) | -0.16 (-0.17--0.16) | -0.17 (-0.17--0.16) |
| Low-middle SDI | 228159740 (201573548-256016979) | 21894.64 (19644.48-24042.14) | 82858840 (72439463-95482103) | 7673.41 (6884.28-8556.43) | 465575 (190126-970238) | 42.51 (17.51-88.15) | 394534381 (353702982-434592656) | 22004.64 (19797.44-24106.7) | 140711272 (125727190-157977912) | 7731.22 (6958.71-8612.32) | 787326 (322644-1623830) | 42.94 (17.65-88.57) | 0.01 (0-0.01) | 0.01 (0.01-0.02) | 0.02 (0.02-0.03) |
| Low SDI | 149258374 (131098860-169929148) | 31292.33 (28086.59-34534.55) | 68866091 (59045356-79465444) | 13356.5 (11915.92-14866.85) | 388542 (157546-815826) | 74.29 (30.31-155.18) | 325796367 (287974642-368388493) | 31124.75 (27943.94-34383.12) | 142839638 (123058640-163253386) | 12866.92 (11521.34-14298.53) | 807825 (331736-1696213) | 71.8 (29.26-149.06) | -0.05 (-0.07--0.04) | -0.18 (-0.19--0.16) | -0.16 (-0.18--0.15) |
| Middle SDI | 304257184 (273157584-336410055) | 19915.79 (18062.59-21958.41) | 105809185 (94792497-118247916) | 6823.62 (6184.91-7537.79) | 594688 (241708-1243206) | 37.93 (15.56-78.24) | 503290634 (454024197-554675359) | 20125.73 (18206.98-22198.06) | 173183772 (155963056-192835881) | 6941.77 (6276.73-7692.35) | 963136 (393633-1990445) | 38.62 (15.78-79.68) | 0.03 (0.02-0.04) | 0.05 (0.04-0.06) | 0.06 (0.05-0.06) |
| GBD region |  |  |  |  |  |  |  |  |  |  |  |  |  |  |  |
| Advanced Health System | 253964195 (228647912-280140608) | 17519.98 (15869.01-19234.69) | 80153409 (73700531-87910350) | 5540.87 (5095.02-6048.94) | 439636 (181013-897939) | 30.51 (12.49-62.17) | 353429209 (317731006-393573829) | 16871.15 (15305.91-18454) | 111952117 (103319306-123477198) | 5328.79 (4910.06-5821.1) | 605603 (250558-1231249) | 29.32 (12.04-59.54) | -0.13 (-0.13--0.13) | -0.13 (-0.14--0.13) | -0.13 (-0.14--0.13) |
| Africa | 186035101 (164163667-211020242) | 31770.59 (28519.03-35020.3) | 85336609 (73397712-98151384) | 13594.95 (12183.05-15099.78) | 481476 (196287-1008579) | 75.63 (30.87-157.89) | 415588373 (367279665-468988912) | 32283.04 (28891.22-35760.18) | 182826199 (158551974-209010397) | 13490.83 (12084.67-15003.31) | 1033418 (424470-2167081) | 75.28 (30.72-156.29) | 0.03 (0.01-0.04) | -0.06 (-0.08--0.04) | -0.05 (-0.07--0.02) |
| African Region | 173281615 (152749999-197278986) | 36332.7 (32587.59-40175.64) | 80968569 (69476903-93258571) | 15863.57 (14169.93-17623.41) | 456954 (185946-959229) | 88.27 (36.04-184.42) | 389458344 (344207067-440511044) | 36251.41 (32418.39-40191.83) | 174015296 (150512947-198902524) | 15381.74 (13766.71-17116.51) | 984190 (404385-2069953) | 85.83 (35.03-178.5) | -0.04 (-0.05--0.02) | -0.14 (-0.16--0.12) | -0.13 (-0.15--0.11) |
| America | 111352404 (101299772-121896587) | 16252.03 (14837.86-17759.74) | 35757823 (32853008-39016867) | 5203.27 (4792.66-5663.03) | 198498 (82102-404728) | 28.77 (11.94-58.48) | 196643941 (179411031-214791262) | 17401.43 (15896.42-18990.99) | 62835591 (58002628-68274539) | 5581.8 (5142.02-6092.28) | 343841 (141839-702315) | 30.76 (12.71-62.56) | 0.19 (0.18-0.2) | 0.2 (0.19-0.21) | 0.19 (0.18-0.21) |
| Andean Latin America | 10193849 (9064771-11351240) | 31413.72 (28239.91-35011.48) | 4019757 (3548188-4511321) | 12382.33 (11123.17-13682.78) | 22541 (9203-46264) | 68.66 (28.3-141.8) | 19833548 (17793929-22026926) | 30930.16 (27772.91-34537.99) | 7803891 (6972788-8667501) | 12185.3 (10928.53-13484.34) | 43352 (17759-89416) | 67.51 (27.8-139.33) | -0.05 (-0.05--0.05) | -0.05 (-0.06--0.05) | -0.05 (-0.05--0.05) |
| Asia | 548718354 (493019864-607158786) | 19161.63 (17353.71-21088.7) | 188142840 (167610627-212216316) | 6462.94 (5830.75-7150.24) | 1056881 (431891-2191640) | 35.94 (14.79-73.93) | 883292233 (796351242-972024863) | 18796.41 (17031.24-20680.66) | 296194797 (266859176-329067591) | 6326.96 (5714.7-7013.89) | 1647941 (675987-3379970) | 35.22 (14.47-72.12) | -0.06 (-0.08--0.05) | -0.07 (-0.09--0.05) | -0.07 (-0.09--0.05) |
| Australasia | 5341166 (4857132-5850349) | 24673.75 (22362.41-26952.61) | 1894644 (1807376-1988591) | 8724.92 (8310.84-9178.82) | 10378 (4273-21221) | 47.89 (19.72-98.27) | 9670089 (8787276-10688314) | 24492.01 (22211.11-26796.32) | 3551173 (3401679-3705444) | 8674.33 (8253.11-9133.01) | 19229 (7927-38968) | 47.67 (19.51-98.06) | -0.02 (-0.03--0.02) | -0.02 (-0.02--0.02) | -0.02 (-0.02--0.02) |
| Basic Health System | 382063585 (343058096-423792940) | 18591.14 (16842.91-20513.06) | 129265865 (115874306-144373885) | 6222.86 (5637.23-6898) | 726290 (294292-1521595) | 34.61 (14.15-71.68) | 610883092 (552456922-675697980) | 18432.92 (16656.83-20388.47) | 205269521 (185333642-228358247) | 6220.5 (5599.22-6913.16) | 1140413 (463780-2366474) | 34.62 (14.07-71.9) | -0.03 (-0.04--0.03) | -0.01 (-0.01-0) | 0 (-0.01-0) |
| Caribbean | 8207389 (7360613-9123056) | 25752.81 (23051.35-28670.64) | 2711422 (2427919-3035556) | 8515.74 (7718.67-9449.95) | 15100 (6252-31403) | 47.12 (19.55-97.27) | 12791954 (11443688-14243213) | 25655.03 (22966.31-28573.75) | 4231139 (3838371-4683834) | 8490.95 (7688.73-9420.81) | 23271 (9624-48117) | 46.84 (19.43-97.05) | -0.01 (-0.01--0.01) | -0.01 (-0.01-0) | -0.01 (-0.02--0.01) |
| Central Africa | 20484445 (17463872-24175698) | 32291.57 (28841.13-36061.16) | 10048622 (7976811-12496632) | 13935.02 (11856.57-16185.5) | 56879 (22595-122175) | 77.68 (31.94-164.57) | 48237798 (41482488-55849219) | 31538.96 (28092.31-34996.99) | 21276670 (17521188-25389145) | 12595.3 (10959.73-14327.81) | 120675 (49093-256512) | 70.34 (28.8-146.23) | -0.08 (-0.11--0.05) | -0.34 (-0.36--0.32) | -0.33 (-0.35--0.31) |
| Central Asia | 10559734 (9428543-11751805) | 18055.7 (16126.69-19996.84) | 3231788 (2917597-3617286) | 5504.35 (4997.22-6131.34) | 18002 (7279-37231) | 30.38 (12.36-62.24) | 15899318 (14300610-17629393) | 18048.07 (16114.29-19997.58) | 4840575 (4368567-5413076) | 5502.46 (4989.41-6130.45) | 26874 (10846-55460) | 30.35 (12.34-61.99) | 0 (0-0) | 0 (0-0) | 0 (0-0) |
| Central Europe | 24176793 (21543318-26849565) | 18162.47 (16240.02-20130.2) | 7355844 (6671492-8184611) | 5550.36 (5049.3-6168.33) | 40299 (16332-82393) | 30.45 (12.38-62.35) | 28988989 (25732360-32200694) | 18152.72 (16232.24-20130.03) | 8797948 (8015062-9865405) | 5547.72 (5046.84-6164.82) | 47583 (19403-96013) | 30.52 (12.41-62.55) | 0 (0-0) | 0 (0-0) | 0.01 (0.01-0.01) |
| Central Latin America | 24222698 (21749112-26844546) | 18047.92 (16335.79-19955.61) | 7391596 (6689263-8241211) | 5414.07 (4938.36-5985.35) | 41411 (17044-85072) | 29.84 (12.32-61.24) | 47243257 (42479108-52194135) | 18823.54 (16934.01-20811.19) | 14252467 (12956015-15754044) | 5684.18 (5172.88-6293.48) | 78631 (32252-162385) | 31.33 (12.92-64.32) | 0.08 (0.02-0.15) | 0.1 (0.02-0.17) | 0.1 (0.02-0.17) |
| Central Sub-Saharan Africa | 16346538 (13835155-19683382) | 30749.75 (27222.42-34817.98) | 8604108 (6617077-10892031) | 13775.24 (11384.47-16448.73) | 48803 (19618-105727) | 76.96 (30.93-165.24) | 37889970 (32068840-44945141) | 29742.7 (26265.95-33437.93) | 17734503 (13834922-21856841) | 12157.24 (10215.64-14241.13) | 100816 (41085-218359) | 68.05 (27.91-145.3) | -0.12 (-0.17--0.08) | -0.44 (-0.47--0.4) | -0.42 (-0.45--0.39) |
| Commonwealth High Income | 25973271 (23348168-28594386) | 20500.02 (18483.69-22504.2) | 8541990 (7865473-9333567) | 6728.85 (6178.26-7356.18) | 46837 (19376-95533) | 37.14 (15.25-75.58) | 38459429 (34594538-42656520) | 19992.41 (18045.64-21926.46) | 12803358 (11837519-13953009) | 6578.44 (6057.03-7160.71) | 69489 (28727-142333) | 36.28 (14.89-73.76) | -0.08 (-0.09--0.07) | -0.07 (-0.08--0.06) | -0.07 (-0.08--0.07) |
| Commonwealth Low Income | 51907560 (45803967-59054513) | 26998.29 (24292.13-29775.35) | 20840867 (18081290-23901227) | 10117.04 (9168.96-11254.93) | 117548 (47852-247842) | 56.22 (23.19-116.91) | 102595278 (91002288-114451356) | 27994.6 (25136.23-31102.84) | 39915690 (35693883-44786769) | 10472.17 (9502-11613.22) | 225200 (92360-466267) | 58.51 (24.14-121.54) | 0.09 (0.08-0.09) | 0.08 (0.07-0.09) | 0.1 (0.09-0.11) |
| Commonwealth Middle Income | 250337096 (219386513-282219678) | 23169.23 (20701.31-25586.32) | 92602863 (80223768-107254744) | 8284.27 (7380.16-9276.12) | 520018 (212075-1093344) | 45.88 (18.85-95.68) | 463152927 (411852656-514147847) | 23380.28 (20908.73-25757.91) | 169345970 (150793191-191484428) | 8405.07 (7504.47-9383.54) | 947956 (387037-1955704) | 46.71 (19.14-96.49) | 0.01 (0-0.02) | 0.01 (0-0.02) | 0.03 (0.02-0.04) |
| East Asia | 167616611 (149979157-188222894) | 14788.08 (13376.34-16446.37) | 51925558 (46257048-58221090) | 4557.19 (4102.12-5060.04) | 292491 (117538-612081) | 25.48 (10.34-53.03) | 236950733 (212978978-263127313) | 14227.76 (12819.42-15818.22) | 71809813 (64761791-79801046) | 4346.89 (3907.34-4830.3) | 398723 (162212-830635) | 24.28 (9.83-50.71) | -0.15 (-0.15--0.14) | -0.18 (-0.19--0.17) | -0.18 (-0.19--0.17) |
| East Asia & Pacific - WB | 320188362 (287924587-355624138) | 18638.59 (16854.54-20603.79) | 108954080 (97690928-121864882) | 6286.27 (5680.34-6971.45) | 612420 (246629-1285792) | 35.05 (14.26-72.77) | 493471026 (445337081-545418181) | 18931.12 (17070.06-20998.76) | 168020458 (151829765-186218935) | 6512.54 (5843.85-7244.6) | 933927 (381021-1937494) | 36.39 (14.73-76.03) | 0.05 (0.04-0.05) | 0.11 (0.11-0.11) | 0.12 (0.12-0.12) |
| Eastern Africa | 61848278 (54213315-70853582) | 36592.14 (32953.52-40461.82) | 30588656 (26270914-35313409) | 16462.79 (14747.08-18388.7) | 173024 (70204-356155) | 91.79 (37.49-191.9) | 129640423 (114292406-147488845) | 36010.76 (32265.19-39926.47) | 58500238 (50607427-67131669) | 15314.95 (13701.77-17110.79) | 331376 (137057-688864) | 85.54 (34.91-178.53) | -0.1 (-0.11--0.08) | -0.3 (-0.32--0.28) | -0.29 (-0.31--0.27) |
| Eastern Europe | 44842701 (39923794-49893695) | 18381.18 (16428.66-20413.89) | 13694517 (12470642-15321528) | 5641.7 (5135.64-6281.9) | 74931 (30524-154499) | 30.95 (12.68-63.87) | 48985877 (43277150-54790606) | 18374.98 (16424.36-20390.18) | 14925815 (13583984-16793845) | 5640.64 (5132.74-6279.89) | 80814 (32830-163988) | 30.95 (12.69-63.75) | 0 (0-0) | 0 (0-0) | 0.01 (0-0.01) |
| Eastern Mediterranean Region | 44373046 (39771881-49118327) | 14522.72 (13075.66-15930.73) | 14684746 (13002397-16657355) | 4613.8 (4189.6-5106.6) | 82399 (33660-171401) | 25.44 (10.45-52.29) | 94469063 (85040706-104595716) | 14613.4 (13124.67-16014.21) | 30741564 (27480906-34414045) | 4655.7 (4203.4-5144.49) | 171726 (70426-350589) | 25.65 (10.54-52.27) | 0.03 (0.02-0.03) | 0.05 (0.04-0.05) | 0.05 (0.04-0.06) |
| Eastern Sub-Saharan Africa | 72346166 (63464615-82824862) | 39374.24 (35470.34-43535.9) | 35462459 (30335305-41008915) | 17634.06 (15740.23-19731.67) | 200529 (81601-414732) | 98.25 (40.12-205.62) | 153079011 (134806215-174216560) | 38643.84 (34630.18-42795.3) | 68902444 (59550670-78967916) | 16407.63 (14685.94-18312.69) | 390277 (162032-811951) | 91.58 (37.52-191.62) | -0.11 (-0.12--0.1) | -0.3 (-0.32--0.28) | -0.29 (-0.31--0.27) |
| Europe | 181560798 (162595087-201383470) | 20261.3 (18239.49-22351.84) | 57657464 (52575690-63919993) | 6452.33 (5889.44-7118.96) | 315766 (129757-649506) | 35.51 (14.5-72.85) | 230850181 (205196273-258386339) | 19975.45 (17981.87-22102.72) | 73711037 (67481642-82311739) | 6363.74 (5804.76-7026.69) | 399022 (164140-818404) | 35.04 (14.3-71.82) | -0.04 (-0.05--0.04) | -0.04 (-0.05--0.04) | -0.04 (-0.04--0.04) |
| Europe & Central Asia - WB | 188674070 (168963780-209313255) | 20147.43 (18143.67-22237.17) | 59835186 (54564355-66322777) | 6405.94 (5848.03-7071.44) | 327911 (134559-674606) | 35.25 (14.38-72.33) | 242299192 (215523650-270746409) | 19822.29 (17854.64-21929.36) | 77196046 (70720682-86177453) | 6301.17 (5750-6962.09) | 418431 (172155-858190) | 34.69 (14.14-71.11) | -0.05 (-0.05--0.05) | -0.05 (-0.05--0.05) | -0.05 (-0.05--0.04) |
| European Region | 190535228 (170649381-211334981) | 20196.49 (18194.2-22287.34) | 60575773 (55265949-67116876) | 6437.62 (5880.14-7102.88) | 332028 (136266-682777) | 35.43 (14.46-72.67) | 245929658 (218922077-274768878) | 19917.44 (17945.47-22027.66) | 78673462 (72137167-87728232) | 6360.73 (5810.46-7021.26) | 426584 (175561-875234) | 35.02 (14.28-71.74) | -0.04 (-0.04--0.04) | -0.04 (-0.04--0.03) | -0.03 (-0.03--0.03) |
| High-income Asia Pacific | 35797316 (32020269-39850237) | 19469.55 (17465.77-21702.23) | 11009300 (9923631-12218322) | 6018.41 (5452.11-6661.85) | 60872 (24589-122856) | 33.3 (13.51-66.95) | 58497384 (51388140-65973659) | 19223.06 (17249.74-21450.51) | 18137114 (16398804-20304408) | 5921.2 (5359.76-6562.53) | 97977 (40319-197988) | 32.82 (13.36-66.18) | -0.04 (-0.04--0.04) | -0.05 (-0.06--0.05) | -0.05 (-0.05--0.04) |
| High-income North America | 26165822 (24222434-28038044) | 8333.2 (7752.43-8915.04) | 7876402 (7536407-8235000) | 2507.13 (2396.61-2619.93) | 42997 (17645-88374) | 13.77 (5.63-28.41) | 41256177 (38055027-44314078) | 8438.69 (7866.31-8989.68) | 12239743 (11750935-12805812) | 2506.94 (2407.04-2606.08) | 65545 (27199-132567) | 13.67 (5.6-28.02) | -0.01 (-0.03-0) | -0.04 (-0.05--0.03) | -0.06 (-0.08--0.05) |
| Latin America & Caribbean - WB | 86138997 (77320411-95231100) | 22994.91 (20809.61-25300.36) | 28194528 (25490638-31203376) | 7488.87 (6840.03-8242.48) | 157235 (64495-322608) | 41.26 (17.05-84.49) | 156621682 (141642605-173260640) | 23141.86 (20926.19-25524.86) | 51007206 (46446377-56263869) | 7553.54 (6887.53-8322.43) | 280518 (115087-577090) | 41.59 (17.1-85.11) | 0 (-0.02-0.02) | 0.01 (-0.02-0.03) | 0.01 (-0.02-0.03) |
| Limited Health System | 350006806 (308232031-394830127) | 24110.16 (21559.96-26636.06) | 137459585 (118833319-159255544) | 9002.42 (8025.68-10095.51) | 773618 (314968-1619269) | 49.96 (20.5-104.18) | 662942299 (590589675-736730233) | 24526.83 (21949.74-27105.66) | 253334551 (224242130-286796151) | 9141.19 (8188.98-10208.07) | 1423128 (580522-2945609) | 50.94 (20.85-105.46) | 0.03 (0.02-0.04) | 0.01 (0-0.02) | 0.02 (0.01-0.04) |
| Middle East & North Africa - WB | 22656770 (20592316-24832961) | 12061.58 (10956.91-13236.21) | 7170045 (6579953-7853760) | 3759.79 (3460.96-4129.2) | 40097 (16557-81805) | 20.64 (8.49-41.76) | 47742873 (43130125-52324209) | 11647.33 (10549.03-12764.64) | 14690177 (13427873-16133919) | 3585.2 (3293.89-3929.44) | 81220 (33313-165250) | 19.59 (8.04-39.64) | -0.1 (-0.11--0.1) | -0.13 (-0.14--0.13) | -0.14 (-0.15--0.14) |
| Minimal Health System | 42633506 (36965093-49288413) | 33391.07 (29907.56-37099.61) | 20344680 (16952345-23981722) | 14510.61 (12789.37-16273.14) | 114899 (46556-242620) | 80.76 (33.19-167.65) | 100421774 (87459312-114939037) | 32795.73 (29408.89-36303.2) | 45444419 (38474617-52807053) | 13667.05 (12179.25-15155.14) | 257418 (105868-543900) | 76.18 (31.27-157.99) | -0.06 (-0.08--0.04) | -0.2 (-0.21--0.19) | -0.19 (-0.2--0.18) |
| North Africa and Middle East | 28816109 (26111450-31712059) | 11487.49 (10402-12614.06) | 8842810 (8057742-9701477) | 3458.53 (3165.88-3824.69) | 49414 (20248-101320) | 18.98 (7.75-38.49) | 59785973 (53911475-65710812) | 11122.47 (10049.39-12248.76) | 17854562 (16208416-19697750) | 3311.44 (3029.03-3657.63) | 98647 (40348-201213) | 18.09 (7.39-36.68) | -0.09 (-0.1--0.09) | -0.12 (-0.13--0.11) | -0.13 (-0.14--0.12) |
| North America | 26176456 (24232239-28049249) | 8336.51 (7755.36-8918.75) | 7880029 (7539795-8238792) | 2508.27 (2397.64-2621.18) | 43017 (17653-88416) | 13.78 (5.64-28.42) | 41272859 (38069638-44333222) | 8441.56 (7869.09-8992.78) | 12245520 (11756320-12811757) | 2507.94 (2407.92-2607.12) | 65576 (27212-132629) | 13.68 (5.61-28.03) | -0.01 (-0.03-0) | -0.04 (-0.05--0.03) | -0.06 (-0.08--0.05) |
| Northern Africa | 11078304 (10079708-12132724) | 12046.52 (10899.37-13221.25) | 3546806 (3237857-3879707) | 3734.68 (3434.66-4123.39) | 19877 (8184-40483) | 20.57 (8.49-41.6) | 20890925 (18905156-22869209) | 11603.82 (10507.31-12722.4) | 6376876 (5790797-7010471) | 3518.44 (3226.69-3889.04) | 35333 (14577-71959) | 19.26 (7.91-38.96) | -0.08 (-0.1--0.07) | -0.12 (-0.15--0.1) | -0.14 (-0.16--0.11) |
| Oceania | 1053505 (935422-1178099) | 19096.58 (17161.94-21205.07) | 335343 (297006-378225) | 5974.59 (5337.86-6667.67) | 1887 (769-3929) | 33.15 (13.54-68.16) | 2329919 (2081626-2608322) | 19063.29 (17141.24-21170.12) | 739478 (652407-832720) | 5973.01 (5353.77-6665.95) | 4155 (1687-8565) | 33.16 (13.45-68.35) | 0 (0-0) | 0 (0-0.01) | 0.01 (0.01-0.01) |
| Region of the Americas | 111352404 (101299772-121896587) | 16252.03 (14837.86-17759.74) | 35757823 (32853008-39016867) | 5203.27 (4792.66-5663.03) | 198498 (82102-404728) | 28.77 (11.94-58.48) | 196643941 (179411031-214791262) | 17401.43 (15896.42-18990.99) | 62835591 (58002628-68274539) | 5581.8 (5142.02-6092.28) | 343841 (141839-702315) | 30.76 (12.71-62.56) | 0.19 (0.18-0.2) | 0.2 (0.19-0.21) | 0.19 (0.18-0.21) |
| South-East Asia Region | 261981090 (231038248-292206376) | 22124.96 (19788.85-24348.08) | 95194647 (83253844-109631568) | 7801.05 (6974.72-8690.36) | 535151 (218012-1118745) | 43.3 (17.8-89.8) | 424661817 (381241965-468141171) | 21405.93 (19170.73-23452.33) | 147953967 (132280102-165052657) | 7406.48 (6637.36-8238.85) | 825557 (336708-1691734) | 41.16 (16.87-84.59) | -0.12 (-0.13--0.11) | -0.19 (-0.2--0.18) | -0.18 (-0.19--0.17) |
| South Asia | 203298552 (176733718-230322880) | 20297.49 (18084.22-22480.13) | 70580005 (60196621-82570182) | 6758.68 (5942.02-7613.91) | 396310 (160227-848409) | 37.38 (15.34-77.97) | 340937797 (303802441-377408907) | 19696.89 (17580.28-21752.46) | 112096656 (98968557-126418264) | 6425.74 (5692.87-7177.08) | 624823 (254045-1282714) | 35.59 (14.54-73.31) | -0.11 (-0.11--0.1) | -0.18 (-0.19--0.17) | -0.18 (-0.19--0.17) |
| South Asia - WB | 208957680 (181788247-236300592) | 20376.33 (18175.26-22548.65) | 73037682 (62478861-85037568) | 6837 (6023.58-7677.58) | 410130 (165791-873591) | 37.83 (15.52-78.81) | 350079692 (312244129-387141799) | 19649.81 (17580.29-21676.64) | 115832321 (102444320-130260199) | 6445.41 (5725.16-7178.18) | 645668 (262560-1324171) | 35.69 (14.58-73.42) | -0.13 (-0.13--0.12) | -0.21 (-0.22--0.2) | -0.21 (-0.22--0.2) |
| Southeast Asia | 115358590 (102121506-130391433) | 27743.17 (24856.63-30910.52) | 46082263 (40551625-51809381) | 10927.33 (9742.28-12188.32) | 259714 (104256-544692) | 61.03 (24.55-126.22) | 193137286 (172966376-215997244) | 27579.96 (24668.34-30727.02) | 76956034 (68120672-86649851) | 10853.49 (9663.93-12128.52) | 431548 (172611-897329) | 60.72 (24.38-125.8) | -0.02 (-0.02--0.02) | -0.02 (-0.02--0.02) | -0.01 (-0.02--0.01) |
| Southern Africa | 27027558 (23966253-30526444) | 32358.86 (29044.81-35774.16) | 10863449 (9415265-12397122) | 12272.66 (11020.02-13690.25) | 61165 (24995-127166) | 68.09 (27.82-141.44) | 53983086 (47980638-60698614) | 32683.21 (29304.15-36240.43) | 21580159 (18765109-24708255) | 12438.04 (11161.11-13862.47) | 121421 (49802-254262) | 69 (28.41-143.7) | 0.03 (0.01-0.05) | 0.06 (0.04-0.09) | 0.07 (0.04-0.1) |
| Southern Latin America | 9191646 (8214521-10175455) | 19304.5 (17294.94-21354.44) | 2822685 (2555173-3120833) | 5936.26 (5383.2-6543.33) | 15630 (6436-31902) | 32.77 (13.52-66.82) | 14555969 (12965731-16156392) | 19065.44 (17057-21112.96) | 4460228 (4044420-4938312) | 5844.86 (5296.87-6447.14) | 24408 (10057-49802) | 32.18 (13.23-65.53) | -0.04 (-0.04--0.03) | -0.05 (-0.05--0.04) | -0.05 (-0.06--0.05) |
| Southern Sub-Saharan Africa | 13521027 (11994701-15111177) | 29964.42 (26793.59-33142.98) | 4659631 (4084182-5298717) | 10199.06 (9121.68-11313.62) | 26115 (10618-54508) | 56.37 (22.84-116.48) | 21931573 (19503932-24407963) | 29868.53 (26669.82-33040.36) | 7487199 (6627186-8370290) | 10149.03 (9087.13-11253.98) | 41566 (16769-86104) | 55.83 (22.63-115.35) | -0.02 (-0.04-0) | -0.02 (-0.04--0.01) | -0.04 (-0.06--0.02) |
| Sub-Saharan Africa - WB | 175526122 (154653600-199910291) | 36605.69 (32819.2-40471.31) | 82045387 (70348056-94596402) | 15986.53 (14269.23-17767.68) | 463043 (188452-972633) | 88.95 (36.35-185.98) | 395716669 (349557224-447801484) | 36436.95 (32592.05-40374.93) | 176866325 (152824369-202294874) | 15445.53 (13829.56-17199.53) | 1000445 (411287-2103921) | 86.15 (35.17-179.26) | -0.04 (-0.06--0.03) | -0.15 (-0.17--0.13) | -0.14 (-0.16--0.12) |
| Tropical Latin America | 34617218 (31130825-38436576) | 26587.82 (23989.04-29433.91) | 11346064 (10186238-12721823) | 8710.4 (7904.85-9581.79) | 63093 (26074-128836) | 47.77 (19.91-97.74) | 62653211 (56393206-69558553) | 26302.46 (23768.2-29078.3) | 20410381 (18525634-22553587) | 8597.1 (7778.7-9470.52) | 111686 (46262-230126) | 47.16 (19.61-96.35) | -0.03 (-0.03--0.03) | -0.04 (-0.04--0.04) | -0.03 (-0.04--0.03) |
| Western Africa | 65596515 (57923360-73743889) | 40335.05 (36031.52-44590.61) | 30289075 (26268562-34601026) | 18135.91 (16323.22-20185.43) | 170531 (69734-359187) | 100.78 (41.33-209.73) | 162836142 (143559767-183779322) | 40344.71 (36124.95-44591.97) | 75092254 (65439755-85555819) | 18032.68 (16222.08-20063.87) | 424613 (173328-897746) | 100.62 (41.23-208.92) | -0.03 (-0.05--0.02) | -0.07 (-0.09--0.05) | -0.05 (-0.07--0.03) |
| Western Europe | 106491280 (95244578-118463538) | 23364.9 (20946.31-25800.22) | 34948190 (31844285-38672103) | 7671.87 (6968.85-8455.47) | 191282 (79150-395017) | 42.38 (17.45-86.65) | 143367679 (127506138-160700549) | 23177.13 (20787.65-25596.06) | 47550475 (43317751-53001043) | 7634.36 (6923.31-8402.41) | 257296 (106636-533552) | 42.18 (17.37-86.38) | -0.03 (-0.03--0.02) | -0.02 (-0.03--0.01) | -0.02 (-0.02--0.01) |
| Western Pacific Region | 243631079 (218329213-271476955) | 17158.44 (15516.93-18942.9) | 78984272 (70845734-88209513) | 5541.34 (5028.91-6117.25) | 443484 (178676-929384) | 30.86 (12.56-64.2) | 371505954 (334803106-409719289) | 17147.46 (15490.37-18989.78) | 120288479 (109037898-132635272) | 5619.6 (5069.1-6231.34) | 666469 (272937-1384519) | 31.35 (12.74-65.62) | -0.01 (-0.02-0) | 0.04 (0.03-0.04) | 0.04 (0.04-0.05) |
| Western Sub-Saharan Africa | 71560397 (63176588-80573420) | 39838.82 (35618.21-44047.35) | 32786956 (28406632-37527632) | 17742.63 (15939.65-19747.09) | 184631 (75465-388627) | 98.61 (40.42-205.37) | 179438295 (158387265-202499099) | 39705.22 (35574.63-43841.03) | 81750648 (71321336-93056770) | 17516.41 (15769.24-19474.17) | 462278 (188745-975268) | 97.72 (40.06-202.83) | -0.04 (-0.06--0.03) | -0.09 (-0.11--0.07) | -0.07 (-0.09--0.05) |
| World Bank High Income | 202863586 (183260158-223318645) | 17663.59 (16020.13-19360.98) | 64579680 (59520848-70560638) | 5629.14 (5178.7-6117.99) | 345999 (143226-704233) | 31.04 (12.75-63.06) | 293920944 (264955954-325605604) | 17027.64 (15488.38-18597.98) | 93925103 (86760113-103309951) | 5414.45 (4997.24-5887.63) | 498779 (206439-1014754) | 29.83 (12.3-60.42) | -0.13 (-0.13--0.12) | -0.13 (-0.14--0.13) | -0.13 (-0.14--0.13) |
| World Bank Low Income | 94070134 (82414880-107794217) | 31690.06 (28481.98-35174.28) | 46385731 (39134197-54013421) | 14179.16 (12539.24-15850.76) | 281067 (114782-587336) | 79.08 (32.41-164.55) | 202709486 (178004977-230261760) | 31408.97 (28170-34743.96) | 92082081 (78204432-105483904) | 13321.38 (11818.28-14824.84) | 551238 (228540-1158402) | 74.39 (30.66-154.87) | -0.05 (-0.07--0.04) | -0.24 (-0.25--0.22) | -0.23 (-0.24--0.21) |
| World Bank Lower Middle Income | 412609476 (366464795-458755619) | 22989.88 (20673.72-25181.88) | 154081351 (135679584-176526559) | 8314.39 (7495.35-9264.48) | 836677 (342294-1748834) | 46.14 (19.01-95.4) | 749826687 (672159595-825759318) | 23249.61 (20932.38-25441.18) | 277548454 (249058958-310509970) | 8474.55 (7654.87-9420.28) | 1503738 (612200-3093686) | 47.19 (19.35-97.09) | 0.02 (0.02-0.03) | 0.04 (0.03-0.05) | 0.06 (0.05-0.07) |
| World Bank Upper Middle Income | 319120956 (286120726-353569088) | 17686.31 (16026.26-19458.97) | 102175538 (91855554-113671652) | 5636.79 (5127.44-6210.72) | 592484 (240266-1240437) | 31.27 (12.78-64.85) | 481215007 (434646338-530543568) | 17259.89 (15592.17-19029.02) | 152443661 (138246360-168979644) | 5508.88 (4991.35-6096.75) | 872108 (355766-1809371) | 30.57 (12.43-63.54) | -0.09 (-0.1--0.08) | -0.09 (-0.09--0.08) | -0.08 (-0.09--0.08) |
| Country |  |  |  |  |  |  |  |  |  |  |  |  |  |  |  |
| Afghanistan | 911391 (820701-1007870) | 11551.4 (10433.61-12742.64) | 278764 (252021-311291) | 3483.89 (3176.57-3849.97) | 1541 (633-3146) | 18.97 (7.79-38.18) | 2297892 (2055659-2553714) | 11264.94 (10152.53-12465.65) | 707745 (633184-797092) | 3380.87 (3083.03-3730.87) | 3949 (1598-8037) | 18.27 (7.5-37.08) | -0.09 (-0.09--0.08) | -0.11 (-0.12--0.1) | -0.13 (-0.13--0.12) |
| Albania | 496370 (443841-554921) | 18075.34 (16130-20039.06) | 151982 (136776-170633) | 5511.14 (5006.02-6135.44) | 848 (341-1754) | 30.39 (12.33-62) | 604269 (535013-676708) | 18041.96 (16103.31-19991.33) | 182953 (165169-205796) | 5501.63 (4996.6-6129.58) | 1000 (407-2037) | 30.36 (12.4-62.12) | -0.01 (-0.01-0) | 0 (-0.01-0) | 0 (0-0.01) |
| Algeria | 2084041 (1883072-2310587) | 11384.17 (10251.55-12550.92) | 630980 (568872-701387) | 3396.26 (3099.86-3755.82) | 3533 (1418-7272) | 18.66 (7.57-37.94) | 4384047 (3936262-4845571) | 11074.14 (9976.73-12247.47) | 1301131 (1182241-1444426) | 3282.77 (2990.48-3625.09) | 7182 (2929-14489) | 17.95 (7.31-36.3) | -0.09 (-0.09--0.08) | -0.11 (-0.11--0.1) | -0.12 (-0.12--0.12) |
| American Samoa | 7853 (6986-8781) | 19054.9 (17109.63-21152.34) | 2489 (2196-2805) | 5953.24 (5318.14-6659.62) | 14 (6-29) | 33.13 (13.59-67.84) | 9329 (8355-10408) | 18956.26 (17027.76-21071.54) | 2928 (2612-3275) | 5921.72 (5288.11-6617.05) | 16 (7-34) | 32.8 (13.38-67.48) | -0.01 (-0.02--0.01) | -0.01 (-0.02--0.01) | -0.02 (-0.03--0.02) |
| Andorra | 13229 (11749-14676) | 23554.33 (21087.16-26065) | 4384 (3923-4884) | 7841.14 (7069.24-8689.24) | 24 (10-51) | 43.43 (17.81-89.51) | 26051 (23162-29075) | 23317.98 (20854.64-25815.01) | 8694 (7904-9731) | 7754.51 (6986.05-8591.2) | 47 (19-99) | 42.85 (17.65-88.02) | -0.03 (-0.04--0.03) | -0.04 (-0.04--0.03) | -0.04 (-0.05--0.04) |
| Angola | 3054319 (2573362-3690138) | 30876.14 (27080.2-34962.69) | 1626785 (1250098-2053539) | 13978.16 (11490.4-16787.52) | 9267 (3642-20152) | 78.48 (31.59-168.73) | 9218538 (7774679-11139227) | 29821.17 (26260.38-33721.62) | 4436263 (3372026-5600060) | 12266.76 (10208.34-14529.12) | 25292 (10161-55876) | 68.76 (28.26-148.06) | -0.13 (-0.18--0.08) | -0.46 (-0.51--0.42) | -0.46 (-0.51--0.42) |
| Antigua and Barbuda | 14966 (13389-16643) | 25599.93 (22880.37-28502.21) | 4946 (4464-5509) | 8426.34 (7634.29-9368.43) | 27 (11-57) | 46.65 (19.37-96.45) | 24249 (21661-27183) | 25459.42 (22756.9-28344.56) | 7939 (7167-8845) | 8385.52 (7599.56-9306.75) | 44 (18-90) | 46.25 (19.23-95.52) | -0.02 (-0.02--0.01) | -0.01 (-0.02--0.01) | -0.02 (-0.03--0.02) |
| Argentina | 6235115 (5574873-6903437) | 19320.83 (17306.72-21374.21) | 1915768 (1735249-2118156) | 5944.28 (5392.62-6553.9) | 10604 (4376-21633) | 32.83 (13.55-66.93) | 9598724 (8552740-10634311) | 19090.74 (17083.45-21134) | 2943700 (2667023-3250461) | 5857.19 (5305.57-6458.87) | 16141 (6632-32893) | 32.28 (13.24-65.69) | -0.04 (-0.04--0.03) | -0.04 (-0.05--0.04) | -0.05 (-0.06--0.04) |
| Armenia | 553590 (494492-616201) | 18050.88 (16115.63-19999.17) | 168897 (151924-189057) | 5502.75 (4990.37-6132.42) | 939 (378-1953) | 30.35 (12.33-62.3) | 641926 (570877-715037) | 18032.56 (16097.2-19975.61) | 194628 (176349-217897) | 5497.25 (4983.2-6128.33) | 1066 (431-2164) | 30.33 (12.29-62.05) | 0 (0-0) | 0 (0-0) | 0 (0-0.01) |
| Australia | 4420439 (3984337-4868066) | 24531.72 (22121.57-26973.21) | 1573279 (1503873-1645281) | 8704.56 (8306.19-9144.47) | 8624 (3571-17645) | 47.82 (19.8-98.37) | 8053935 (7275653-8937386) | 24358.69 (21960.59-26816.84) | 2975614 (2855101-3088616) | 8654.94 (8262.15-9096.4) | 16106 (6629-32666) | 47.57 (19.55-97.97) | -0.02 (-0.02--0.02) | -0.02 (-0.02--0.01) | -0.02 (-0.03--0.02) |
| Austria | 2182497 (1942347-2435326) | 23550.22 (21062.6-26085.76) | 723338 (654927-801412) | 7806.99 (7051.4-8650.53) | 3955 (1631-8181) | 43.12 (17.75-88.68) | 2883453 (2560376-3233875) | 23318.74 (20860.87-25824.22) | 964289 (876314-1075611) | 7740.21 (6972.45-8581.66) | 5230 (2148-10804) | 42.79 (17.54-87.69) | -0.03 (-0.03--0.03) | -0.02 (-0.03--0.02) | -0.02 (-0.03--0.02) |
| Azerbaijan | 1124263 (1003929-1253501) | 18058.08 (16136.97-20002.49) | 343852 (309756-385341) | 5504.77 (4996.47-6134.98) | 1919 (774-3989) | 30.45 (12.41-62.38) | 1846978 (1653507-2055929) | 18055.4 (16120.08-20004.45) | 560978 (503736-626735) | 5505.24 (4993.65-6133.27) | 3113 (1243-6437) | 30.41 (12.3-62.29) | 0 (0-0) | 0 (0-0) | 0 (0-0) |
| Bahamas | 57089 (50844-63556) | 25816.44 (23096.03-28707.15) | 18833 (16697-21221) | 8526.85 (7725.86-9462.84) | 105 (43-220) | 47.27 (19.65-97.61) | 100785 (89531-112823) | 25629.92 (22926.11-28524.73) | 33077 (29943-36921) | 8462.79 (7667.42-9391.88) | 183 (75-380) | 46.76 (19.44-96.81) | -0.02 (-0.03--0.02) | -0.02 (-0.03--0.02) | -0.03 (-0.04--0.03) |
| Bahrain | 41859 (36970-47093) | 11379.9 (10255.95-12567.02) | 12607 (11113-14351) | 3389.76 (3074.94-3737.13) | 71 (28-144) | 18.59 (7.53-37.76) | 144973 (128670-163454) | 11088.77 (9941.36-12282.83) | 42916 (38330-48333) | 3279.68 (2980.16-3622.89) | 239 (97-486) | 17.87 (7.3-36.2) | -0.08 (-0.09--0.08) | -0.11 (-0.11--0.1) | -0.12 (-0.13--0.12) |
| Bangladesh | 18730315 (16353172-21302562) | 19403.68 (17386.15-21470.59) | 6576614 (5532734-7846717) | 6425.81 (5651.32-7255.09) | 37084 (14736-80425) | 35.67 (14.54-73.58) | 28941648 (25891405-32150688) | 18606.29 (16744.26-20598.61) | 9255261 (8265702-10368087) | 5914.87 (5297.74-6578.37) | 51670 (21191-106835) | 32.86 (13.43-68.05) | -0.15 (-0.15--0.14) | -0.29 (-0.3--0.28) | -0.29 (-0.3--0.28) |
| Barbados | 69213 (61861-77429) | 25625.74 (22910.41-28544.78) | 22904 (20648-25558) | 8438.57 (7648.29-9355.7) | 127 (52-261) | 46.8 (19.39-96.71) | 95311 (85137-107584) | 25416.37 (22714.25-28315.34) | 31430 (28241-35301) | 8365.09 (7578.84-9302.38) | 172 (71-354) | 46.23 (19.14-95.56) | -0.02 (-0.03--0.02) | -0.02 (-0.03--0.02) | -0.03 (-0.04--0.03) |
| Belarus | 2050974 (1826668-2283676) | 18022.35 (16101.12-19960.64) | 622483 (565924-694625) | 5491.5 (4986.63-6118.04) | 3417 (1383-6971) | 30.25 (12.29-61.82) | 2180007 (1930938-2440399) | 18008.08 (16085.77-19950.57) | 659596 (598292-740295) | 5486.01 (4975.23-6114.82) | 3584 (1453-7226) | 30.19 (12.24-61.51) | 0 (0-0) | 0 (-0.01-0) | 0 (0-0) |
| Belgium | 2806138 (2497313-3127398) | 23535.15 (21064.58-26041.03) | 931343 (844290-1034702) | 7809.5 (7054.51-8654.52) | 5097 (2103-10537) | 43.15 (17.8-88.54) | 3681844 (3272166-4124691) | 23317.46 (20870.61-25824.89) | 1235016 (1126796-1376649) | 7743.14 (6972.26-8585.26) | 6675 (2758-13811) | 42.72 (17.58-87.27) | -0.03 (-0.03--0.03) | -0.03 (-0.03--0.02) | -0.03 (-0.04--0.03) |
| Belize | 39088 (34739-43714) | 25671.65 (22982.27-28601.42) | 12936 (11544-14540) | 8467.82 (7682.78-9407.38) | 73 (30-150) | 46.99 (19.61-97.31) | 97972 (87718-109532) | 25564.65 (22874.19-28519.26) | 32227 (28787-36128) | 8442.34 (7655.71-9366.49) | 180 (74-375) | 46.66 (19.35-96.35) | -0.02 (-0.02--0.01) | -0.02 (-0.02--0.01) | -0.03 (-0.03--0.02) |
| Benin | 1650073 (1426056-1926225) | 36698.92 (32687.63-41067.11) | 733690 (607786-880560) | 15284.1 (13295.11-17262.5) | 4148 (1645-8792) | 85.07 (34.13-179.65) | 4394861 (3803976-5070002) | 36191.95 (32246.86-40436.66) | 1855405 (1557969-2183185) | 14594.52 (12831.13-16441.01) | 10505 (4267-22073) | 81.38 (32.81-171.04) | -0.06 (-0.07--0.04) | -0.16 (-0.17--0.15) | -0.15 (-0.17--0.14) |
| Bermuda | 15455 (13746-17288) | 25484 (22756.2-28371.58) | 5050 (4553-5658) | 8374.18 (7587.97-9302.33) | 28 (12-58) | 46.49 (19.3-96.21) | 22560 (20081-25559) | 25137.13 (22450.92-28071.55) | 7467 (6723-8346) | 8248.28 (7453.58-9184.45) | 41 (17-84) | 45.66 (18.94-94.18) | -0.04 (-0.05--0.03) | -0.04 (-0.05--0.04) | -0.05 (-0.06--0.04) |
| Bhutan | 103829 (91550-117890) | 19260.24 (17216.21-21308.47) | 36000 (30541-42145) | 6324.85 (5579.61-7079.87) | 204 (82-437) | 35.2 (14.26-73.18) | 132349 (119157-147496) | 18646.69 (16782.92-20594.74) | 42180 (37724-47103) | 5934.05 (5295.23-6609.57) | 236 (97-478) | 33.01 (13.48-67.49) | -0.12 (-0.12--0.11) | -0.23 (-0.24--0.21) | -0.23 (-0.24--0.21) |
| Bolivia (Plurinational State of) | 1675611 (1495048-1866323) | 31450.09 (28205.67-34938.46) | 651145 (576733-731482) | 12291.97 (11061.27-13611.62) | 3648 (1487-7489) | 67.94 (27.83-141.23) | 3391917 (3030341-3766296) | 31039.4 (27808.32-34521.25) | 1320272 (1173417-1473602) | 12134.13 (10883.42-13423.83) | 7341 (2987-15261) | 67 (27.42-139.49) | -0.04 (-0.05--0.04) | -0.04 (-0.05--0.04) | -0.04 (-0.05--0.04) |
| Bosnia and Herzegovina | 768425 (690110-854342) | 18041.02 (16099.29-19988.69) | 233741 (210084-261321) | 5499.91 (4990.5-6123.65) | 1294 (518-2672) | 30.25 (12.26-61.89) | 809701 (714405-906148) | 18026.56 (16084.42-19975.42) | 244858 (221218-274995) | 5495.26 (4985.32-6120.54) | 1323 (538-2687) | 30.17 (12.18-61.66) | 0 (0-0) | 0 (0-0) | 0 (0-0) |
| Botswana | 322347 (284468-365241) | 29349.7 (26163.58-32515.97) | 113626 (98756-130821) | 10153.24 (9141.48-11264.39) | 640 (261-1341) | 56.27 (22.83-115.16) | 620935 (552668-691094) | 29234.58 (26022.21-32355.71) | 215277 (190185-243677) | 10073.84 (9081.09-11242.54) | 1201 (488-2520) | 55.56 (22.52-114.83) | -0.03 (-0.05--0.01) | -0.05 (-0.07--0.03) | -0.06 (-0.08--0.04) |
| Brazil | 33754760 (30357571-37479822) | 26612.5 (24004.35-29478.59) | 11061557 (9924560-12400502) | 8716.84 (7908.83-9591.53) | 61503 (25424-125545) | 47.8 (19.92-97.77) | 60942534 (54876447-67703857) | 26326.71 (23782.63-29124.37) | 19847606 (17999176-21931557) | 8603.02 (7784.53-9473.39) | 108572 (44952-223665) | 47.19 (19.63-96.37) | -0.03 (-0.03--0.03) | -0.04 (-0.04--0.04) | -0.03 (-0.04--0.03) |
| Brunei Darussalam | 38740 (34373-43430) | 19427.9 (17379.94-21501.7) | 12070 (10712-13610) | 6029.73 (5460.47-6642.83) | 68 (28-140) | 33.28 (13.73-68.16) | 78694 (70096-87589) | 19175.23 (17157.83-21245.14) | 24054 (21574-26894) | 5917.96 (5369.88-6518.72) | 134 (55-275) | 32.63 (13.38-66.32) | -0.04 (-0.05--0.03) | -0.05 (-0.06--0.04) | -0.06 (-0.06--0.05) |
| Bulgaria | 1784012 (1579734-1998948) | 18053.02 (16118.27-19997.58) | 539165 (486713-606674) | 5505.35 (4998.7-6131.81) | 2955 (1207-6041) | 30.29 (12.36-62.05) | 1782157 (1565820-2011136) | 18028.33 (16086.24-19983.42) | 538370 (485520-608374) | 5495.2 (4982.59-6119.69) | 2911 (1195-5954) | 30.25 (12.32-62.26) | 0 (0-0) | -0.01 (-0.01--0.01) | 0 (0-0) |
| Burkina Faso | 3180292 (2746586-3655012) | 36275.57 (32339.25-40260.12) | 1377349 (1143506-1652073) | 14870.38 (13074.32-16770.45) | 7757 (3160-16371) | 82.6 (33.5-173.07) | 7441890 (6453018-8590221) | 36174.32 (32307.05-40418.4) | 3136020 (2628669-3685380) | 14592.19 (12839.54-16493) | 17752 (7147-37430) | 81.5 (33.01-172.74) | -0.02 (-0.04--0.01) | -0.08 (-0.1--0.06) | -0.06 (-0.08--0.04) |
| Burundi | 1935746 (1684418-2264173) | 36838.75 (33038.26-41177.44) | 889914 (735257-1076870) | 15603.17 (13695.29-17879.67) | 5033 (2023-10717) | 87.04 (36.38-183.82) | 4395881 (3808028-5058524) | 36419.9 (32523.65-40628.65) | 1908562 (1594759-2254425) | 14882.26 (13130.99-16870.33) | 10816 (4433-23009) | 83.11 (34.57-176.4) | -0.05 (-0.06--0.04) | -0.16 (-0.17--0.15) | -0.15 (-0.16--0.14) |
| Cabo Verde | 119573 (104274-136518) | 35555.87 (31624.1-39718.98) | 49159 (42286-57342) | 14173.12 (12505.13-15987.41) | 277 (112-589) | 79.32 (32.11-165.99) | 185655 (164449-208004) | 34987.35 (31009.64-38918.24) | 72612 (63879-82027) | 13574.47 (11959.82-15250.6) | 407 (166-860) | 75.8 (30.76-158.81) | -0.08 (-0.09--0.07) | -0.19 (-0.2--0.17) | -0.19 (-0.21--0.18) |
| Cambodia | 2323782 (2070123-2630712) | 26950.29 (24245.42-30273.14) | 927146 (812753-1047648) | 10661.25 (9506.52-12006.05) | 5217 (2095-10976) | 59.3 (23.97-123.86) | 4282307 (3831025-4838193) | 26674.35 (23983.35-30026.21) | 1716842 (1515147-1944545) | 10554.86 (9409.11-11879.2) | 9643 (3862-20399) | 58.89 (23.8-123.12) | -0.04 (-0.04--0.04) | -0.04 (-0.04--0.04) | -0.03 (-0.03--0.02) |
| Cameroon | 3179485 (2776077-3647739) | 34055.28 (30530.53-37969.11) | 1274696 (1080760-1500692) | 13058.4 (11588.98-14723.68) | 7196 (2919-15277) | 72.56 (29.76-152.83) | 9297579 (8195537-10473955) | 33389.19 (30036.3-36935.48) | 3530601 (3229561-3847104) | 12346.77 (11381.29-13515.38) | 19951 (8074-41683) | 68.67 (28.08-141.95) | -0.06 (-0.08--0.05) | -0.15 (-0.17--0.12) | -0.14 (-0.16--0.12) |
| Canada | 2614869 (2337886-2914567) | 8831.07 (7949.35-9829.09) | 741289 (667273-827821) | 2517.31 (2268.41-2807.4) | 4083 (1657-8402) | 13.91 (5.65-28.73) | 4586339 (4069374-5159299) | 8757.87 (7878.4-9743.23) | 1290901 (1163522-1457562) | 2490 (2245.31-2781.64) | 6980 (2821-14196) | 13.71 (5.58-27.76) | -0.03 (-0.03--0.02) | -0.03 (-0.04--0.03) | -0.04 (-0.05--0.04) |
| Central African Republic | 821972 (695478-974827) | 31160.84 (27474.69-35279.66) | 443528 (339654-564116) | 14354.93 (11797.86-17324.26) | 2514 (1020-5441) | 80.19 (32.81-172.76) | 1558808 (1341037-1844417) | 30531.77 (26995.13-34417.71) | 796228 (622090-998480) | 13467 (11189.3-16029.56) | 4522 (1826-9829) | 75.35 (30.77-160.61) | -0.07 (-0.12--0.03) | -0.23 (-0.27--0.2) | -0.23 (-0.26--0.19) |
| Chad | 2035732 (1763013-2352373) | 36479.99 (32525.42-40722.6) | 888782 (734565-1050281) | 15096.93 (13224.43-17023.28) | 5013 (2042-10811) | 84.03 (34.4-175.95) | 5803054 (4975671-6750772) | 36294.21 (32227.8-40556.31) | 2510830 (2090657-2973193) | 14803.02 (13023.21-16660.69) | 14223 (5779-30048) | 82.48 (33.78-172.73) | -0.03 (-0.04--0.01) | -0.09 (-0.11--0.06) | -0.08 (-0.11--0.06) |
| Chile | 2298470 (2060606-2550066) | 19276.62 (17269.45-21324.62) | 705379 (634492-786863) | 5923.18 (5370.83-6526.81) | 3917 (1605-8032) | 32.64 (13.44-66.53) | 4130943 (3682013-4584241) | 19006.05 (16996.17-21065.71) | 1262592 (1144727-1397616) | 5815.95 (5276.32-6419.62) | 6887 (2852-14115) | 31.96 (13.17-65.16) | -0.05 (-0.05--0.04) | -0.06 (-0.07--0.05) | -0.07 (-0.07--0.06) |
| China | 162417621 (145294297-182471885) | 14836.44 (13414.65-16503.51) | 50395632 (44888051-56527047) | 4579.55 (4122.36-5088.01) | 283873 (114080-594497) | 25.6 (10.39-53.33) | 229353300 (205924082-254932663) | 14263.04 (12845.6-15855.07) | 69601875 (62751479-77349821) | 4363.55 (3920.72-4851.33) | 386453 (157258-805104) | 24.37 (9.87-50.94) | -0.15 (-0.16--0.14) | -0.18 (-0.19--0.17) | -0.19 (-0.19--0.18) |
| Colombia | 4947280 (4454505-5490072) | 18463.36 (16597.82-20404.04) | 1490082 (1341128-1666971) | 5496.55 (4971.99-6093.81) | 8348 (3394-17176) | 30.35 (12.33-62.61) | 9346671 (8367805-10367897) | 18067.85 (16201.99-19969.56) | 2755096 (2494697-3056513) | 5341.75 (4835.46-5928.29) | 15166 (6198-31282) | 29.51 (12.05-60.72) | -0.07 (-0.07--0.06) | -0.09 (-0.1--0.08) | -0.08 (-0.09--0.08) |
| Comoros | 158551 (138334-181152) | 36694.5 (32880.54-40867.69) | 71454 (59373-86064) | 15359.99 (13501.15-17475.09) | 405 (166-862) | 85.73 (35.51-180.05) | 251775 (222932-284082) | 36385.33 (32636.78-40726.04) | 105018 (90393-121933) | 14849.35 (13045.75-17025.53) | 592 (247-1261) | 82.96 (34.54-176.05) | -0.05 (-0.06--0.04) | -0.13 (-0.16--0.1) | -0.12 (-0.15--0.09) |
| Congo | 720429 (615778-855725) | 31098.75 (27312.27-35231.02) | 387895 (300249-488799) | 14245.84 (11687.41-17050.64) | 2208 (889-4791) | 80.01 (32.35-174.32) | 1470116 (1259987-1708890) | 29838.85 (26243.63-33686.02) | 667141 (524301-823398) | 12334.52 (10289.47-14561.25) | 3786 (1519-8063) | 69.16 (28.34-147.53) | -0.16 (-0.21--0.11) | -0.55 (-0.6--0.5) | -0.56 (-0.6--0.51) |
| Cook Islands | 3227 (2869-3604) | 18899.54 (16983.88-21014.28) | 1015 (897-1141) | 5881.95 (5242.96-6575.44) | 6 (2-12) | 32.74 (13.23-67.25) | 3649 (3259-4065) | 18546.19 (16656.99-20672.72) | 1122 (1005-1253) | 5742.85 (5122.72-6401.29) | 6 (3-13) | 31.87 (12.85-66.15) | -0.06 (-0.07--0.06) | -0.08 (-0.08--0.07) | -0.09 (-0.09--0.08) |
| Costa Rica | 464636 (418256-516492) | 18199.89 (16343.22-20107.92) | 138737 (125040-155199) | 5380.75 (4872.43-5965.37) | 777 (316-1595) | 29.75 (12.08-61.29) | 910733 (816093-1012234) | 18016.14 (16168.67-19929.39) | 268081 (242638-296913) | 5320.58 (4813.05-5902.83) | 1472 (600-3042) | 29.34 (11.96-60.85) | -0.03 (-0.04--0.03) | -0.04 (-0.04--0.04) | -0.05 (-0.05--0.04) |
| Croatia | 953102 (847939-1064708) | 18018.9 (16085.38-19960.82) | 288702 (261242-321773) | 5490.29 (4981.25-6114.89) | 1585 (644-3220) | 30.2 (12.37-61.75) | 1114889 (984075-1252728) | 18020.13 (16078.59-19971.57) | 337426 (305079-378805) | 5491.9 (4980.13-6118.39) | 1817 (743-3679) | 30.2 (12.31-61.91) | 0 (0-0) | 0 (0-0) | 0 (0-0.01) |
| Cuba | 2723887 (2431849-3048353) | 25457.15 (22750.74-28361.71) | 894792 (806911-1002036) | 8375.94 (7599.94-9311.7) | 4965 (2049-10286) | 46.39 (19.29-95.5) | 3610912 (3232527-4062522) | 25244.19 (22558.22-28177.1) | 1191568 (1071425-1332121) | 8294.92 (7504.76-9232.73) | 6496 (2657-13432) | 45.83 (18.97-94.78) | -0.02 (-0.03--0.02) | -0.03 (-0.03--0.02) | -0.03 (-0.04--0.03) |
| Cyprus | 184801 (164792-205267) | 23594.84 (21120.26-26097.57) | 61187 (55002-67779) | 7849.2 (7086.83-8690.68) | 339 (140-701) | 43.38 (17.86-89.1) | 379143 (337379-421578) | 23299.18 (20851.53-25780.19) | 125707 (113794-140103) | 7741.22 (6973.36-8579.39) | 690 (285-1422) | 42.83 (17.65-87.73) | -0.04 (-0.05--0.04) | -0.05 (-0.06--0.04) | -0.05 (-0.05--0.04) |
| Czechia | 2108803 (1874759-2344905) | 18029.08 (16098.53-19976.69) | 639504 (581438-711940) | 5493.96 (4981.5-6120.89) | 3482 (1414-7112) | 30.09 (12.2-61.54) | 2728022 (2410636-3063962) | 18026.97 (16082.08-19981.72) | 826178 (746969-930752) | 5494.41 (4984.76-6120.06) | 4445 (1820-9029) | 30.15 (12.23-61.57) | 0 (0-0) | 0 (0-0) | 0.01 (0.01-0.01) |
| C么te d'Ivoire | 3934689 (3400495-4515744) | 36261.66 (32322.65-40281.42) | 1695753 (1545117-1858210) | 14798.8 (13593.79-16102.68) | 9558 (3876-19944) | 81.88 (33.2-169.78) | 9202565 (8087660-10529319) | 36587.35 (32690.29-40814.1) | 3856510 (3511563-4202935) | 14732.22 (13600.55-16049.45) | 21809 (8915-45553) | 82.15 (33.43-170.05) | -0.06 (-0.08--0.03) | -0.16 (-0.21--0.12) | -0.14 (-0.19--0.1) |
| Democratic People's Republic of Korea | 2691297 (2425346-3006664) | 13886.62 (12580.58-15378.68) | 802351 (720376-894822) | 4126.23 (3728.64-4581.37) | 4519 (1840-9382) | 23.1 (9.41-47.43) | 3896976 (3524817-4337385) | 13682.18 (12377.01-15148.11) | 1150253 (1038835-1277204) | 4069.04 (3677.41-4509.91) | 6424 (2638-13197) | 22.78 (9.33-46.66) | -0.06 (-0.06--0.05) | -0.05 (-0.06--0.05) | -0.05 (-0.06--0.05) |
| Democratic Republic of the Congo | 11327898 (9500798-13727269) | 30669.08 (27057.86-34835.68) | 5930733 (4531591-7580797) | 13655.31 (11279.65-16366.96) | 33597 (13426-73146) | 76.16 (30.58-164.07) | 24763438 (20883299-29435486) | 29680.11 (26100.11-33428.62) | 11446990 (8951292-14136384) | 12043.38 (10095.56-14170.65) | 65018 (26540-140063) | 67.37 (27.6-143.6) | -0.12 (-0.16--0.08) | -0.43 (-0.46--0.41) | -0.42 (-0.44--0.39) |
| Denmark | 1473160 (1315329-1642318) | 23591.81 (21120.51-26089.53) | 490345 (444230-543225) | 7844.05 (7084.96-8690.14) | 2680 (1112-5542) | 43.35 (17.89-89.24) | 1862125 (1646226-2095181) | 23343.03 (20868.84-25843.04) | 623325 (565961-697009) | 7756.66 (6989.74-8599) | 3399 (1402-7069) | 42.97 (17.68-88.61) | -0.04 (-0.04--0.03) | -0.04 (-0.04--0.04) | -0.03 (-0.03--0.02) |
| Djibouti | 135714 (117455-155757) | 36579.28 (32628.73-40758.25) | 59829 (49879-71684) | 15132.49 (13258.22-17333.52) | 340 (139-714) | 84.61 (35.27-175.3) | 410354 (361401-463359) | 36309.74 (32475.76-40510.96) | 170568 (146915-200022) | 14729.99 (12981.46-16835.04) | 964 (399-2010) | 82.34 (34.1-172.37) | -0.03 (-0.05--0.02) | -0.09 (-0.13--0.06) | -0.09 (-0.13--0.05) |
| Dominica | 17400 (15620-19377) | 25778.57 (23038.01-28690.58) | 5760 (5202-6439) | 8505.87 (7691.54-9439.44) | 32 (13-66) | 47.13 (19.6-97.97) | 18691 (16705-20923) | 25773.5 (23090.48-28691.62) | 6166 (5578-6881) | 8527.07 (7719.28-9469.76) | 34 (14-70) | 47.09 (19.48-97.3) | 0 (0-0.01) | 0.01 (0.01-0.02) | 0 (0-0.01) |
| Dominican Republic | 1518846 (1350885-1701314) | 25804.16 (23138.2-28690.6) | 501525 (444331-567754) | 8532.98 (7732.86-9462.48) | 2814 (1161-5863) | 47.3 (19.55-98.06) | 2734357 (2438009-3049757) | 25618.29 (22922.82-28546.8) | 901044 (811481-1003292) | 8462.09 (7668.75-9397.66) | 4987 (2061-10349) | 46.73 (19.39-96.83) | -0.02 (-0.02--0.01) | -0.02 (-0.03--0.01) | -0.03 (-0.03--0.02) |
| Ecuador | 2647319 (2360241-2948081) | 31220.03 (27989.21-34744.52) | 1029620 (906904-1158110) | 12183.17 (10943.01-13472) | 5774 (2359-11799) | 67.55 (27.59-140.16) | 5414956 (4838183-6024919) | 30810.42 (27590.09-34300.23) | 2111780 (1877954-2357799) | 12021.63 (10775.85-13319.53) | 11712 (4771-24299) | 66.5 (27.15-138.33) | -0.04 (-0.04--0.03) | -0.04 (-0.04--0.03) | -0.04 (-0.05--0.04) |
| Egypt | 4968460 (4471227-5469557) | 11904.72 (10776.29-13062.48) | 1599067 (1469360-1757634) | 3688.78 (3396.68-4068.78) | 8978 (3654-18318) | 20.32 (8.41-41.25) | 9106487 (8245885-9998301) | 11128.49 (10044.14-12215.58) | 2717076 (2465998-2990054) | 3300.77 (3008.27-3653.17) | 15121 (6150-31179) | 18.05 (7.39-36.52) | -0.15 (-0.17--0.12) | -0.22 (-0.27--0.18) | -0.23 (-0.28--0.19) |
| El Salvador | 823012 (738304-912568) | 18521.11 (16656.94-20464.34) | 249583 (224343-278486) | 5526.32 (5001.24-6138.84) | 1393 (569-2872) | 30.48 (12.38-62.69) | 1166119 (1047969-1288271) | 18109.65 (16264.31-20034.49) | 346198 (312872-384072) | 5362.33 (4844.7-5952.18) | 1907 (780-3954) | 29.56 (12.07-61.19) | -0.07 (-0.08--0.06) | -0.09 (-0.11--0.08) | -0.09 (-0.11--0.08) |
| Equatorial Guinea | 129283 (109515-156455) | 31037.47 (27265.27-35141.33) | 69040 (52889-86819) | 14124.58 (11497.52-16815.04) | 390 (157-839) | 78.7 (32.08-166.45) | 397291 (337521-467848) | 29403.44 (25912.19-33097.55) | 177400 (138205-221247) | 11660.41 (9805.95-13840.07) | 1008 (407-2175) | 65.18 (26.62-139.57) | -0.21 (-0.25--0.17) | -0.72 (-0.75--0.68) | -0.7 (-0.74--0.67) |
| Eritrea | 1163190 (997369-1357586) | 37002.76 (33130.59-41351.23) | 540570 (441347-651574) | 15695.65 (13691.27-17913.16) | 3055 (1221-6469) | 87.27 (36.01-181.65) | 2200811 (1921988-2522963) | 36821.44 (32807.73-41123.25) | 963165 (811253-1131733) | 15264.74 (13422.87-17486.38) | 5454 (2239-11543) | 85.15 (35.28-177.03) | -0.02 (-0.04--0.01) | -0.07 (-0.09--0.05) | -0.06 (-0.08--0.03) |
| Estonia | 316638 (281991-351839) | 18022.04 (16107.37-19963.28) | 95973 (87570-106853) | 5490.71 (4983.88-6120.48) | 525 (214-1067) | 30.19 (12.26-61.6) | 344954 (304731-385657) | 18022.25 (16091.72-19967.71) | 104603 (94871-117520) | 5491.11 (4980.05-6116.6) | 565 (231-1143) | 30.26 (12.22-61.72) | 0 (0-0) | 0 (0-0) | 0.01 (0.01-0.01) |
| Eswatini | 192770 (170198-217743) | 29292.14 (26206.47-32373.83) | 67829 (58613-78366) | 10105.03 (9079.29-11263.36) | 384 (155-807) | 56.1 (22.71-115.21) | 289162 (256985-324692) | 29390.85 (26189.2-32575.52) | 101258 (88453-115218) | 10147.98 (9138.53-11250.09) | 567 (231-1196) | 55.84 (22.64-115.14) | 0.01 (-0.01-0.02) | 0.01 (-0.01-0.04) | -0.01 (-0.04-0.01) |
| Ethiopia | 26073170 (22545859-30487980) | 49542.74 (44237.02-55641.14) | 15434199 (13040302-18125099) | 26536.98 (23195.87-29871.65) | 87523 (35406-179991) | 148.43 (60.29-307.88) | 49754827 (43552428-56873684) | 47244.75 (42242.21-52965.46) | 25775618 (21624531-29836686) | 23086.22 (20202.85-25968.55) | 146226 (59500-304555) | 129.28 (52.43-270.08) | -0.2 (-0.22--0.19) | -0.51 (-0.53--0.49) | -0.5 (-0.51--0.48) |
| Fiji | 125563 (111597-139993) | 19114.29 (17192.32-21242.81) | 39980 (35114-45100) | 5984.31 (5345.21-6687.01) | 225 (91-462) | 33.25 (13.53-68) | 167833 (150810-187586) | 19064.64 (17152.95-21199.79) | 52917 (46933-59306) | 5979.53 (5346.26-6684.35) | 295 (120-607) | 33.17 (13.54-67.83) | -0.01 (-0.01-0) | 0 (0-0) | 0 (-0.01-0) |
| Finland | 1366132 (1216969-1517080) | 23589 (21081.45-26126.88) | 452374 (409915-501167) | 7826.81 (7067.13-8671.87) | 2473 (1019-5083) | 43.14 (17.77-88.15) | 1883111 (1660920-2122131) | 23325.87 (20872.08-25823.1) | 632587 (572210-707678) | 7744.32 (6978.16-8586.38) | 3408 (1406-7088) | 42.71 (17.58-87.58) | -0.04 (-0.04--0.03) | -0.03 (-0.04--0.03) | -0.03 (-0.04--0.02) |
| France | 15834021 (14103952-17572417) | 23554.99 (21081.48-26053.06) | 5266321 (4779499-5839686) | 7822.11 (7061.97-8661.75) | 28785 (11862-59236) | 43.17 (17.73-88.41) | 21708487 (19296928-24400237) | 23297.79 (20845.4-25784.43) | 7297650 (6645461-8137054) | 7731.53 (6963.18-8574.2) | 39409 (16250-81729) | 42.69 (17.64-87.34) | -0.04 (-0.04--0.03) | -0.04 (-0.04--0.03) | -0.04 (-0.04--0.03) |
| Gabon | 292636 (253010-344194) | 30488.07 (27176.78-34526.45) | 146127 (139359-153343) | 13468.46 (12784.78-14208) | 826 (338-1723) | 75.33 (30.93-155.12) | 481780 (429921-555626) | 28921.47 (26076.62-32376.2) | 210480 (199322-221981) | 11659.5 (10993.18-12357.65) | 1189 (485-2446) | 65.12 (26.68-133.13) | -0.11 (-0.19--0.04) | -0.52 (-0.58--0.45) | -0.52 (-0.59--0.45) |
| Gambia | 319260 (274842-367127) | 36278.25 (32378.04-40529.32) | 138618 (114498-163829) | 14871.88 (13008.45-16613.88) | 785 (318-1676) | 82.97 (33.91-177.42) | 771903 (673731-877933) | 36005.86 (32062.7-40033.02) | 324302 (272275-376563) | 14489.99 (12764.11-16265.71) | 1832 (755-3934) | 80.71 (32.94-169.65) | -0.04 (-0.05--0.03) | -0.1 (-0.12--0.09) | -0.11 (-0.12--0.09) |
| Georgia | 1034062 (921407-1151218) | 18038.82 (16116-19980.02) | 314169 (284921-350473) | 5497.06 (4987.59-6127.01) | 1737 (703-3558) | 30.4 (12.36-62.25) | 834699 (742352-931658) | 18039.38 (16109.92-19985.35) | 253432 (230433-283605) | 5498.37 (4983.78-6126.86) | 1377 (561-2795) | 30.29 (12.3-61.91) | 0 (0-0) | 0 (0-0) | -0.01 (-0.01--0.01) |
| Germany | 22938632 (20426004-25562256) | 23621.1 (21131.97-26179.72) | 7598595 (6886109-8428405) | 7839.4 (7077.14-8685.34) | 41560 (17010-85528) | 43.33 (17.68-88.6) | 29157995 (25807614-32759575) | 23351.18 (20901.38-25852.5) | 9772584 (8852733-10909113) | 7755.98 (6987.26-8605.95) | 52716 (21675-109586) | 42.81 (17.64-87.76) | -0.03 (-0.04--0.03) | -0.03 (-0.04--0.02) | -0.03 (-0.04--0.03) |
| Ghana | 3602209 (3042754-4194233) | 25870.07 (22880.54-29034.46) | 1326278 (1240837-1415201) | 8661.59 (8090.29-9347.5) | 7502 (3080-15294) | 48.33 (19.86-98.26) | 7951132 (6886344-9033215) | 25513.31 (22778.95-28321.67) | 2746681 (2560075-2952858) | 8309.9 (7729.12-8922.52) | 15540 (6429-32035) | 46.45 (19.25-94.85) | -0.04 (-0.05--0.02) | -0.09 (-0.11--0.07) | -0.08 (-0.1--0.06) |
| Greece | 2831618 (2520785-3161962) | 23443.61 (20987.8-25938) | 939412 (850491-1048360) | 7781.27 (7013.19-8625.25) | 5163 (2122-10728) | 43.06 (17.63-88.53) | 3568146 (3169758-4028583) | 23312.86 (20866.78-25796.15) | 1198928 (1087688-1336738) | 7739.28 (6972.61-8582.87) | 6487 (2672-13426) | 42.82 (17.6-88.16) | -0.02 (-0.02--0.02) | -0.02 (-0.02--0.01) | -0.02 (-0.02--0.01) |
| Greenland | 4220 (3756-4724) | 9152.44 (8256.39-10170.24) | 1242 (1103-1393) | 2675.35 (2416.55-2986.14) | 7 (3-14) | 14.71 (6.03-30.17) | 5231 (4639-5867) | 8976.41 (8099.01-9980.71) | 1498 (1353-1676) | 2593.98 (2341.31-2900.64) | 8 (3-17) | 14.27 (5.78-29.58) | -0.06 (-0.07--0.05) | -0.1 (-0.11--0.09) | -0.09 (-0.1--0.09) |
| Grenada | 21080 (18972-23447) | 25732.56 (23022.55-28702.63) | 7009 (6348-7816) | 8486.96 (7682.95-9425.29) | 39 (16-80) | 46.95 (19.51-96.71) | 26985 (24007-30210) | 25611.53 (22901.25-28521.25) | 8848 (7987-9851) | 8443.13 (7645.36-9395.64) | 49 (20-102) | 46.55 (19.22-96.79) | -0.01 (-0.01-0) | -0.01 (-0.01-0) | -0.02 (-0.02--0.01) |
| Guam | 22978 (20494-25846) | 18811.83 (16910.44-20901.04) | 7204 (6330-8127) | 5845.2 (5203.49-6533.97) | 41 (16-85) | 32.67 (13.18-67.58) | 32377 (29115-36045) | 18761.55 (16860.78-20852.92) | 10015 (8981-11189) | 5833.59 (5194.89-6513.05) | 56 (23-115) | 32.55 (13.21-67.39) | 0 (-0.01-0) | 0 (-0.01-0.01) | 0 (-0.01-0.01) |
| Guatemala | 1218686 (1089708-1355068) | 18703.85 (16803.67-20680.96) | 375624 (336348-425325) | 5613.59 (5084.73-6244.13) | 2109 (860-4420) | 30.86 (12.66-63.58) | 2562830 (2303084-2852127) | 18351.32 (16484.76-20294.2) | 770256 (692387-858783) | 5471.33 (4945.07-6066.74) | 4271 (1759-8791) | 30.03 (12.36-61.99) | -0.06 (-0.07--0.05) | -0.08 (-0.09--0.07) | -0.08 (-0.09--0.07) |
| Guinea | 2074877 (1803942-2382428) | 36760.28 (32664.22-40968.95) | 914727 (757221-1090229) | 15420.29 (13504-17473.64) | 5162 (2085-10985) | 86.04 (35.86-182.22) | 4473889 (3872715-5146731) | 36487.35 (32413.88-40627.65) | 1926577 (1617811-2261533) | 14918.31 (13085.92-16820.54) | 10905 (4455-23173) | 83.3 (34.39-175.22) | -0.04 (-0.05--0.02) | -0.1 (-0.11--0.08) | -0.09 (-0.11--0.08) |
| Guinea-Bissau | 345015 (297703-398543) | 37183.96 (33216.49-41520.03) | 161886 (131163-196002) | 16045.77 (13959.55-18150.59) | 916 (368-1952) | 89.4 (36.41-187.88) | 683890 (592719-790212) | 36878.17 (32852.92-41342.53) | 303357 (250888-359749) | 15360.19 (13490.32-17250.74) | 1720 (704-3659) | 85.78 (35.53-179.99) | -0.05 (-0.06--0.03) | -0.16 (-0.18--0.14) | -0.15 (-0.17--0.14) |
| Guyana | 165128 (146880-184599) | 26007.13 (23333.28-28942.76) | 54800 (48560-62004) | 8637.09 (7822.2-9569.63) | 306 (126-637) | 47.46 (19.65-97.85) | 184014 (164435-205335) | 25860.39 (23186-28806.99) | 60767 (54762-67830) | 8586.8 (7776.95-9523.27) | 335 (138-698) | 47.07 (19.55-97.38) | -0.01 (-0.02--0.01) | -0.01 (-0.01--0.01) | -0.01 (-0.02--0.01) |
| Haiti | 1356634 (1210821-1513834) | 26225.08 (23477.82-29129.1) | 454275 (405317-512794) | 8762.32 (7924.94-9713.17) | 2535 (1041-5250) | 48.17 (19.95-99.47) | 2805608 (2520726-3131081) | 26013.64 (23353.4-28914.03) | 932996 (832209-1051115) | 8683.14 (7867.38-9637.84) | 5195 (2147-10901) | 47.7 (19.79-98.91) | -0.03 (-0.03--0.02) | -0.03 (-0.03--0.03) | -0.03 (-0.03--0.03) |
| Honduras | 691754 (619556-768544) | 18642.46 (16744.49-20632.86) | 212220 (189210-238460) | 5582.35 (5065.01-6189.13) | 1194 (487-2475) | 30.85 (12.61-63.44) | 1592118 (1431664-1771407) | 18437.73 (16559.04-20369.93) | 480999 (431673-536795) | 5516.79 (5000.27-6113.49) | 2682 (1103-5560) | 30.37 (12.44-62.55) | -0.03 (-0.04--0.03) | -0.04 (-0.04--0.03) | -0.04 (-0.05--0.04) |
| Hungary | 2174619 (1931539-2421883) | 18030.67 (16099.9-19978.1) | 658660 (597805-737262) | 5495.28 (4981.22-6123.29) | 3578 (1462-7268) | 30.05 (12.25-61.27) | 2483515 (2189805-2781734) | 18012.56 (16076.98-19962.12) | 751867 (680377-843599) | 5488.84 (4974.96-6115.54) | 4057 (1653-8227) | 30.19 (12.24-61.26) | 0 (0-0) | 0 (0-0) | 0.02 (0.02-0.02) |
| Iceland | 62743 (56061-69554) | 23470.57 (21014.93-25981.97) | 20891 (18843-23132) | 7793 (7025.51-8637.96) | 115 (47-238) | 43.17 (17.76-88.89) | 101085 (90192-112880) | 23289.65 (20840.37-25783.25) | 33821 (30746-37712) | 7735.62 (6967.91-8583.48) | 185 (76-385) | 42.89 (17.52-88.28) | -0.02 (-0.03--0.02) | -0.02 (-0.03--0.02) | -0.02 (-0.02--0.01) |
| India | 161054541 (139765978-182192618) | 20501.31 (18224.05-22761.99) | 56022068 (47705458-65483728) | 6853.21 (6021.69-7753.46) | 314340 (127202-671810) | 37.88 (15.64-78.99) | 265162118 (235600132-294116264) | 19871.63 (17699.37-21973.88) | 87230762 (77093668-98204047) | 6510.28 (5751.92-7304.64) | 485519 (197480-994783) | 36.05 (14.74-74.28) | -0.11 (-0.12--0.11) | -0.19 (-0.2--0.18) | -0.18 (-0.19--0.17) |
| Indonesia | 47346548 (41713215-53168071) | 28594.02 (25454.61-31730.35) | 18771931 (16513231-21188640) | 11172.43 (9960.63-12510.94) | 105930 (43217-221227) | 62.45 (25.23-128.36) | 78845408 (69908331-88499425) | 28436.94 (25295.18-31552.13) | 31456817 (27591739-35597049) | 11127.15 (9908.63-12470.5) | 176817 (71497-364358) | 62.29 (25.18-127.93) | -0.02 (-0.02--0.02) | -0.01 (-0.02--0.01) | -0.01 (-0.01-0) |
| Iran (Islamic Republic of) | 4671405 (4190954-5211788) | 11557.24 (10435.06-12734.36) | 1444592 (1291359-1607138) | 3505.71 (3181.73-3869.36) | 8080 (3281-16500) | 19.2 (7.9-38.71) | 9297241 (8349412-10277825) | 11248.35 (10126.58-12410.32) | 2806933 (2533131-3120342) | 3395.84 (3078.16-3749.45) | 15420 (6359-31276) | 18.55 (7.62-37.55) | -0.08 (-0.09--0.08) | -0.1 (-0.1--0.09) | -0.1 (-0.11--0.1) |
| Iraq | 1402487 (1258758-1555912) | 10955.65 (9806.92-12126.77) | 415685 (375908-464613) | 3224.44 (2935.07-3558.79) | 2307 (935-4717) | 17.58 (7.19-35.64) | 3466395 (3076234-3850931) | 10807.61 (9654.41-11982.8) | 1022828 (922143-1146947) | 3177.82 (2885.55-3510.48) | 5645 (2294-11494) | 17.23 (7.07-35.01) | -0.05 (-0.06--0.05) | -0.06 (-0.06--0.05) | -0.07 (-0.08--0.07) |
| Ireland | 885292 (792218-985812) | 23535.32 (21065.08-26044.21) | 294387 (265853-326990) | 7821.02 (7055.31-8661.24) | 1626 (667-3333) | 43.27 (17.68-88.47) | 1401130 (1247675-1565498) | 23282.06 (20826.37-25768.32) | 466833 (424304-520666) | 7731.99 (6963.88-8574.57) | 2557 (1052-5305) | 42.79 (17.59-87.99) | -0.04 (-0.04--0.04) | -0.04 (-0.04--0.04) | -0.04 (-0.04--0.04) |
| Israel | 1427920 (1278326-1595537) | 29276.45 (26244.19-32712.3) | 606086 (570384-649941) | 12454.09 (11753.62-13279.04) | 3374 (1393-7007) | 69.26 (28.53-143.62) | 3020321 (2700629-3377351) | 29037.7 (26000.4-32511.66) | 1288332 (1214671-1376649) | 12362.56 (11679.17-13199.03) | 7121 (2951-14747) | 68.73 (28.42-142.38) | -0.03 (-0.03--0.02) | -0.02 (-0.03--0.02) | -0.02 (-0.03--0.02) |
| Italy | 16584219 (14704569-18485529) | 24301.79 (21671.29-26963.82) | 5421464 (4888851-6064852) | 7956.59 (7174.84-8837.46) | 29580 (12265-61414) | 43.84 (18.02-90.09) | 21906000 (19286296-24684313) | 24074.75 (21448.88-26735.33) | 7236858 (6514598-8116550) | 7880.52 (7096.76-8746.63) | 38996 (16151-80869) | 43.55 (17.85-89.72) | -0.03 (-0.03--0.03) | -0.03 (-0.03--0.02) | -0.02 (-0.02--0.01) |
| Jamaica | 552553 (494979-615654) | 25564.41 (22862.34-28478.66) | 182700 (164497-203791) | 8416.17 (7634.59-9336.22) | 1018 (423-2105) | 46.69 (19.45-96.47) | 752760 (672744-837114) | 25493.17 (22817.01-28385.29) | 248401 (225062-277165) | 8401.08 (7607.94-9330.3) | 1370 (565-2851) | 46.5 (19.34-96.55) | -0.01 (-0.01--0.01) | -0.01 (-0.01-0) | -0.01 (-0.02--0.01) |
| Japan | 27851218 (24710232-31097552) | 19516.59 (17443.28-21785.7) | 8569807 (7745078-9539194) | 6039.97 (5453.57-6692.07) | 47240 (19115-95533) | 33.44 (13.55-67.09) | 44109283 (38559914-50206552) | 19346.69 (17293.66-21646.65) | 13757388 (12363740-15526047) | 5981.31 (5397.74-6631.8) | 74067 (30184-149065) | 33.18 (13.44-66.74) | -0.03 (-0.03--0.02) | -0.03 (-0.03--0.03) | -0.02 (-0.03--0.02) |
| Jordan | 284308 (254828-314577) | 11345.51 (10220.56-12517.06) | 86105 (77402-96356) | 3378.34 (3075.78-3735.07) | 484 (196-991) | 18.56 (7.6-37.61) | 1096088 (978903-1216372) | 11058.11 (9951.31-12237.73) | 325778 (294401-364261) | 3273.46 (2980.66-3613.35) | 1809 (730-3729) | 17.88 (7.28-36.53) | -0.09 (-0.1--0.09) | -0.11 (-0.12--0.11) | -0.13 (-0.14--0.12) |
| Kazakhstan | 2622340 (2349219-2920213) | 18042.56 (16120.76-19981.71) | 800639 (723624-894806) | 5498.81 (4994.51-6127.18) | 4442 (1791-9213) | 30.27 (12.3-62.12) | 3249712 (2915584-3604920) | 18022.11 (16096.37-19965.42) | 986963 (889430-1107823) | 5491.7 (4980.72-6121.18) | 5457 (2212-11220) | 30.21 (12.29-61.81) | -0.01 (-0.01-0) | -0.01 (-0.01-0) | -0.01 (-0.01-0) |
| Kenya | 6924589 (5985962-8078523) | 32631.02 (29142.59-36489.86) | 2516503 (2078313-3016065) | 11224.21 (9874.31-12866.95) | 14268 (5802-30763) | 62.46 (25.62-129.98) | 15523592 (13540467-17719791) | 34018.95 (30222.82-38071.52) | 5636115 (4771664-6596370) | 11871.24 (10482.49-13507.23) | 31908 (13144-67341) | 66.22 (27.11-137.86) | 0.11 (0.09-0.13) | 0.17 (0.14-0.2) | 0.18 (0.15-0.21) |
| Kiribati | 12425 (11096-13826) | 19439.3 (17463.92-21583.6) | 4021 (3537-4533) | 6172.7 (5526.05-6862.61) | 23 (9-47) | 34.21 (14-70.44) | 20917 (18738-23322) | 19292.33 (17343.66-21398.53) | 6728 (5941-7579) | 6112.95 (5461.47-6816.32) | 38 (15-79) | 33.93 (13.91-69.88) | -0.02 (-0.03--0.02) | -0.03 (-0.03--0.02) | -0.02 (-0.03--0.02) |
| Kuwait | 139974 (123845-157436) | 11239.69 (10103.69-12413.68) | 41822 (37068-47518) | 3326.27 (3022.02-3675.32) | 235 (95-481) | 18.29 (7.52-37.22) | 458231 (407858-514032) | 11010.12 (9916.87-12194.89) | 135534 (120942-152309) | 3251.69 (2959.47-3598.13) | 751 (301-1542) | 17.75 (7.25-36.19) | -0.08 (-0.08--0.07) | -0.09 (-0.09--0.08) | -0.11 (-0.12--0.11) |
| Kyrgyzstan | 675057 (601994-752448) | 18055.16 (16130.97-19997.23) | 206693 (186673-231393) | 5503.45 (4997.55-6129.68) | 1152 (468-2371) | 30.37 (12.4-62.07) | 1059817 (951533-1179738) | 18036.59 (16106.95-19986.18) | 323686 (291583-362673) | 5497.62 (4983.46-6126.66) | 1806 (731-3730) | 30.38 (12.4-62.09) | 0 (-0.01-0) | 0 (-0.01-0) | 0 (0-0.01) |
| Lao People's Democratic Republic | 966107 (860767-1093190) | 27147.18 (24415.85-30499.06) | 386686 (341628-437104) | 10773.45 (9605.35-12120.46) | 2180 (877-4561) | 60.16 (24.32-125.64) | 1834307 (1633674-2070541) | 26876.55 (24157.21-30214.83) | 739664 (651566-838370) | 10655.25 (9516.5-12023.71) | 4170 (1681-8798) | 59.65 (24.24-124.45) | -0.04 (-0.04--0.03) | -0.04 (-0.05--0.04) | -0.03 (-0.04--0.03) |
| Latvia | 545734 (485960-605938) | 18023.07 (16104.76-19961.68) | 165394 (150940-184184) | 5491.81 (4985.59-6120.13) | 902 (368-1842) | 30.16 (12.29-61.86) | 500291 (441509-560699) | 18018.09 (16092.37-19961.03) | 151562 (137493-170409) | 5490.01 (4979.37-6117.52) | 817 (333-1652) | 30.2 (12.23-61.56) | 0 (0-0) | 0 (0-0) | 0.01 (0.01-0.02) |
| Lebanon | 281329 (253862-311048) | 11236.19 (10123.01-12432.06) | 83877 (76103-92710) | 3335.95 (3040.08-3686.56) | 464 (190-944) | 18.24 (7.47-36.78) | 656562 (590005-727361) | 10960.88 (9853.47-12130.26) | 193830 (176076-214365) | 3238.03 (2953.13-3577.41) | 1051 (428-2132) | 17.62 (7.2-36) | -0.08 (-0.08--0.08) | -0.1 (-0.1--0.09) | -0.11 (-0.11--0.1) |
| Lesotho | 389417 (344848-436615) | 29231.55 (26083.13-32370.89) | 136205 (119829-155467) | 10072.55 (9059.86-11189.92) | 766 (312-1592) | 55.88 (22.69-114.24) | 485853 (429990-542552) | 29622.92 (26446.85-32798.99) | 171182 (149322-195213) | 10291.8 (9260.76-11527.33) | 955 (389-1988) | 56.61 (22.9-115.88) | 0.05 (0.03-0.06) | 0.08 (0.06-0.1) | 0.06 (0.03-0.08) |
| Liberia | 837424 (726152-959563) | 36740.65 (32819.75-41011.64) | 373875 (308699-447969) | 15459.89 (13493.64-17564.28) | 2100 (837-4481) | 85.56 (34.31-179.99) | 1740736 (1510006-1966063) | 35873.02 (31912.69-39939.43) | 723555 (612124-842288) | 14347.64 (12628.7-16166.21) | 4072 (1661-8708) | 79.47 (32.25-165.93) | -0.09 (-0.11--0.08) | -0.26 (-0.28--0.23) | -0.25 (-0.28--0.23) |
| Libya | 341210 (308643-377818) | 11311.56 (10189.61-12490.24) | 102876 (92825-113870) | 3362.27 (3058.54-3712.06) | 575 (231-1171) | 18.48 (7.49-37.47) | 688135 (616083-763767) | 11087.72 (9975.23-12272.18) | 204336 (184369-227835) | 3290.01 (2995.05-3629.27) | 1126 (457-2286) | 17.93 (7.32-36.16) | -0.06 (-0.06--0.06) | -0.07 (-0.07--0.07) | -0.09 (-0.1--0.09) |
| Lithuania | 725675 (647645-806216) | 18029.94 (16103.5-19969.02) | 220317 (200815-245541) | 5495.35 (4985.95-6123.11) | 1207 (492-2470) | 30.22 (12.33-62.03) | 728727 (643772-816627) | 18020.84 (16094.17-19964.48) | 220830 (200276-248085) | 5491.49 (4979.97-6118.83) | 1189 (486-2393) | 30.19 (12.27-61.79) | 0 (0-0) | 0 (0-0) | 0 (0-0) |
| Luxembourg | 104240 (92808-115504) | 23602.52 (21124.55-26131.98) | 34515 (31239-38268) | 7838.55 (7086.49-8680.78) | 189 (78-389) | 43.33 (17.79-88.54) | 187202 (166817-207489) | 23328.72 (20877.53-25825.95) | 62401 (56772-69635) | 7750.41 (6988.62-8587.3) | 341 (141-705) | 42.89 (17.71-87.95) | -0.04 (-0.04--0.03) | -0.04 (-0.04--0.03) | -0.03 (-0.04--0.02) |
| Madagascar | 3648654 (3208792-4126458) | 34316.42 (30813.32-37856.46) | 1433002 (1268041-1639759) | 13145.41 (11869.41-14566.3) | 8061 (3275-16431) | 72.88 (30.1-150.62) | 8418110 (7455531-9451256) | 33885.14 (30535-37337.89) | 3236943 (2878511-3650204) | 12790.48 (11524.72-14126.57) | 18283 (7506-37439) | 71.16 (29.38-146.38) | -0.04 (-0.05--0.04) | -0.08 (-0.09--0.07) | -0.06 (-0.07--0.05) |
| Malawi | 3331910 (2898615-3861829) | 36597.27 (32495.97-40894.36) | 1493695 (1232918-1785309) | 15280.91 (13364.53-17487.64) | 8426 (3438-17946) | 84.93 (34.97-177.69) | 6376474 (5556527-7304172) | 36242.44 (32363.27-40300.79) | 2734821 (2287540-3244635) | 14661.6 (12901.05-16717.97) | 15488 (6341-32872) | 81.8 (34.04-173.58) | -0.05 (-0.06--0.04) | -0.13 (-0.15--0.12) | -0.11 (-0.13--0.09) |
| Malaysia | 4211997 (3742073-4771365) | 26820.99 (24119.47-30145.64) | 1688535 (1484651-1911257) | 10613.91 (9463.67-11954.36) | 9517 (3813-20053) | 59.29 (24.02-123.82) | 8400312 (7517139-9461292) | 26566.14 (23842.11-29903.07) | 3362209 (2970690-3815705) | 10515.48 (9375.37-11873.75) | 18845 (7539-40122) | 58.74 (23.71-123.58) | -0.03 (-0.03--0.03) | -0.03 (-0.04--0.03) | -0.03 (-0.03--0.03) |
| Maldives | 48793 (43243-55179) | 26862.18 (24153.81-30146.21) | 19487 (17013-22046) | 10635.91 (9487.81-12007.29) | 110 (44-231) | 59.4 (24.15-124.44) | 137169 (121127-156077) | 26518.83 (23696.74-29845.42) | 55762 (48738-64402) | 10541.29 (9392.16-11931.98) | 314 (125-673) | 59.03 (23.89-124.73) | -0.05 (-0.06--0.04) | -0.03 (-0.05--0.02) | -0.02 (-0.03--0.01) |
| Mali | 3662633 (3199209-4236954) | 43695.93 (38964.27-49001.34) | 2182505 (1858215-2530945) | 23993.3 (21348.45-26735.86) | 12326 (4971-26084) | 133.91 (54.2-277.87) | 10099792 (8815661-11703522) | 43741.39 (39113.77-49030.62) | 6003043 (5084622-6968244) | 23696.61 (20972.72-26338.73) | 34017 (13761-72060) | 132.53 (54.3-273.61) | -0.03 (-0.04--0.01) | -0.12 (-0.14--0.09) | -0.11 (-0.13--0.08) |
| Malta | 91764 (81965-101584) | 23524.62 (21060.2-26023.8) | 30380 (27457-33589) | 7813.62 (7044.36-8658.88) | 168 (69-348) | 43.31 (17.8-89.1) | 147245 (129800-166044) | 23324.34 (20861.61-25838.6) | 49337 (44727-55162) | 7746.66 (6976.94-8588.79) | 268 (110-562) | 42.9 (17.69-88.01) | -0.03 (-0.03--0.02) | -0.03 (-0.03--0.02) | -0.03 (-0.03--0.02) |
| Marshall Islands | 7113 (6272-7958) | 19248.31 (17284.74-21374.6) | 2296 (2023-2603) | 6051.93 (5415.42-6761.88) | 13 (5-27) | 33.66 (13.7-68.99) | 9808 (8757-10970) | 19155.6 (17231.54-21292.46) | 3120 (2757-3524) | 6022.09 (5383.36-6743.2) | 17 (7-36) | 33.37 (13.52-68.05) | -0.02 (-0.02--0.01) | -0.02 (-0.03--0.01) | -0.03 (-0.04--0.02) |
| Mauritania | 705040 (612342-811866) | 36916.51 (32870.82-41180.08) | 315413 (262847-374287) | 15568.23 (13553.89-17664.92) | 1784 (711-3841) | 87 (35.1-184.64) | 1429574 (1248147-1614075) | 35696.76 (31948.37-39708.63) | 587571 (502894-690898) | 14190.67 (12577.59-16070.37) | 3324 (1359-7034) | 79.34 (32.23-165.23) | -0.11 (-0.13--0.1) | -0.3 (-0.31--0.29) | -0.3 (-0.3--0.29) |
| Mauritius | 271813 (241940-307638) | 26626.22 (23904.02-29966.03) | 108940 (96008-123877) | 10523.17 (9384.96-11872.55) | 612 (244-1302) | 58.68 (23.7-122.62) | 374163 (333651-420962) | 26503.99 (23798.62-29814.03) | 147624 (130557-167204) | 10480.88 (9337.9-11827.12) | 817 (332-1713) | 58.33 (23.71-121.97) | -0.01 (-0.01--0.01) | -0.01 (-0.01-0) | -0.01 (-0.02--0.01) |
| Mexico | 12301839 (10981009-13644591) | 17617.57 (15942.82-19531.62) | 3784375 (3410213-4212310) | 5316.82 (4841.6-5853.08) | 21195 (8693-43388) | 29.25 (12.19-59.35) | 24763824 (22364841-27470397) | 19444.56 (17518.42-21611.24) | 7588938 (6890913-8430945) | 5965.33 (5425.72-6614.86) | 41863 (17035-85598) | 32.85 (13.45-66.86) | 0.21 (0.1-0.33) | 0.25 (0.11-0.39) | 0.25 (0.11-0.39) |
| Micronesia (Federated States of) | 16878 (14936-18853) | 19303.95 (17310.62-21396.53) | 5438 (4791-6122) | 6083.27 (5443.38-6804.93) | 31 (13-64) | 33.85 (13.75-69.17) | 18144 (16206-20285) | 19065.08 (17126.16-21227.3) | 5748 (5100-6479) | 5976.69 (5337.68-6680.39) | 32 (13-67) | 33.21 (13.55-68.17) | -0.04 (-0.05--0.04) | -0.06 (-0.07--0.06) | -0.07 (-0.07--0.07) |
| Monaco | 10496 (9247-11832) | 23498.93 (21024.33-25989.6) | 3504 (3176-3936) | 7800.52 (7035.79-8642.63) | 19 (8-40) | 43.32 (17.75-89.25) | 13718 (12085-15651) | 23305.93 (20853.27-25797.4) | 4614 (4183-5178) | 7741.77 (6969.24-8581.47) | 25 (10-52) | 42.91 (17.73-88.43) | -0.03 (-0.03--0.03) | -0.03 (-0.03--0.02) | -0.03 (-0.03--0.03) |
| Mongolia | 297088 (264246-334354) | 18108.19 (16171.93-20064.83) | 91405 (81819-103320) | 5525.59 (5015.73-6152.38) | 512 (208-1049) | 30.48 (12.4-62.09) | 511329 (459677-570367) | 18044.28 (16110.98-19991.89) | 155946 (140405-174702) | 5501.8 (4989.88-6128.27) | 869 (351-1774) | 30.3 (12.28-61.76) | -0.01 (-0.01--0.01) | -0.02 (-0.02--0.02) | -0.02 (-0.02--0.02) |
| Montenegro | 112092 (100102-124473) | 18034.63 (16090.97-19981.69) | 34131 (30872-38142) | 5497.3 (4986.09-6122.72) | 189 (77-386) | 30.34 (12.38-61.9) | 136036 (120354-152285) | 18024.48 (16081.39-19972.63) | 41109 (37146-46250) | 5494.21 (4982.23-6121.99) | 224 (92-457) | 30.24 (12.32-61.8) | 0 (0-0) | 0 (0-0) | 0 (-0.01-0) |
| Morocco | 2247572 (2030175-2473474) | 11412.3 (10277.84-12620.84) | 679575 (613999-752290) | 3411.54 (3114.87-3771.07) | 3786 (1541-7746) | 18.74 (7.69-38.11) | 3915038 (3511009-4334329) | 11123.54 (10017.19-12308.31) | 1163066 (1059711-1288960) | 3306.45 (3012.07-3650.1) | 6376 (2629-12956) | 18.02 (7.42-36.37) | -0.08 (-0.08--0.08) | -0.1 (-0.1--0.1) | -0.12 (-0.12--0.12) |
| Mozambique | 4401731 (3815110-5011783) | 35637.94 (31980.81-39724.1) | 1826157 (1656343-2022638) | 14119.29 (12898.87-15502.7) | 10255 (4238-21210) | 78.02 (32.23-162.63) | 10015914 (8657667-11459926) | 35600.47 (32019.7-39616.58) | 4115301 (3687682-4567197) | 13934.15 (12709.07-15255.6) | 23212 (9557-48354) | 77.15 (31.89-162.04) | 0 (-0.01-0.02) | 0.01 (-0.02-0.03) | 0.03 (0-0.06) |
| Myanmar | 9730121 (8626076-11014021) | 27019.09 (24305.42-30325.1) | 3912657 (3441208-4431325) | 10707.98 (9556.11-12072.97) | 22000 (8793-46479) | 59.66 (24.1-124.22) | 14731280 (13202375-16629402) | 26728.11 (24026.16-30064.49) | 5902709 (5228813-6693432) | 10573.6 (9438.98-11917.81) | 33101 (13249-69624) | 59.07 (23.73-123.57) | -0.04 (-0.04--0.04) | -0.05 (-0.05--0.05) | -0.04 (-0.04--0.03) |
| Namibia | 348001 (308237-391014) | 29468.79 (26406.06-32588.85) | 122766 (107008-140365) | 10221.89 (9213.66-11321.48) | 692 (283-1446) | 56.72 (23.07-116.2) | 614568 (548940-687369) | 29026.39 (25916.56-32118.31) | 212504 (188359-241143) | 9953.92 (8954.79-11078.44) | 1190 (484-2492) | 55.04 (22.35-113.22) | -0.06 (-0.07--0.05) | -0.09 (-0.11--0.08) | -0.1 (-0.11--0.09) |
| Nauru | 1660 (1481-1852) | 19272.58 (17311.02-21417.81) | 531 (468-601) | 6056.94 (5412.8-6763.65) | 3 (1-6) | 33.73 (13.66-69.3) | 1841 (1638-2059) | 19070.45 (17145.58-21218.57) | 587 (517-662) | 5983.96 (5367.03-6671.64) | 3 (1-7) | 33.2 (13.56-68.4) | -0.03 (-0.04--0.02) | -0.04 (-0.05--0.02) | -0.04 (-0.06--0.03) |
| Nepal | 3329806 (2925547-3798780) | 19328.25 (17294.86-21396.47) | 1149738 (977112-1347327) | 6355.07 (5609.29-7189.24) | 6470 (2588-13809) | 35.15 (14.34-72.85) | 5364144 (4801984-5962107) | 18753.77 (16871.91-20737.86) | 1738172 (1537434-1966679) | 6003.79 (5342.59-6725.99) | 9705 (3998-19786) | 33.25 (13.71-67.8) | -0.1 (-0.1--0.09) | -0.18 (-0.2--0.17) | -0.18 (-0.19--0.16) |
| Netherlands | 3971816 (3537502-4404241) | 23510.82 (21042.28-26030.63) | 1317409 (1193937-1459167) | 7799.87 (7037.95-8646.99) | 7258 (2990-14963) | 43.23 (17.74-88.72) | 5485955 (4845090-6185715) | 23297.6 (20849.2-25796.26) | 1834115 (1666890-2054140) | 7737.46 (6969.69-8578.26) | 9974 (4097-20785) | 42.81 (17.55-87.8) | -0.03 (-0.03--0.03) | -0.03 (-0.03--0.03) | -0.03 (-0.04--0.03) |
| New Zealand | 920728 (824005-1030654) | 25382.44 (22754.1-28310.84) | 321366 (289885-355981) | 8828.82 (8022.99-9751.15) | 1754 (731-3577) | 48.3 (19.98-98.2) | 1616154 (1440268-1807920) | 25171.76 (22558.74-28102.92) | 575559 (522492-637437) | 8764.2 (7946.31-9695.16) | 3123 (1302-6371) | 48.12 (19.98-98.22) | -0.03 (-0.03--0.02) | -0.02 (-0.03--0.02) | -0.02 (-0.02--0.01) |
| Nicaragua | 551184 (494300-615479) | 18391.35 (16523.55-20326.25) | 167414 (149530-188939) | 5463.37 (4942.59-6057.65) | 941 (384-1959) | 30.14 (12.31-61.93) | 1085195 (974140-1207211) | 18156.4 (16302.52-20026.16) | 322895 (292068-359232) | 5379.71 (4871.5-5968.7) | 1793 (733-3694) | 29.62 (12.03-60.81) | -0.05 (-0.05--0.05) | -0.06 (-0.07--0.06) | -0.07 (-0.07--0.06) |
| Niger | 3042413 (2641387-3512202) | 40894.56 (37108.99-45534.99) | 1471814 (1341699-1601440) | 18976.28 (17478.24-20659.28) | 8321 (3329-17356) | 105.67 (43.23-220.3) | 9098693 (7999677-10336439) | 39968.98 (36454.18-44207.33) | 4131453 (3762671-4539527) | 17837.67 (16300.87-19503.22) | 23420 (9604-49340) | 99.54 (40.64-207.98) | -0.11 (-0.13--0.09) | -0.25 (-0.28--0.22) | -0.24 (-0.28--0.21) |
| Nigeria | 37675384 (33229634-42451802) | 44489.69 (39660.43-49317.86) | 17595350 (15109347-20275117) | 20696.51 (18473.23-23279.05) | 98883 (40273-208790) | 114.94 (47.27-240.66) | 95963470 (83963374-108223361) | 44547.86 (39673.25-49362.51) | 45466757 (39022952-52447926) | 20826.82 (18579.08-23380.75) | 256949 (104358-544387) | 116.15 (47.73-242.55) | -0.04 (-0.06--0.02) | -0.05 (-0.08--0.01) | -0.03 (-0.06-0) |
| Niue | 430 (385-478) | 19011.12 (17086.21-21116.2) | 135 (121-150) | 5935.21 (5312.72-6621.08) | 1 (0-2) | 33.06 (13.42-67.81) | 336 (302-375) | 18848.8 (16925.03-20955.79) | 104 (93-117) | 5874.52 (5250.08-6557) | 1 (0-1) | 32.61 (13.2-67.05) | -0.03 (-0.03--0.03) | -0.03 (-0.03--0.03) | -0.04 (-0.04--0.04) |
| North Macedonia | 345695 (309782-383685) | 18067.62 (16136.29-20016.89) | 105364 (95108-117892) | 5510.8 (5009.37-6136.8) | 582 (235-1202) | 30.32 (12.32-61.88) | 468130 (413755-524469) | 18044.79 (16101.9-19995.47) | 141246 (127257-158710) | 5501.8 (4995.8-6126.88) | 772 (315-1572) | 30.23 (12.3-61.98) | 0 (0-0) | -0.01 (-0.01-0) | 0 (-0.01-0) |
| Northern Mariana Islands | 7554 (6680-8569) | 18923.74 (16964.15-21018.38) | 2377 (2069-2706) | 5891.14 (5262.67-6595.52) | 13 (5-28) | 32.88 (13.38-67.34) | 9338 (8373-10469) | 18798.86 (16899.25-20885.44) | 2906 (2591-3273) | 5848.29 (5221.43-6525.03) | 16 (7-34) | 32.58 (13.16-67.44) | -0.01 (-0.02-0) | -0.01 (-0.02-0) | -0.01 (-0.02-0) |
| Norway | 1261963 (1122839-1406575) | 24375.67 (21741.45-27043.85) | 414497 (374327-462145) | 7988.04 (7199.91-8869.16) | 2256 (938-4676) | 44.04 (18.09-90.46) | 1705978 (1516421-1901489) | 24121.95 (21496.09-26775.19) | 562208 (507493-627040) | 7904.4 (7114.08-8770.09) | 3058 (1269-6338) | 43.7 (17.92-90.03) | -0.03 (-0.04--0.03) | -0.03 (-0.04--0.03) | -0.02 (-0.03--0.02) |
| Oman | 156634 (139796-174692) | 11344.09 (10222.28-12527.87) | 47221 (42251-53060) | 3371.19 (3061.75-3728.8) | 265 (107-538) | 18.44 (7.53-37.35) | 411630 (363056-463022) | 11082.82 (9947.42-12265.5) | 122538 (108325-139064) | 3279.82 (2977.59-3618.38) | 686 (275-1418) | 17.92 (7.29-36.52) | -0.07 (-0.07--0.06) | -0.08 (-0.09--0.07) | -0.08 (-0.09--0.08) |
| Pakistan | 20080062 (17441849-22780254) | 19914.79 (17886.93-22004.65) | 6795585 (5818424-7949149) | 6489.79 (5751.07-7284.95) | 38213 (15409-81384) | 35.97 (14.56-75.41) | 41337539 (36424661-46462323) | 19771.19 (17760.17-21904.11) | 13830281 (11894115-15936194) | 6435.18 (5676.22-7215.77) | 77693 (31207-161655) | 35.63 (14.31-73.48) | -0.04 (-0.05--0.03) | -0.05 (-0.07--0.03) | -0.05 (-0.07--0.03) |
| Palau | 2646 (2361-2966) | 19046.52 (17132.24-21168.55) | 835 (736-942) | 5949.77 (5319-6647.41) | 5 (2-10) | 33.12 (13.46-68.14) | 3674 (3286-4125) | 18958.89 (17050.06-21074.45) | 1141 (1010-1287) | 5916.44 (5290.11-6616.56) | 6 (3-13) | 32.83 (13.32-67.11) | -0.01 (-0.01--0.01) | -0.01 (-0.01--0.01) | -0.02 (-0.02--0.02) |
| Palestine | 159175 (143486-176107) | 11222.78 (10117.52-12415.9) | 47866 (43191-53218) | 3330.84 (3036.21-3678.39) | 268 (109-547) | 18.26 (7.48-37.03) | 418615 (373436-466339) | 11017.49 (9902.17-12191.82) | 124973 (112552-139619) | 3261.74 (2967.8-3600.64) | 695 (282-1425) | 17.8 (7.27-36) | -0.06 (-0.06--0.06) | -0.07 (-0.07--0.07) | -0.08 (-0.08--0.08) |
| Panama | 377415 (339788-419918) | 18403.45 (16528.87-20328.89) | 113234 (101936-126128) | 5466.48 (4938.15-6051.42) | 633 (259-1303) | 30.22 (12.36-62.38) | 791115 (710577-874740) | 18138.72 (16273.98-20026.15) | 234330 (211634-259789) | 5371.23 (4860.13-5962.8) | 1291 (528-2663) | 29.62 (12.11-61.11) | -0.04 (-0.04--0.04) | -0.05 (-0.05--0.04) | -0.05 (-0.06--0.05) |
| Papua New Guinea | 655269 (581859-732889) | 19084.63 (17150.97-21197.91) | 208465 (184490-235279) | 5967.54 (5326.4-6655.58) | 1172 (478-2444) | 33.06 (13.53-68.17) | 1725204 (1539294-1935481) | 19076.18 (17150.46-21190.51) | 548237 (483739-618810) | 5977.26 (5356.32-6667.33) | 3084 (1254-6345) | 33.17 (13.47-68.32) | 0 (0-0) | 0.01 (0.01-0.01) | 0.02 (0.02-0.02) |
| Paraguay | 862459 (771915-961712) | 25686.08 (22996.79-28584.06) | 284507 (254467-318980) | 8471.6 (7672.41-9394.35) | 1590 (661-3292) | 46.77 (19.48-96.89) | 1710677 (1527260-1907455) | 25528.86 (22826.45-28404.02) | 562775 (504761-627767) | 8420.63 (7633.2-9356.12) | 3115 (1283-6493) | 46.35 (19.23-95.79) | -0.02 (-0.02--0.02) | -0.02 (-0.02--0.02) | -0.02 (-0.03--0.02) |
| Peru | 5870920 (5196062-6564259) | 31490.07 (28212.01-35087.48) | 2338992 (2056889-2639067) | 12498.06 (11228.54-13850.2) | 13119 (5360-27635) | 69.36 (28.57-145.33) | 11026675 (9836150-12278894) | 30964.08 (27667.34-34596.83) | 4371839 (3907548-4857577) | 12292.33 (11010.09-13653.13) | 24299 (9972-51033) | 68.22 (28.14-143.03) | -0.06 (-0.06--0.05) | -0.06 (-0.06--0.05) | -0.05 (-0.05--0.05) |
| Philippines | 15315017 (13522260-17151211) | 27986.96 (25108.99-31177.84) | 5927486 (5195973-6760959) | 10717.89 (9532.71-12116.21) | 33382 (13520-69860) | 59.67 (24.33-123.8) | 29960578 (26709093-33573893) | 27916.12 (25061.31-31098.36) | 11640996 (10252970-13255974) | 10699.92 (9509.57-12097.99) | 65367 (26444-136845) | 59.72 (24.28-124.01) | -0.01 (-0.01-0) | 0 (-0.01-0) | 0.01 (0-0.01) |
| Poland | 7397943 (6603032-8254632) | 18428.48 (16490.48-20466.16) | 2265705 (2062491-2527892) | 5662.1 (5148.65-6294.88) | 12384 (5047-25555) | 31 (12.69-63.89) | 9618610 (8492551-10772250) | 18403.05 (16468.89-20436.81) | 2934423 (2665122-3309186) | 5653.06 (5138.99-6285.75) | 15852 (6475-32115) | 31.06 (12.72-64.02) | 0 (0-0) | 0 (0-0) | 0.01 (0.01-0.02) |
| Portugal | 2687555 (2392047-3001942) | 23571.1 (21092.36-26073.67) | 891482 (804942-994855) | 7834.28 (7073.06-8673.69) | 4899 (2010-10150) | 43.27 (17.81-88.34) | 3668294 (3242357-4145040) | 23290.16 (20844.89-25780.79) | 1228917 (1114468-1373063) | 7725.94 (6955.39-8567.72) | 6649 (2739-13878) | 42.69 (17.58-87.76) | -0.04 (-0.04--0.04) | -0.04 (-0.05--0.04) | -0.04 (-0.04--0.03) |
| Puerto Rico | 922580 (825970-1031402) | 25491.17 (22784.24-28392.94) | 303491 (274226-338065) | 8384.95 (7598.55-9318.16) | 1681 (695-3498) | 46.41 (19.2-96.36) | 1197602 (1063508-1352276) | 25251.01 (22552.44-28170.77) | 399583 (360725-446279) | 8294.21 (7499.07-9228.9) | 2158 (889-4456) | 45.77 (18.94-94.91) | -0.03 (-0.04--0.03) | -0.04 (-0.04--0.04) | -0.05 (-0.05--0.04) |
| Qatar | 36895 (32264-41842) | 11428.31 (10246.92-12590.33) | 11065 (9668-12799) | 3396.97 (3088.59-3749.89) | 62 (25-126) | 18.62 (7.65-37.68) | 267872 (232064-305136) | 11102.55 (9921.67-12266.7) | 79403 (68790-92527) | 3279.48 (2979.9-3622.53) | 445 (177-910) | 17.85 (7.33-36.22) | -0.09 (-0.09--0.09) | -0.12 (-0.12--0.11) | -0.13 (-0.14--0.13) |
| Republic of Korea | 7381090 (6599888-8217678) | 19294.91 (17304.08-21350.02) | 2266780 (2028373-2543205) | 5939.77 (5379.77-6550.63) | 12665 (5134-26055) | 32.75 (13.42-66.7) | 12996496 (11563248-14535022) | 18950.69 (16927.71-21010.42) | 3956153 (3557922-4398608) | 5794.65 (5242.82-6402.37) | 21569 (8883-44407) | 32 (13.21-65.24) | -0.06 (-0.06--0.05) | -0.08 (-0.09--0.07) | -0.07 (-0.08--0.06) |
| Republic of Moldova | 775238 (692532-863665) | 18041.99 (16116.41-19986.16) | 235679 (212719-263538) | 5500.18 (4988.48-6129.88) | 1302 (528-2680) | 30.26 (12.32-62.07) | 830320 (737304-926632) | 18028.16 (16097.4-19971.98) | 251457 (228211-281442) | 5494.98 (4983.15-6120.38) | 1370 (559-2774) | 30.28 (12.37-61.54) | 0 (0-0) | 0 (0-0) | 0.01 (0-0.01) |
| Romania | 4449730 (3953700-4957962) | 18053.15 (16111.49-20001.28) | 1350148 (1224104-1509620) | 5505.22 (4996.61-6130.61) | 7421 (3013-15277) | 30.28 (12.33-61.81) | 4809434 (4250251-5373698) | 18024.05 (16081.95-19977.84) | 1456287 (1318969-1632391) | 5493.7 (4982.02-6118.43) | 7909 (3222-16125) | 30.32 (12.35-62.19) | 0 (-0.01-0) | -0.01 (-0.01--0.01) | 0.01 (0.01-0.01) |
| Russian Federation | 29489783 (26278196-32901378) | 18421.2 (16467.39-20484.53) | 9015265 (8197553-10071712) | 5658.06 (5146.02-6292.47) | 49356 (20119-101896) | 31.03 (12.73-64) | 33793482 (29866735-37810539) | 18409.51 (16463.24-20452.93) | 10306776 (9381267-11588538) | 5655.11 (5141.84-6288.03) | 55806 (22685-113388) | 31.01 (12.73-63.96) | 0 (-0.01-0) | 0 (-0.01-0) | 0 (0-0) |
| Rwanda | 2830017 (2442741-3288449) | 39757.05 (35441.86-44602.22) | 1691286 (1481938-1862094) | 20393.02 (18446.82-22276.86) | 9600 (3890-19817) | 114.36 (46.36-233.62) | 4722206 (4084495-5479141) | 38271.55 (34035.8-43380.35) | 2217571 (2032542-2410457) | 16830.94 (15513.85-18257.4) | 12563 (5086-25781) | 94.25 (38.36-193.97) | -0.19 (-0.2--0.17) | -0.76 (-0.81--0.71) | -0.76 (-0.81--0.71) |
| Saint Kitts and Nevis | 10372 (9226-11562) | 25990.25 (23304.29-28937.84) | 3457 (3120-3869) | 8616.68 (7791.85-9533.29) | 19 (8-39) | 47.6 (19.73-98.53) | 15765 (14066-17724) | 25618 (22929.2-28515.28) | 5145 (4624-5780) | 8454.46 (7661.63-9389.91) | 28 (12-59) | 46.63 (19.28-96.83) | -0.04 (-0.05--0.04) | -0.06 (-0.07--0.05) | -0.06 (-0.07--0.05) |
| Saint Lucia | 30432 (27217-33983) | 25725.98 (23003.23-28660.21) | 10056 (8991-11274) | 8481.9 (7685.7-9418.91) | 56 (23-117) | 46.79 (19.37-96.81) | 51173 (45784-57330) | 25531.99 (22836.41-28429.76) | 16821 (15217-18754) | 8417.41 (7636.31-9349.91) | 92 (38-191) | 46.37 (19.19-95.77) | -0.02 (-0.03--0.02) | -0.02 (-0.03--0.02) | -0.02 (-0.03--0.02) |
| Saint Vincent and the Grenadines | 24537 (21852-27478) | 25693.17 (22976.47-28606.06) | 8120 (7242-9080) | 8468.25 (7667.25-9397.32) | 45 (19-94) | 46.85 (19.43-96.22) | 31698 (28344-35415) | 25618.8 (22929.09-28551.15) | 10447 (9462-11662) | 8461.85 (7676.1-9391.6) | 57 (24-119) | 46.64 (19.26-96.49) | -0.01 (-0.01--0.01) | 0 (-0.01-0) | -0.01 (-0.02--0.01) |
| Samoa | 27422 (24286-30677) | 19111.53 (17172.82-21218.23) | 8737 (7732-9852) | 5979.54 (5346.33-6679.31) | 49 (20-103) | 33.29 (13.53-68.34) | 36338 (32492-40420) | 18926.44 (17003.39-21014.28) | 11456 (10180-12834) | 5907.66 (5269.27-6597.88) | 64 (26-133) | 32.81 (13.34-67.32) | -0.03 (-0.03--0.02) | -0.03 (-0.04--0.03) | -0.04 (-0.05--0.04) |
| San Marino | 6582 (5874-7336) | 23466.22 (21008.99-25975.68) | 2191 (1984-2432) | 7789.65 (7024.12-8631.76) | 12 (5-25) | 43.16 (17.78-88.25) | 11247 (10007-12640) | 23279.87 (20834.74-25758.59) | 3791 (3444-4235) | 7733.26 (6963.95-8579.39) | 21 (8-43) | 42.8 (17.64-87.71) | -0.02 (-0.03--0.02) | -0.02 (-0.03--0.02) | -0.03 (-0.03--0.02) |
| Sao Tome and Principe | 41263 (35682-47507) | 36355.5 (32390.38-40645.79) | 17907 (14972-21134) | 14937.43 (13126.83-16901.47) | 101 (40-217) | 83.58 (33.65-175.52) | 69853 (61224-78642) | 35656.04 (31756.78-39590.69) | 28438 (24500-32791) | 14117.04 (12462.36-15879.85) | 161 (66-337) | 78.89 (32.29-165.52) | -0.07 (-0.08--0.06) | -0.19 (-0.2--0.17) | -0.19 (-0.2--0.18) |
| Saudi Arabia | 1276956 (1139428-1420151) | 11492.35 (10355.65-12697.39) | 389046 (349821-434424) | 3436.73 (3123.95-3792.38) | 2180 (884-4468) | 18.75 (7.7-38.24) | 3447226 (3053896-3872767) | 11149.76 (10026.52-12338.48) | 1025007 (910430-1158918) | 3305.96 (3011.8-3653.29) | 5711 (2303-11610) | 17.99 (7.35-36.24) | -0.1 (-0.1--0.1) | -0.13 (-0.13--0.13) | -0.14 (-0.14--0.14) |
| Senegal | 2550929 (2203044-2935857) | 36480.07 (32426.37-40659.08) | 1124652 (931062-1344289) | 15133.52 (13260.41-17270.61) | 6345 (2568-13486) | 84.2 (34.64-177.38) | 5204391 (4528735-5923107) | 36092 (31973.53-40174.18) | 2180866 (1848785-2518183) | 14565.42 (12801.21-16473.19) | 12318 (5041-26896) | 81.22 (33.39-172.5) | -0.04 (-0.05--0.02) | -0.11 (-0.12--0.09) | -0.1 (-0.11--0.08) |
| Serbia | 1806453 (1610711-2013567) | 18050.04 (16102.61-20000.49) | 547835 (493816-610878) | 5502.97 (4992.45-6129.24) | 3021 (1209-6158) | 30.33 (12.31-61.92) | 2177295 (1927956-2439071) | 18036.29 (16092.36-19984.98) | 659632 (597132-741983) | 5498.47 (4990.1-6122.96) | 3577 (1456-7269) | 30.27 (12.22-61.91) | 0 (0-0) | 0 (0-0) | 0 (0-0) |
| Seychelles | 18340 (16257-20788) | 26790.54 (24072.63-30151.41) | 7285 (6438-8230) | 10603.25 (9465.53-11955.62) | 41 (16-87) | 59.37 (24-124.29) | 29308 (26142-32897) | 26626.1 (23885.67-29981.57) | 11709 (10348-13308) | 10550.29 (9409.3-11915.63) | 65 (26-137) | 58.92 (23.79-123.85) | -0.02 (-0.02--0.02) | -0.01 (-0.02--0.01) | -0.02 (-0.02--0.02) |
| Sierra Leone | 1398461 (1216027-1602083) | 36558.13 (32545.53-40936.32) | 608775 (510514-715425) | 15204.26 (13332.84-17101.36) | 3429 (1394-7358) | 84.6 (34.94-177.2) | 2886598 (2524451-3308656) | 36228.43 (32442-40432.26) | 1212081 (1022336-1423065) | 14627.63 (12843.66-16522.22) | 6847 (2800-14801) | 81.53 (33.08-172.79) | -0.04 (-0.05--0.03) | -0.12 (-0.13--0.11) | -0.11 (-0.12--0.09) |
| Singapore | 526268 (470032-586139) | 19166.68 (17167.21-21216.29) | 160643 (143516-179546) | 5883.97 (5331.96-6479.51) | 899 (367-1876) | 32.64 (13.43-67.11) | 1312912 (1170801-1465798) | 18939.79 (16905.22-21011.81) | 399519 (360182-442924) | 5795 (5241.69-6397.9) | 2207 (911-4570) | 32.22 (13.27-65.96) | -0.04 (-0.04--0.03) | -0.05 (-0.05--0.04) | -0.04 (-0.04--0.03) |
| Slovakia | 999401 (886990-1111200) | 18034.79 (16100.27-19983.57) | 303810 (276109-339313) | 5497.19 (4983.88-6124.33) | 1665 (670-3433) | 30.17 (12.18-62.11) | 1280113 (1132300-1430790) | 18019.97 (16081.25-19975.71) | 387191 (350446-433702) | 5491.47 (4977.6-6117.81) | 2100 (856-4262) | 30.19 (12.23-61.42) | 0 (0-0) | 0 (0-0) | 0.01 (0-0.01) |
| Slovenia | 393519 (350803-437025) | 18019.79 (16085.15-19959.35) | 119463 (108563-133224) | 5491.31 (4984.31-6114.03) | 652 (264-1333) | 30.08 (12.22-61.58) | 554872 (490539-621275) | 18031.57 (16086.04-19986.93) | 168351 (152466-188819) | 5495.67 (4987.12-6120.93) | 904 (370-1822) | 30.2 (12.23-61.94) | 0 (0-0) | 0 (0-0) | 0.01 (0.01-0.01) |
| Solomon Islands | 53241 (47010-59395) | 19202.87 (17252.46-21303.04) | 17061 (15044-19261) | 6022.7 (5391.38-6723.69) | 96 (39-201) | 33.52 (13.69-68.97) | 113110 (100957-126722) | 19055.25 (17163.49-21179.14) | 35988 (31704-40605) | 5970.84 (5346.91-6661.67) | 203 (83-422) | 33.2 (13.51-68.65) | -0.02 (-0.02--0.02) | -0.02 (-0.03--0.02) | -0.03 (-0.03--0.02) |
| Somalia | 2676928 (2297639-3121666) | 36617.23 (32669.78-40926.91) | 1205535 (994054-1450817) | 15209.27 (13361.65-17436.94) | 6821 (2731-14615) | 84.61 (34.92-180.12) | 7122305 (6120055-8256681) | 36278.31 (32499.16-40338.15) | 3088455 (2556356-3701272) | 14670.28 (12954.37-16656.08) | 17501 (7063-36630) | 81.63 (34.27-171.07) | -0.04 (-0.05--0.03) | -0.1 (-0.11--0.08) | -0.09 (-0.11--0.07) |
| South Africa | 9775207 (8682459-10938104) | 30220.62 (26993.76-33462.99) | 3341434 (2931263-3767662) | 10228.46 (9076.71-11326.51) | 18681 (7577-38882) | 56.47 (22.87-116.87) | 16069822 (14308907-17939506) | 30022.16 (26817.01-33238.76) | 5433553 (4783927-6072502) | 10132.71 (9014.03-11223.52) | 30036 (12032-62423) | 55.67 (22.54-115.26) | -0.04 (-0.06--0.02) | -0.05 (-0.07--0.03) | -0.06 (-0.09--0.04) |
| South Sudan | 2005154 (1741424-2293592) | 36713.89 (32814.1-40841.6) | 889952 (742558-1064030) | 15296.59 (13562.41-17462.43) | 5011 (2033-10615) | 84.77 (35.39-177.86) | 3184057 (2765511-3650371) | 36052.73 (32216.89-40278.39) | 1339206 (1125222-1584903) | 14453.69 (12775.42-16510.91) | 7556 (3091-16003) | 80.13 (33.44-166.49) | -0.07 (-0.08--0.05) | -0.16 (-0.18--0.14) | -0.16 (-0.18--0.13) |
| Spain | 8360119 (7442301-9288703) | 18583.82 (16601.47-20460.82) | 2408325 (2188293-2674335) | 5359.18 (4890.17-5875.48) | 13212 (5435-27126) | 29.6 (12.22-60.81) | 12502656 (11164090-13997496) | 18335.24 (16365.8-20223.39) | 3634573 (3285234-4076606) | 5276.03 (4818.16-5795.54) | 19651 (8108-40321) | 29.15 (11.98-59.85) | -0.04 (-0.04--0.04) | -0.05 (-0.05--0.04) | -0.05 (-0.05--0.04) |
| Sri Lanka | 4698945 (4217185-5266957) | 29220.31 (26417.39-32433.99) | 2159426 (1955112-2348104) | 12995.1 (11887.09-14018.99) | 12168 (4943-25339) | 72.75 (29.63-152.1) | 6706833 (6061165-7479536) | 28844.84 (26057.48-32125.22) | 2972158 (2735634-3200909) | 12842.22 (11727.09-13868.72) | 16582 (6731-34600) | 71.82 (29.17-150.05) | -0.05 (-0.05--0.04) | -0.04 (-0.04--0.04) | -0.04 (-0.04--0.04) |
| Sudan | 1651621 (1491247-1825620) | 11434.2 (10305.19-12638.75) | 502263 (452479-556729) | 3421.44 (3116.86-3779.9) | 2802 (1128-5771) | 18.73 (7.61-38.1) | 3520066 (3153324-3906371) | 11195.83 (10080.7-12386.68) | 1063705 (954862-1185483) | 3337.81 (3033.63-3697.76) | 5936 (2401-12032) | 18.27 (7.46-36.73) | -0.07 (-0.07--0.06) | -0.08 (-0.08--0.07) | -0.08 (-0.08--0.07) |
| Suriname | 87677 (78635-97790) | 25904.85 (23208.28-28796.47) | 29027 (25924-32473) | 8577.87 (7779.64-9507.12) | 162 (66-340) | 47.45 (19.61-98.53) | 153017 (136850-171110) | 25730.95 (23047.82-28647) | 50474 (45720-56042) | 8514.07 (7721.23-9460.86) | 277 (115-577) | 46.85 (19.47-97.03) | -0.02 (-0.02--0.02) | -0.03 (-0.03--0.03) | -0.04 (-0.04--0.04) |
| Sweden | 2653442 (2370516-2966038) | 24309.26 (21679.72-26980.21) | 870782 (786931-974509) | 7962.64 (7175.46-8834.46) | 4757 (1979-9877) | 44.09 (18.03-90.53) | 3458456 (3062199-3867685) | 24104.11 (21482.27-26747.59) | 1142833 (1034072-1279870) | 7897.41 (7110.04-8760.86) | 6202 (2573-12918) | 43.71 (17.86-90.15) | -0.03 (-0.03--0.02) | -0.03 (-0.03--0.02) | -0.03 (-0.03--0.03) |
| Switzerland | 1937223 (1726521-2154093) | 23529.61 (21055.21-26042.79) | 643640 (584659-711497) | 7808.6 (7037.59-8654.43) | 3510 (1448-7233) | 43.05 (17.66-88.26) | 2867381 (2547481-3213946) | 23313.44 (20865.1-25815.2) | 961299 (873126-1071486) | 7742.5 (6973.79-8584.16) | 5201 (2140-10776) | 42.74 (17.57-87.8) | -0.03 (-0.03--0.02) | -0.03 (-0.03--0.02) | -0.02 (-0.02--0.01) |
| Syrian Arab Republic | 993380 (892674-1103076) | 11331.4 (10211.49-12530.42) | 300798 (270716-334877) | 3372.02 (3072.79-3727.05) | 1684 (674-3454) | 18.52 (7.56-37.78) | 1431923 (1284009-1591745) | 11026.91 (9910.63-12205.94) | 423380 (384331-469972) | 3265.46 (2975.1-3608.41) | 2329 (952-4760) | 17.83 (7.27-36.21) | -0.09 (-0.09--0.08) | -0.1 (-0.11--0.1) | -0.12 (-0.12--0.12) |
| Taiwan (Province of China) | 2507692 (2252571-2807738) | 13015.8 (11752.69-14462.61) | 727575 (653842-811763) | 3763.68 (3409.04-4155.41) | 4100 (1650-8571) | 21.07 (8.55-43.94) | 3700456 (3338704-4096377) | 12775.23 (11528.82-14227.65) | 1057684 (961568-1177445) | 3691.5 (3334.6-4072.48) | 5845 (2398-12097) | 20.65 (8.4-43.08) | -0.06 (-0.07--0.06) | -0.07 (-0.07--0.06) | -0.07 (-0.08--0.06) |
| Tajikistan | 743093 (662465-831508) | 18082.48 (16138.78-20033.78) | 228357 (204842-257533) | 5515.58 (5007.23-6138.73) | 1279 (517-2635) | 30.51 (12.38-62.44) | 1471019 (1317452-1646195) | 18084.9 (16155.62-20032.07) | 450238 (403466-506094) | 5518.78 (5017.26-6144.56) | 2523 (1010-5230) | 30.52 (12.35-62.4) | 0 (0-0) | 0 (0-0) | 0.01 (0.01-0.01) |
| Thailand | 14070281 (12481034-15945854) | 26616.59 (23911.86-29938.58) | 5673412 (4987267-6447186) | 10516.22 (9368.42-11857.1) | 31926 (12653-68080) | 58.72 (23.71-123.06) | 20408394 (18190530-22936323) | 26308.44 (23551.18-29628.84) | 8015282 (7093269-9053766) | 10394.18 (9243.16-11738.19) | 44431 (18106-92447) | 58.11 (23.45-121.76) | -0.04 (-0.04--0.04) | -0.04 (-0.04--0.04) | -0.03 (-0.04--0.03) |
| Timor-Leste | 176615 (156578-198632) | 27019.22 (24291.31-30377.67) | 70962 (62217-80390) | 10714.43 (9569.81-12069.19) | 400 (162-842) | 59.66 (24.21-124.61) | 335498 (297894-380709) | 26856.77 (24144.77-30150.01) | 134610 (118297-152394) | 10646.55 (9501.59-12002.23) | 757 (303-1599) | 59.37 (24.02-123.92) | -0.03 (-0.03--0.02) | -0.03 (-0.03--0.02) | -0.01 (-0.02--0.01) |
| Togo | 1203282 (1034671-1396423) | 36543.61 (32407.44-40797.57) | 534643 (437624-644720) | 15159.51 (13302.14-17214.4) | 3024 (1219-6474) | 84.45 (34.36-178.79) | 2736677 (2386575-3118401) | 36250.31 (32183.36-40524.98) | 1153036 (977419-1338984) | 14675.38 (12881.55-16558.57) | 6519 (2632-13738) | 81.93 (33.26-172.53) | -0.04 (-0.05--0.03) | -0.13 (-0.15--0.11) | -0.12 (-0.14--0.1) |
| Tokelau | 281 (252-311) | 19069.88 (17144.15-21200.25) | 89 (80-99) | 5963.54 (5342.08-6668.97) | 0 (0-1) | 33.23 (13.5-68.59) | 263 (236-293) | 18827.05 (16922.89-20914.29) | 82 (73-91) | 5864.26 (5231.05-6536.03) | 0 (0-1) | 32.58 (13.26-66.88) | -0.04 (-0.04--0.03) | -0.05 (-0.05--0.05) | -0.05 (-0.06--0.05) |
| Tonga | 16078 (14296-17930) | 18953.27 (17036.42-21093.07) | 5089 (4515-5718) | 5908.49 (5267.22-6597.6) | 29 (12-60) | 32.96 (13.36-68.23) | 18471 (16560-20534) | 18845.43 (16930.84-20948.35) | 5800 (5163-6459) | 5873.47 (5245.25-6548.64) | 32 (13-67) | 32.69 (13.36-67.3) | -0.02 (-0.02--0.02) | -0.02 (-0.02--0.02) | -0.02 (-0.03--0.02) |
| Trinidad and Tobago | 277527 (248790-308465) | 25853.3 (23154.36-28765.71) | 91735 (82202-102495) | 8547.31 (7738.21-9474.63) | 511 (211-1067) | 47.24 (19.57-97.73) | 405163 (362331-453266) | 25628.76 (22929.32-28524.43) | 133469 (120819-149147) | 8463.91 (7663.41-9386.12) | 730 (301-1519) | 46.63 (19.37-96.6) | -0.03 (-0.04--0.03) | -0.04 (-0.04--0.04) | -0.05 (-0.05--0.05) |
| Tunisia | 731981 (658631-812343) | 11253.71 (10131.94-12444.82) | 218895 (197484-243106) | 3340.74 (3039.91-3689.61) | 1221 (493-2500) | 18.35 (7.47-37.15) | 1367643 (1223489-1521022) | 11005.03 (9900.21-12172.2) | 403696 (366039-446553) | 3256.23 (2965.35-3600.21) | 2204 (897-4481) | 17.77 (7.26-36.26) | -0.07 (-0.08--0.07) | -0.09 (-0.09--0.09) | -0.1 (-0.1--0.1) |
| Turkmenistan | 514286 (458012-577217) | 18069.06 (16138-20013.52) | 157952 (141327-178107) | 5510.17 (5002.92-6136.24) | 885 (357-1838) | 30.45 (12.36-62.38) | 829443 (745526-918571) | 18066.25 (16128.4-20019.78) | 253122 (228368-283110) | 5508.84 (4998.68-6130.18) | 1410 (568-2926) | 30.46 (12.39-62.36) | 0 (0-0) | 0 (0-0) | 0.01 (0-0.01) |
| Tuvalu | 1642 (1471-1828) | 19230.25 (17278.49-21384.8) | 521 (464-582) | 6052.27 (5414.62-6740.44) | 3 (1-6) | 33.73 (13.81-69.7) | 2232 (1995-2491) | 19026.64 (17114.46-21159.44) | 702 (625-788) | 5955.95 (5332.84-6653.76) | 4 (2-8) | 33.16 (13.45-68.3) | -0.03 (-0.03--0.03) | -0.05 (-0.05--0.04) | -0.04 (-0.05--0.03) |
| T眉rkiye | 5235962 (4717139-5744726) | 11452.27 (10321.04-12650.71) | 1583196 (1441812-1751180) | 3428.88 (3142.73-3784.62) | 8828 (3612-18233) | 18.87 (7.74-38.44) | 9747298 (8747552-10833828) | 11074.79 (9977.38-12245.53) | 2884519 (2622215-3193384) | 3286.23 (2998.38-3630.1) | 15805 (6446-32149) | 18 (7.34-36.51) | -0.12 (-0.13--0.11) | -0.15 (-0.17--0.14) | -0.17 (-0.18--0.15) |
| Uganda | 5759104 (4980021-6616908) | 36063.71 (32222.31-40021.35) | 2498169 (2072984-2991432) | 14667.09 (13016.22-16781.32) | 14079 (5734-30016) | 81.33 (34.01-171.04) | 14215622 (12271104-16485466) | 36057.22 (32220.3-40278.65) | 6076623 (5047124-7290723) | 14474.38 (12798.53-16554.34) | 34428 (14055-74089) | 80.66 (33.26-168.03) | -0.02 (-0.03--0.01) | -0.06 (-0.08--0.03) | -0.03 (-0.06--0.01) |
| Ukraine | 10938659 (9691805-12257421) | 18419.35 (16468.41-20473.37) | 3339406 (3042327-3741098) | 5658.42 (5144.59-6293.3) | 18222 (7429-37596) | 31.05 (12.72-64.35) | 10608096 (9356831-11877152) | 18417.74 (16471.8-20457.32) | 3230991 (2932119-3642320) | 5658.66 (5144.75-6292.76) | 17483 (7122-35512) | 31.07 (12.74-64) | 0 (0-0) | 0 (0-0) | 0.01 (0.01-0.01) |
| United Arab Emirates | 154694 (136054-174356) | 11570.07 (10389.09-12727.17) | 47014 (41369-54053) | 3463.97 (3147.48-3825.44) | 265 (106-538) | 18.99 (7.71-38.28) | 950381 (828138-1093303) | 11344.46 (10150.55-12491.26) | 282833 (246589-327310) | 3377.42 (3077.8-3745.18) | 1583 (636-3257) | 18.48 (7.58-37.57) | -0.06 (-0.06--0.05) | -0.08 (-0.09--0.07) | -0.08 (-0.09--0.07) |
| United Kingdom | 16728155 (14898936-18578521) | 24227.41 (21627.76-26836.61) | 5492616 (4977249-6131930) | 7953.89 (7182.88-8830.44) | 30073 (12488-62435) | 43.98 (18.1-90.36) | 21614427 (19223323-24131053) | 23997.56 (21388.75-26619.1) | 7139899 (6467656-7988990) | 7881.23 (7102.41-8757.08) | 38759 (16077-80526) | 43.5 (17.87-89.47) | -0.03 (-0.04--0.03) | -0.03 (-0.04--0.03) | -0.04 (-0.04--0.03) |
| United Republic of Tanzania | 8531434 (7385986-9818093) | 35777.31 (31923.62-39840.11) | 3629669 (3279700-4077072) | 14452.34 (13308.45-15837.77) | 20407 (8382-42551) | 80.08 (32.87-166.24) | 19913225 (17299553-22919603) | 36738.04 (32799.29-40847.39) | 8667917 (7890776-9404155) | 15145.09 (13965.49-16398.37) | 49083 (19641-101466) | 84.61 (34.26-175.93) | 0 (-0.03-0.03) | -0.01 (-0.08-0.05) | 0.02 (-0.05-0.08) |
| United States of America | 23546132 (21759507-25188972) | 8282.02 (7684.81-8834.58) | 7133690 (6835528-7422895) | 2505.81 (2399.74-2611.53) | 38907 (15934-79887) | 13.76 (5.61-28.35) | 36663960 (33839814-39273511) | 8400.75 (7814.24-8916.19) | 10947152 (10532222-11390673) | 2509.04 (2416.84-2590.13) | 58556 (24201-118692) | 13.67 (5.58-28) | -0.01 (-0.03-0.01) | -0.04 (-0.06--0.03) | -0.07 (-0.08--0.05) |
| United States Virgin Islands | 25013 (22522-27850) | 25691.14 (23004.58-28593.04) | 8193 (7386-9146) | 8468.12 (7679.53-9412.26) | 46 (19-95) | 46.92 (19.45-96.61) | 30439 (27000-34663) | 25534.49 (22834.73-28417.18) | 10085 (9025-11392) | 8417.05 (7635-9331.99) | 55 (22-113) | 46.41 (19.21-95.88) | -0.02 (-0.02--0.01) | -0.02 (-0.02--0.01) | -0.03 (-0.04--0.03) |
| Uruguay | 657625 (586432-729284) | 19243.04 (17235.77-21285.15) | 201404 (182378-222616) | 5903.82 (5351.33-6502.76) | 1108 (457-2275) | 32.61 (13.42-66.8) | 825498 (737023-919795) | 19028.1 (17000.94-21076.97) | 253690 (229734-280960) | 5825.14 (5278.45-6424.86) | 1378 (569-2808) | 32.08 (13.19-65.14) | -0.03 (-0.04--0.03) | -0.04 (-0.05--0.04) | -0.05 (-0.05--0.04) |
| Uzbekistan | 2995954 (2670988-3351434) | 18061.65 (16124.7-20003.38) | 919825 (827867-1035727) | 5507.06 (4999.86-6130.56) | 5137 (2087-10603) | 30.41 (12.4-62.26) | 5454395 (4912025-6054349) | 18058.91 (16122.81-20005.75) | 1661582 (1495467-1857508) | 5506.98 (4997.59-6135.25) | 9253 (3740-19071) | 30.4 (12.38-61.99) | 0 (0-0) | 0 (0-0) | 0 (0-0) |
| Vanuatu | 24036 (21325-26805) | 19157.37 (17199.78-21269.17) | 7667 (6777-8648) | 6000.87 (5372.51-6707.67) | 43 (18-90) | 33.43 (13.64-68.54) | 52447 (46753-58625) | 19092.63 (17185.75-21203.87) | 16695 (14755-18776) | 5990.5 (5362.58-6682.89) | 94 (38-196) | 33.34 (13.46-68.63) | -0.01 (-0.01--0.01) | 0 (0-0) | 0 (0-0.01) |
| Venezuela (Bolivarian Republic of) | 2846890 (2559943-3158193) | 18501.59 (16633.89-20419.1) | 860327 (772443-962895) | 5513.9 (4992.39-6116.7) | 4823 (1972-9976) | 30.46 (12.52-63) | 5024651 (4515279-5571701) | 18200.99 (16352.36-20111.29) | 1485673 (1344994-1645704) | 5402.81 (4892.28-5992.8) | 8186 (3324-16714) | 29.81 (12.13-61.21) | -0.05 (-0.05--0.04) | -0.06 (-0.07--0.05) | -0.06 (-0.07--0.05) |
| Viet Nam | 16013354 (14162759-18128225) | 26652.75 (23958.68-30001.26) | 6361647 (5593988-7194766) | 10523.93 (9383.56-11858.28) | 35856 (14238-75811) | 58.87 (23.81-123) | 26822342 (24038332-30154666) | 26400.14 (23695.28-29738.29) | 10692313 (9426060-12167692) | 10438.34 (9293.78-11786.19) | 60035 (24139-126463) | 58.52 (23.59-123.02) | -0.03 (-0.03--0.03) | -0.03 (-0.03--0.03) | -0.02 (-0.02--0.02) |
| Yemen | 1029012 (924063-1144367) | 11372.03 (10246.69-12556.14) | 314659 (282571-351725) | 3396.23 (3085.78-3755.85) | 1759 (707-3597) | 18.55 (7.56-37.5) | 2656465 (2379046-2950639) | 11194.9 (10084.19-12401.39) | 806683 (725446-901511) | 3342.66 (3041.8-3695.18) | 4494 (1830-9150) | 18.25 (7.49-36.85) | -0.06 (-0.06--0.05) | -0.06 (-0.06--0.06) | -0.06 (-0.06--0.06) |
| Zambia | 2718571 (2348790-3130594) | 36879.17 (32881.93-41247.47) | 1257181 (1027254-1516586) | 15682.7 (13633.6-18000.8) | 7102 (2865-14974) | 87.27 (36.39-184.17) | 6440586 (5578465-7379911) | 36449.28 (32563.96-40676.99) | 2806575 (2319982-3318542) | 14923.06 (13093.22-16999.49) | 15863 (6474-33468) | 83.02 (34.36-173.88) | -0.05 (-0.07--0.04) | -0.17 (-0.2--0.13) | -0.16 (-0.19--0.12) |
| Zimbabwe | 2493285 (2215849-2814333) | 29229.75 (26148.46-32414.42) | 877771 (761258-1017424) | 10096.75 (9091.52-11252.56) | 4953 (2026-10386) | 56.01 (22.79-115.62) | 3851234 (3412204-4320539) | 29365.65 (26260.25-32497.13) | 1353425 (1178210-1547613) | 10158.41 (9161.18-11259.45) | 7617 (3094-15989) | 56.19 (22.64-115.65) | 0.02 (0.01-0.04) | 0.04 (0.02-0.06) | 0.04 (0.02-0.05) |

**Supplementary Table S2** Predictions of trends in age-Standardized rates and cases from 2022 to 2046 by APC model

| year | sex | Age-standardized incidence rate/100000 | Number of incidence cases | Age-standardized prevalence rate/10000 | Number of prevalence cases | Age-standardized DALYs rate/100000 | Number of DALYs cases |
| --- | --- | --- | --- | --- | --- | --- | --- |
| 2022 | Female | 21106.78 | 878768609.3 | 7421.3 | 304803207.3 | 41.17 | 1683897.254 |
| 2023 | Female | 21103.93 | 891222809.2 | 7422.46 | 308919936.3 | 41.17 | 1705636.893 |
| 2024 | Female | 21101.09 | 903973454.4 | 7423.61 | 313133060.1 | 41.17 | 1727795.733 |
| 2025 | Female | 21117.21 | 917546416.8 | 7432.21 | 317649808.3 | 41.22 | 1751684.174 |
| 2026 | Female | 21133.33 | 931085905.5 | 7440.82 | 322142809.2 | 41.26 | 1775412.59 |
| 2027 | Female | 21149.45 | 944483037.4 | 7449.42 | 326575776.2 | 41.31 | 1798806.342 |
| 2028 | Female | 21165.57 | 957978344.8 | 7458.03 | 331028798.8 | 41.35 | 1822250.327 |
| 2029 | Female | 21181.69 | 971745611.4 | 7466.63 | 335560326.4 | 41.39 | 1846023.991 |
| 2030 | Female | 21197.83 | 985721425.2 | 7474.69 | 340133697.8 | 41.43 | 1870013.915 |
| 2031 | Female | 21213.97 | 999685103 | 7482.75 | 344699397 | 41.48 | 1893923.684 |
| 2032 | Female | 21230.11 | 1013503862 | 7490.81 | 349216695.4 | 41.52 | 1917552.725 |
| 2033 | Female | 21246.25 | 1027433958 | 7498.88 | 353777959.2 | 41.56 | 1941367.822 |
| 2034 | Female | 21262.39 | 1041613680 | 7506.94 | 358420495.7 | 41.6 | 1965533.457 |
| 2035 | Female | 21277.93 | 1055917065 | 7514.22 | 363082697.1 | 41.64 | 1989808.563 |
| 2036 | Female | 21293.46 | 1070077716 | 7521.51 | 367701328.6 | 41.68 | 2013822.856 |
| 2037 | Female | 21308.99 | 1083916524 | 7528.8 | 372218888.7 | 41.72 | 2037290.942 |
| 2038 | Female | 21324.53 | 1097622689 | 7536.09 | 376705887.8 | 41.76 | 2060559.286 |
| 2039 | Female | 21340.06 | 1111385753 | 7543.37 | 381219825.2 | 41.8 | 2083903.847 |
| 2040 | Female | 21355.63 | 1125183467 | 7550.55 | 385752906.8 | 41.83 | 2107293.824 |
| 2041 | Female | 21371.19 | 1138751656 | 7557.74 | 390211275.4 | 41.87 | 2130272.072 |
| 2042 | Female | 21386.76 | 1151963986 | 7564.92 | 394550037 | 41.91 | 2152622.243 |
| 2043 | Female | 21402.32 | 1164982145 | 7572.1 | 398822676.4 | 41.95 | 2174596.567 |
| 2044 | Female | 21417.89 | 1177972974 | 7579.28 | 403085129.5 | 41.98 | 2196464.061 |
| 2045 | Female | 21433.46 | 1190816613 | 7586.46 | 407298781.7 | 42.02 | 2218042.861 |
| 2046 | Female | 21449.02 | 1203402768 | 7593.64 | 411429063.6 | 42.06 | 2239169.408 |
| 2022 | Male | 22413.63 | 895676794.6 | 8260.55 | 327758474.2 | 46.19 | 1833720.072 |
| 2023 | Male | 22412.65 | 907341807.9 | 8260.83 | 331709569.6 | 46.18 | 1854812.165 |
| 2024 | Male | 22411.68 | 919205555.6 | 8261.11 | 335715350.2 | 46.18 | 1876106.76 |
| 2025 | Male | 22427.01 | 931725383 | 8269.58 | 340027946.6 | 46.22 | 1899177.456 |
| 2026 | Male | 22442.34 | 944101597.1 | 8278.05 | 344275548.1 | 46.26 | 1921863.451 |
| 2027 | Male | 22457.67 | 956230971.2 | 8286.52 | 348425759.2 | 46.3 | 1944009.442 |
| 2028 | Male | 22473 | 968348086.6 | 8294.99 | 352557402.8 | 46.35 | 1966001.128 |
| 2029 | Male | 22488.34 | 980620163 | 8303.47 | 356725021.7 | 46.39 | 1988103.041 |
| 2030 | Male | 22503.8 | 992952176.5 | 8311.48 | 360884091.3 | 46.43 | 2010153.25 |
| 2031 | Male | 22519.27 | 1005170522 | 8319.5 | 364994829.2 | 46.47 | 2031909.579 |
| 2032 | Male | 22534.74 | 1017158138 | 8327.51 | 369018870.3 | 46.51 | 2053181.236 |
| 2033 | Male | 22550.21 | 1029199445 | 8335.53 | 373066067.7 | 46.55 | 2074541.373 |
| 2034 | Male | 22565.68 | 1041407973 | 8343.54 | 377160902.5 | 46.59 | 2096085.985 |
| 2035 | Male | 22580.83 | 1053656069 | 8350.92 | 381245589.6 | 46.63 | 2117585.572 |
| 2036 | Male | 22595.97 | 1065747059 | 8358.3 | 385274424.6 | 46.67 | 2138761.188 |
| 2037 | Male | 22611.12 | 1077539500 | 8365.69 | 389201268.1 | 46.71 | 2159382.117 |
| 2038 | Male | 22626.26 | 1089233142 | 8373.07 | 393103545.3 | 46.75 | 2179840.697 |
| 2039 | Male | 22641.41 | 1100996667 | 8380.45 | 397029375.8 | 46.78 | 2200366.371 |
| 2040 | Male | 22656.28 | 1112746966 | 8387.72 | 400958951.9 | 46.82 | 2220866.823 |
| 2041 | Male | 22671.15 | 1124310925 | 8394.99 | 404824760.7 | 46.86 | 2241008.562 |
| 2042 | Male | 22686.02 | 1135578096 | 8402.27 | 408588981.6 | 46.9 | 2260606.241 |
| 2043 | Male | 22700.89 | 1146691375 | 8409.54 | 412300515.3 | 46.93 | 2279894.351 |
| 2044 | Male | 22715.76 | 1157802503 | 8416.81 | 416008575.2 | 46.97 | 2299112.358 |
| 2045 | Male | 22730.63 | 1168802465 | 8424.08 | 419677618.4 | 47.01 | 2318089.166 |
| 2046 | Male | 22745.49 | 1179591354 | 8431.35 | 423275487.7 | 47.05 | 2336669.286 |

**Supplementary Table S3** Predictions of trends in age-Standardized rates and cases from 2022 to 2046 by ARIMA model

| year | sex | Age-standardized incidence rate/100000 | Number of incidence cases | Age-standardized prevalence rate/100000 | Number of prevalence cases | Age-standardized DALYs rate/100000 | Number of DALYs cases |
| --- | --- | --- | --- | --- | --- | --- | --- |
| 2022 | Male | 22256.92925 | 880071091.9 | 8227.737612 | 323236451.9 | 46.00922099 | 1808162.654 |
| 2023 | Male | 22275.22924 | 888926789.2 | 8266.65721 | 327825159.6 | 46.21948537 | 1833122.474 |
| 2024 | Male | 22293.52922 | 897782486.5 | 8305.576808 | 332052703.4 | 46.42974975 | 1855540.411 |
| 2025 | Male | 22311.82921 | 906638183.8 | 8344.496406 | 336544087.4 | 46.64001413 | 1879913.709 |
| 2026 | Male | 22330.12919 | 915493881 | 8383.416004 | 340842728.8 | 46.85027851 | 1902782.832 |
| 2027 | Male | 22348.42918 | 924349578.3 | 8422.335602 | 345282174 | 47.06054289 | 1926809.052 |
| 2028 | Male | 22366.72916 | 933205275.6 | 8461.2552 | 349618758.1 | 47.27080727 | 1949945.167 |
| 2029 | Male | 22385.02915 | 942060972.9 | 8500.174798 | 354030485.1 | 47.48107165 | 1973766.002 |
| 2030 | Male | 22403.32913 | 950916670.2 | 8539.094396 | 358387318.1 | 47.69133603 | 1997060.111 |
| 2031 | Male | 22421.62912 | 959772367.5 | 8578.013994 | 362784252.7 | 47.90160041 | 2020759.407 |
| 2032 | Male | 22439.9291 | 968628064.7 | 8616.933592 | 367151892 | 48.11186479 | 2044147.011 |
| 2033 | Male | 22458.22909 | 977483762 | 8655.85319 | 371540932.4 | 48.32212917 | 2067774.386 |
| 2034 | Male | 22476.52907 | 986339459.3 | 8694.772788 | 375914338.7 | 48.53239355 | 2091217.315 |
| 2035 | Male | 22494.82906 | 995195156.6 | 8733.692386 | 380299166 | 48.74265793 | 2114802.131 |
| 2036 | Male | 22513.12904 | 1004050854 | 8772.611984 | 384675650 | 48.95292231 | 2138277.8 |
| 2037 | Male | 22531.42903 | 1012906551 | 8811.531582 | 389058229.1 | 49.16318669 | 2161837.43 |
| 2038 | Male | 22549.72901 | 1021762248 | 8850.45118 | 393436355.5 | 49.37345107 | 2185332.472 |
| 2039 | Male | 22568.029 | 1030617946 | 8889.370778 | 397817734.7 | 49.58371545 | 2208877.2 |
| 2040 | Male | 22586.32898 | 1039473643 | 8928.290376 | 402196737.6 | 49.79397983 | 2232383.706 |
| 2041 | Male | 22604.62897 | 1048329340 | 8967.209974 | 406577476.5 | 50.00424421 | 2255919.615 |
| 2042 | Male | 22622.92895 | 1057185038 | 9006.129572 | 410956947.2 | 50.21450859 | 2279432.905 |
| 2043 | Male | 22641.22894 | 1066040735 | 9045.04917 | 415337344.4 | 50.42477297 | 2302963.595 |
| 2044 | Male | 22659.52892 | 1074896432 | 9083.968768 | 419717064.7 | 50.63503735 | 2326480.9 |
| 2045 | Male | 22677.82891 | 1083752129 | 9122.888366 | 424097279.5 | 50.84530173 | 2350008.501 |
| 2046 | Male | 22696.12889 | 1092607827 | 9161.807964 | 428477133.1 | 51.05556611 | 2373528.183 |
| 2047 | Male | 22714.42888 | 1101463524 | 9200.727562 | 432857250.5 | 51.26583049 | 2397053.956 |
| 2048 | Male | 22732.72886 | 1110319221 | 9239.64716 | 437237175.2 | 51.47609487 | 2420575.043 |
| 2049 | Male | 22751.02885 | 1119174919 | 9278.566758 | 441617240.7 | 51.68635925 | 2444099.735 |
| 2050 | Male | 22769.32883 | 1128030616 | 9317.486356 | 445997203.4 | 51.89662363 | 2467621.654 |
| 2022 | Female | 21091.91113 | 873033616.5 | 7389.421769 | 302025334.1 | 40.9807264 | 1667289.454 |
| 2023 | Female | 21117.80038 | 887496362.6 | 7402.449236 | 306660513.7 | 41.04511453 | 1691589.239 |
| 2024 | Female | 21143.68962 | 901959108.7 | 7415.476702 | 311295693.3 | 41.10950266 | 1715889.025 |
| 2025 | Female | 21169.57887 | 916421854.8 | 7428.504169 | 315930872.9 | 41.17389079 | 1740188.81 |
| 2026 | Female | 21195.46812 | 930884600.9 | 7441.531635 | 320566052.5 | 41.23827891 | 1764488.596 |
| 2027 | Female | 21221.35737 | 945347347 | 7454.559102 | 325201232.1 | 41.30266704 | 1788788.381 |
| 2028 | Female | 21247.24661 | 959810093.1 | 7467.586568 | 329836411.7 | 41.36705517 | 1813088.167 |
| 2029 | Female | 21273.13586 | 974272839.2 | 7480.614035 | 334471591.3 | 41.4314433 | 1837387.952 |
| 2030 | Female | 21299.02511 | 988735585.4 | 7493.641501 | 339106770.9 | 41.49583143 | 1861687.738 |
| 2031 | Female | 21324.91436 | 1003198331 | 7506.668968 | 343741950.5 | 41.56021956 | 1885987.523 |
| 2032 | Female | 21350.80361 | 1017661078 | 7519.696434 | 348377130.1 | 41.62460769 | 1910287.309 |
| 2033 | Female | 21376.69285 | 1032123824 | 7532.723901 | 353012309.7 | 41.68899582 | 1934587.094 |
| 2034 | Female | 21402.5821 | 1046586570 | 7545.751367 | 357647489.3 | 41.75338395 | 1958886.88 |
| 2035 | Female | 21428.47135 | 1061049316 | 7558.778834 | 362282668.9 | 41.81777208 | 1983186.665 |
| 2036 | Female | 21454.3606 | 1075512062 | 7571.8063 | 366917848.5 | 41.8821602 | 2007486.451 |
| 2037 | Female | 21480.24984 | 1089974808 | 7584.833767 | 371553028.1 | 41.94654833 | 2031786.236 |
| 2038 | Female | 21506.13909 | 1104437554 | 7597.861233 | 376188207.7 | 42.01093646 | 2056086.022 |
| 2039 | Female | 21532.02834 | 1118900300 | 7610.8887 | 380823387.3 | 42.07532459 | 2080385.807 |
| 2040 | Female | 21557.91759 | 1133363046 | 7623.916166 | 385458566.9 | 42.13971272 | 2104685.593 |
| 2041 | Female | 21583.80683 | 1147825793 | 7636.943633 | 390093746.4 | 42.20410085 | 2128985.378 |
| 2042 | Female | 21609.69608 | 1162288539 | 7649.971099 | 394728926 | 42.26848898 | 2153285.164 |
| 2043 | Female | 21635.58533 | 1176751285 | 7662.998566 | 399364105.6 | 42.33287711 | 2177584.95 |
| 2044 | Female | 21661.47458 | 1191214031 | 7676.026032 | 403999285.2 | 42.39726524 | 2201884.735 |
| 2045 | Female | 21687.36383 | 1205676777 | 7689.053499 | 408634464.8 | 42.46165337 | 2226184.521 |
| 2046 | Female | 21713.25307 | 1220139523 | 7702.080965 | 413269644.4 | 42.52604149 | 2250484.306 |
| 2047 | Female | 21739.14232 | 1234602269 | 7715.108432 | 417904824 | 42.59042962 | 2274784.092 |
| 2048 | Female | 21765.03157 | 1249065015 | 7728.135898 | 422540003.6 | 42.65481775 | 2299083.877 |
| 2049 | Female | 21790.92082 | 1263527761 | 7741.163365 | 427175183.2 | 42.71920588 | 2323383.663 |
| 2050 | Female | 21816.81006 | 1277990507 | 7754.190831 | 431810362.8 | 42.78359401 | 2347683.448 |
