# Supplementary figures and images for: Worldwide trends and future projections of fungal skin disease burden: a comprehensive analysis from the Global Burden of Diseases study 2021
Source: Front Public Health. 2025 Jun 4;13:1580221. doi: 10.3389/fpubh.2025.1580221 (PMC12174046; doi:10.3389/fpubh.2025.1580221)

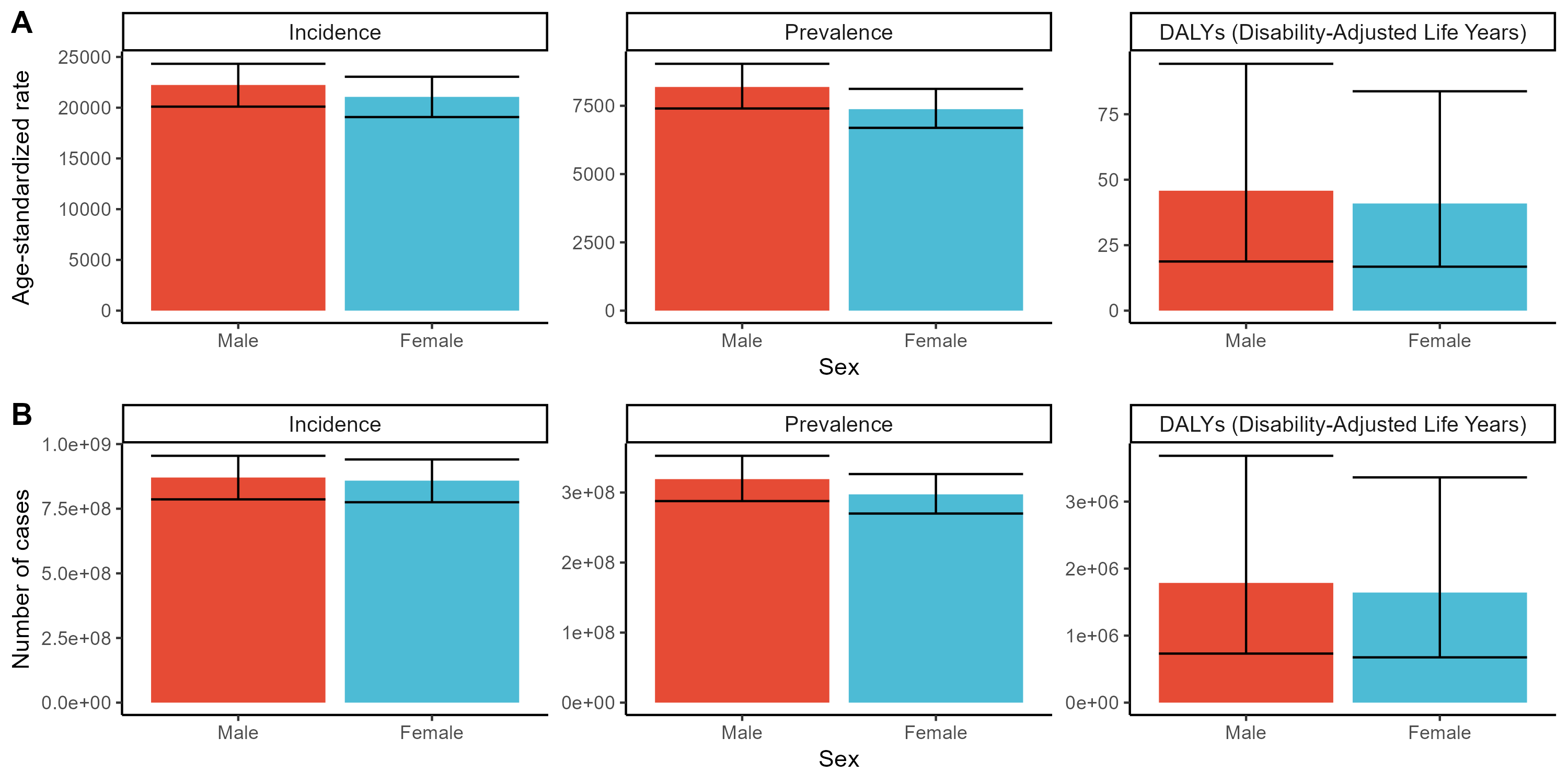

Supplement: Supplementary file 1 [file Data_Sheet_1.ZIP › Supplementary information/SFig. 1 21sex.png]

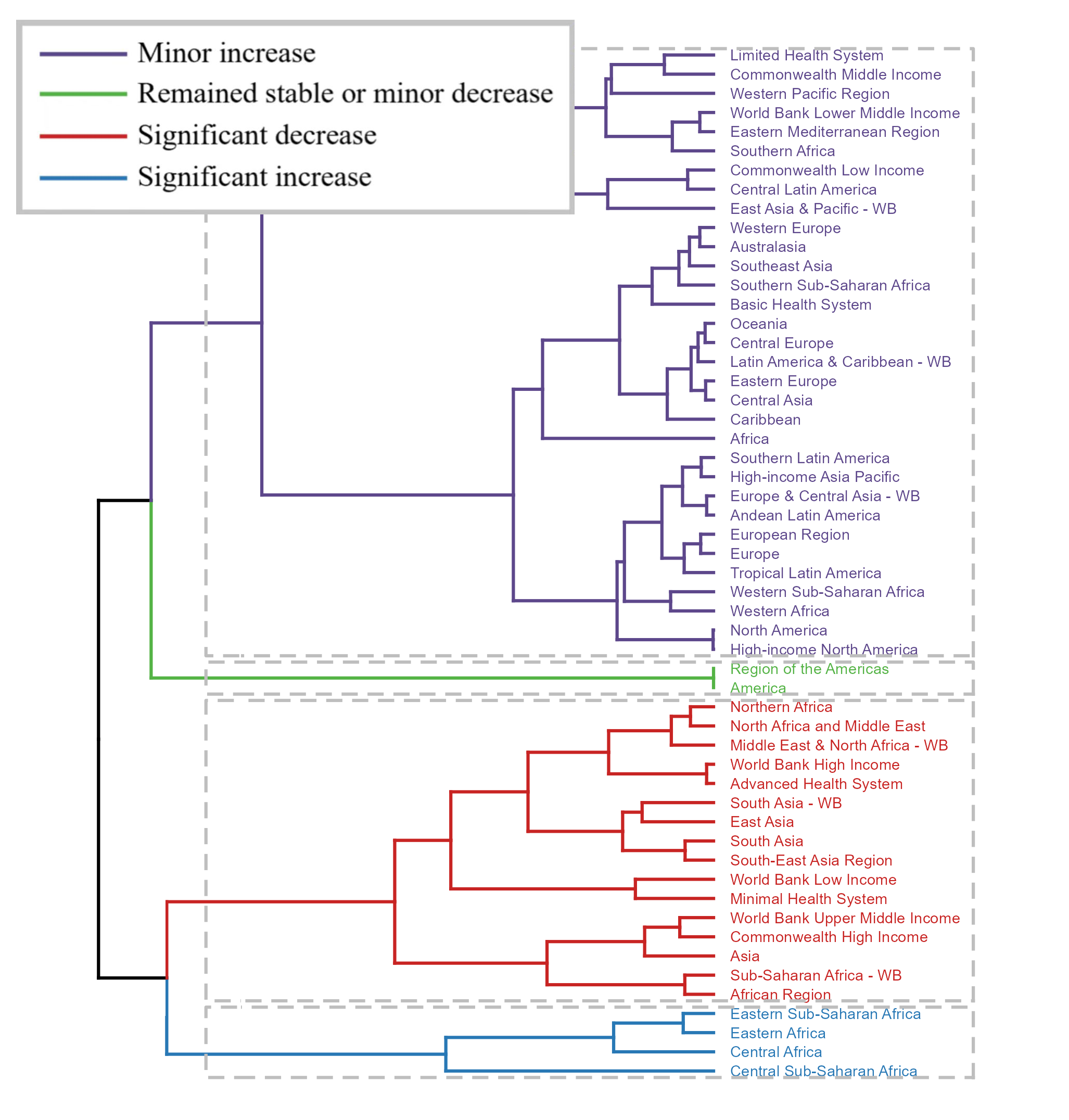

Supplement: Supplementary file 1 [file Data_Sheet_1.ZIP › Supplementary information/SFig. 2 90-21GBD region.png]

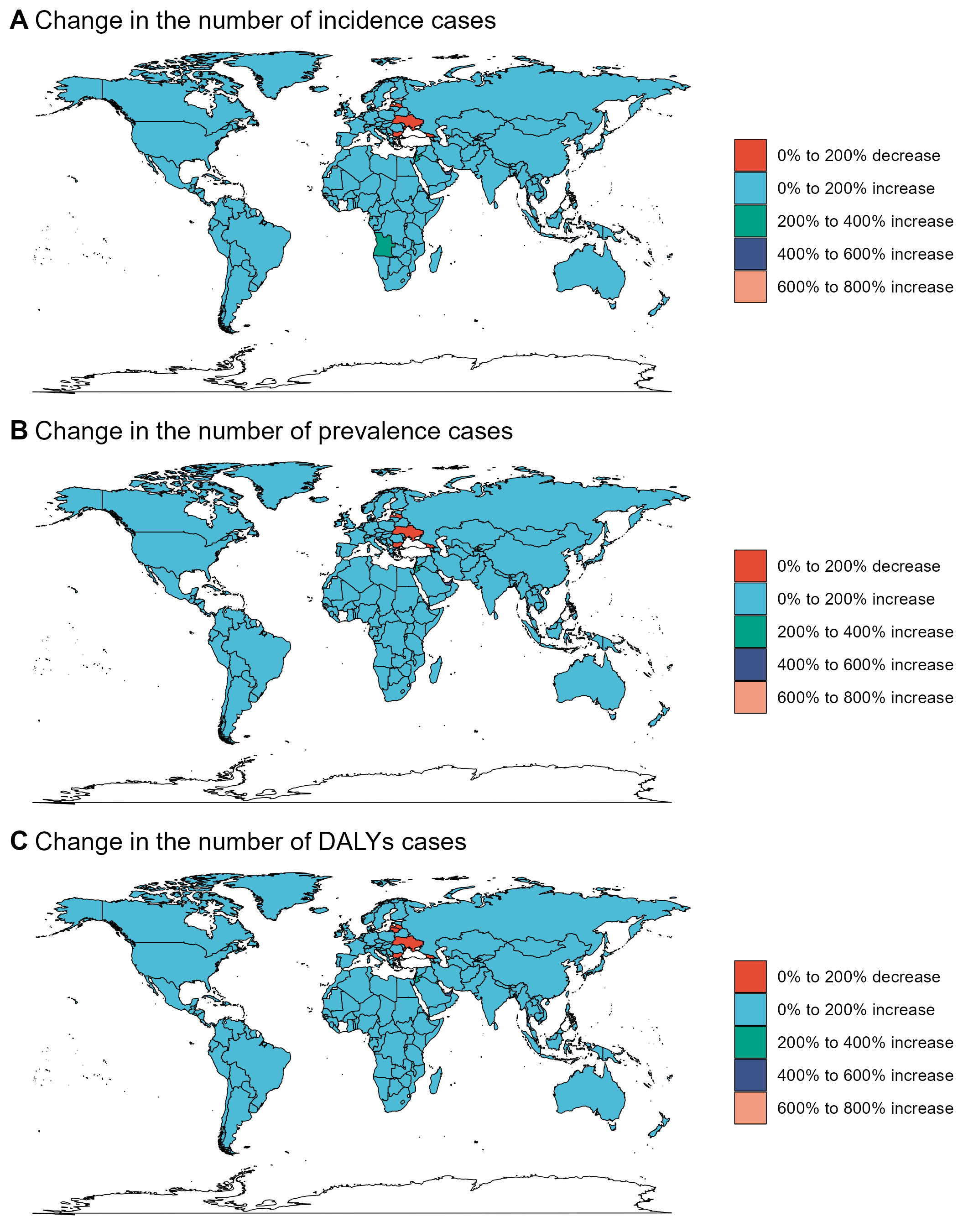

Supplement: Supplementary file 1 [file Data_Sheet_1.ZIP › Supplementary information/SFig. 3 90-21country number.png]

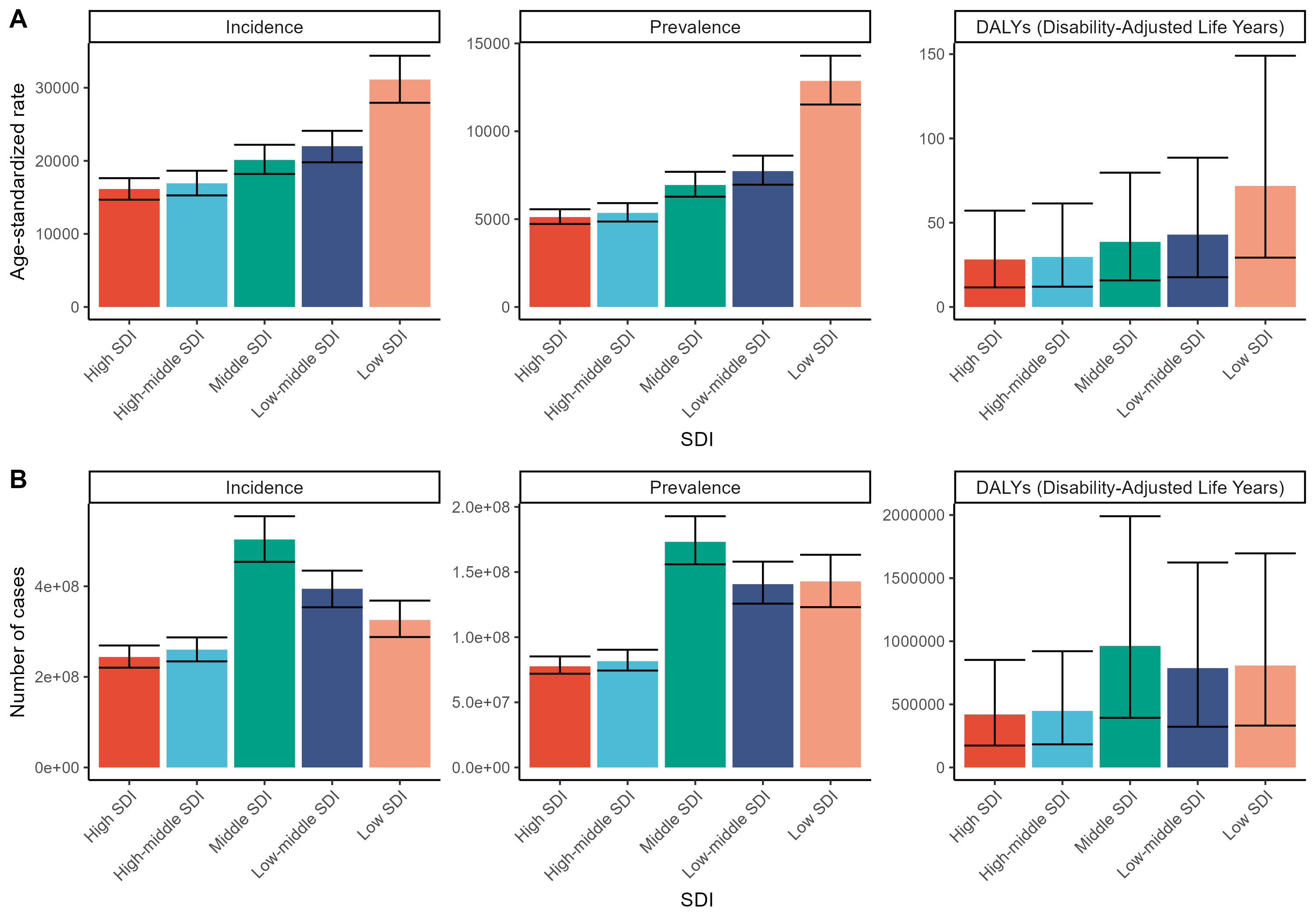

Supplement: Supplementary file 1 [file Data_Sheet_1.ZIP › Supplementary information/SFig. 4 21SDI.png]

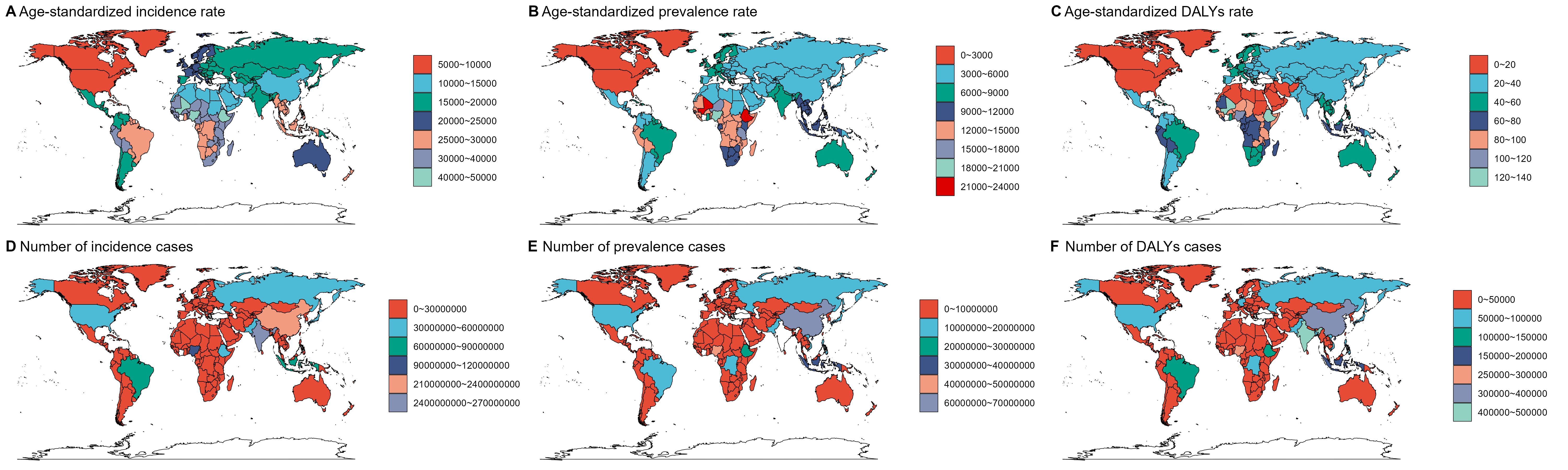

Supplement: Supplementary file 1 [file Data_Sheet_1.ZIP › Supplementary information/SFig. 5 21country.png]

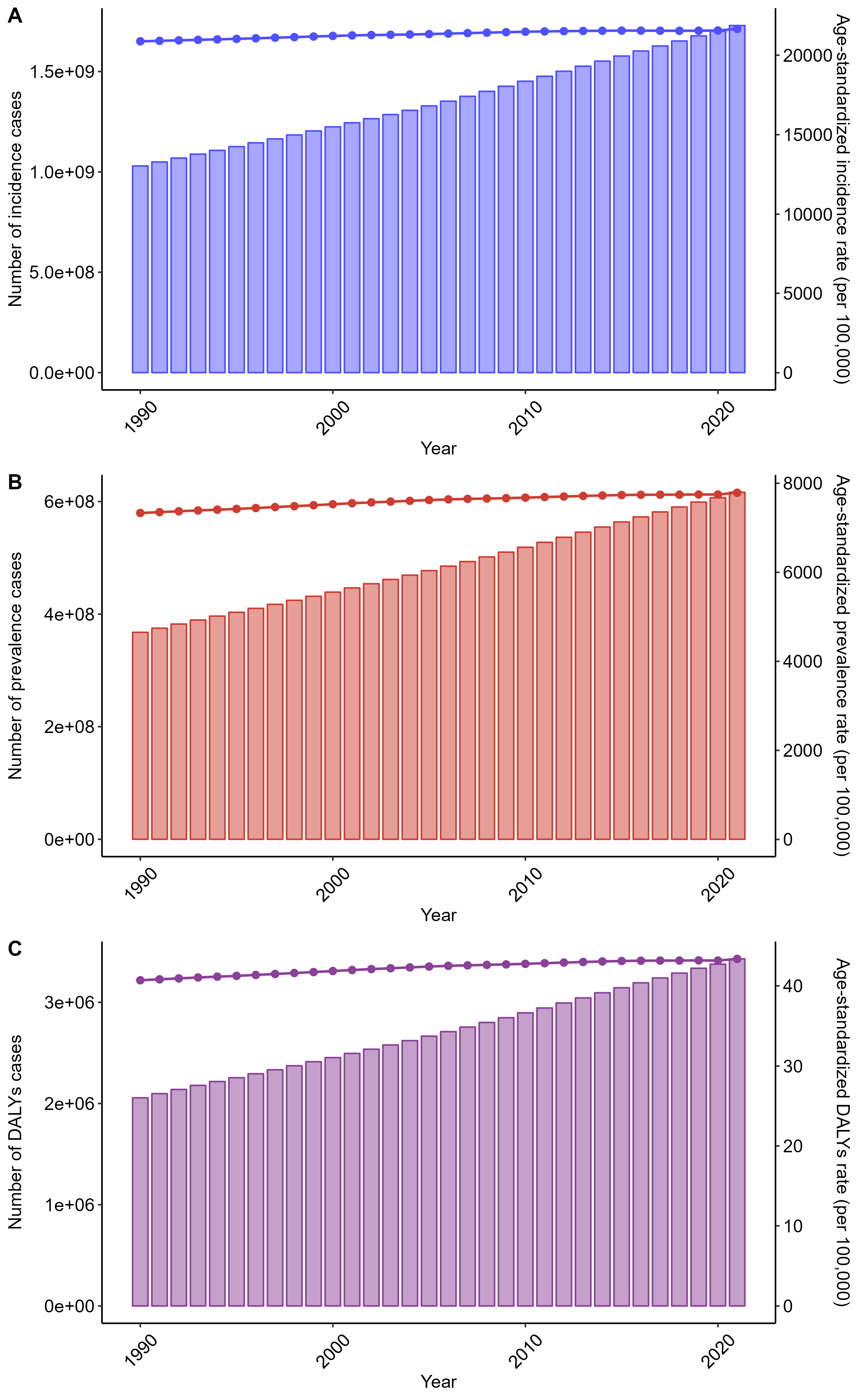

Supplement: Supplementary file 1 [file Data_Sheet_1.ZIP › Supplementary information/SFig. 6 90-21.png]

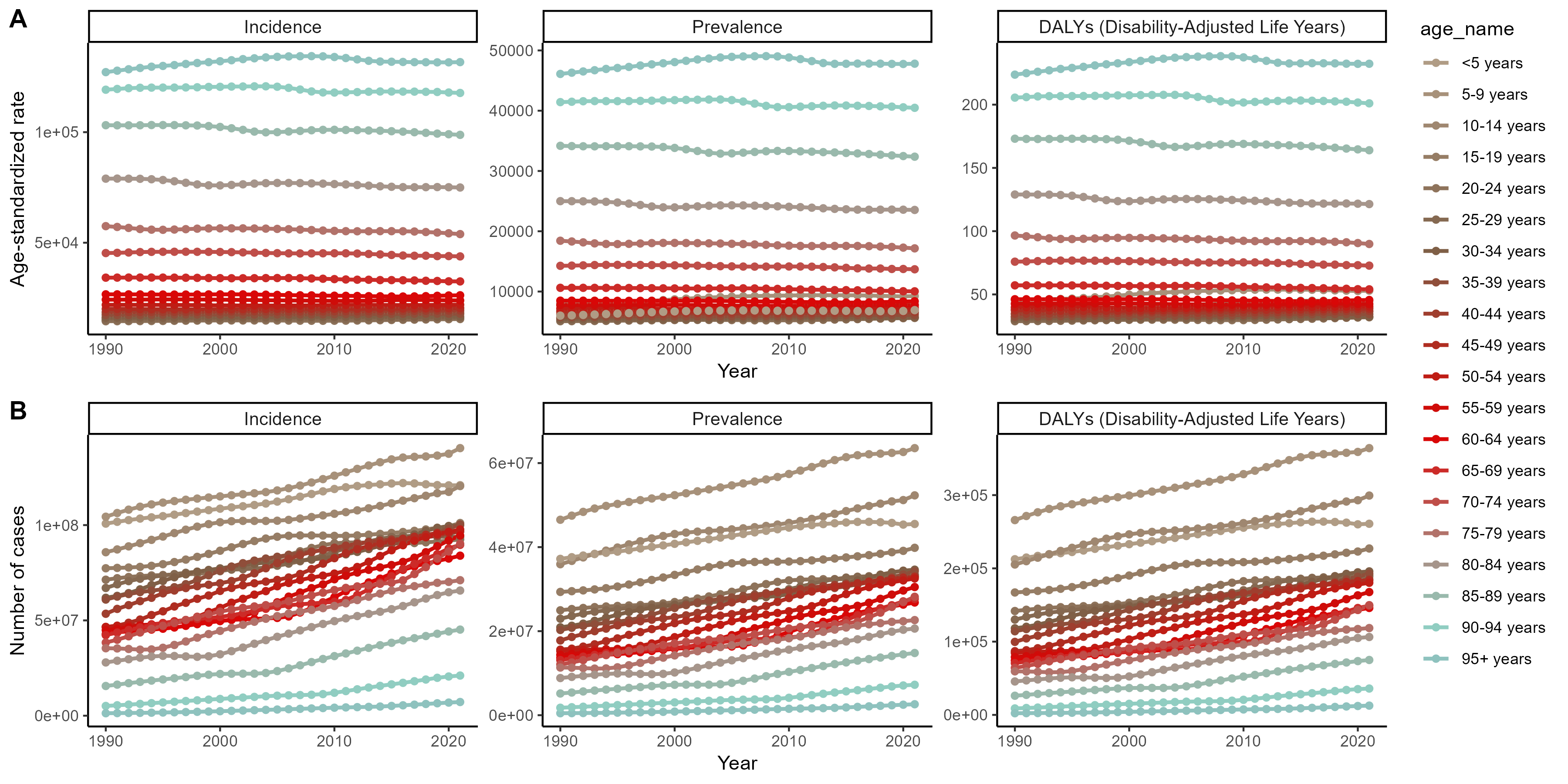

Supplement: Supplementary file 1 [file Data_Sheet_1.ZIP › Supplementary information/SFig. 7 90-21age.png]

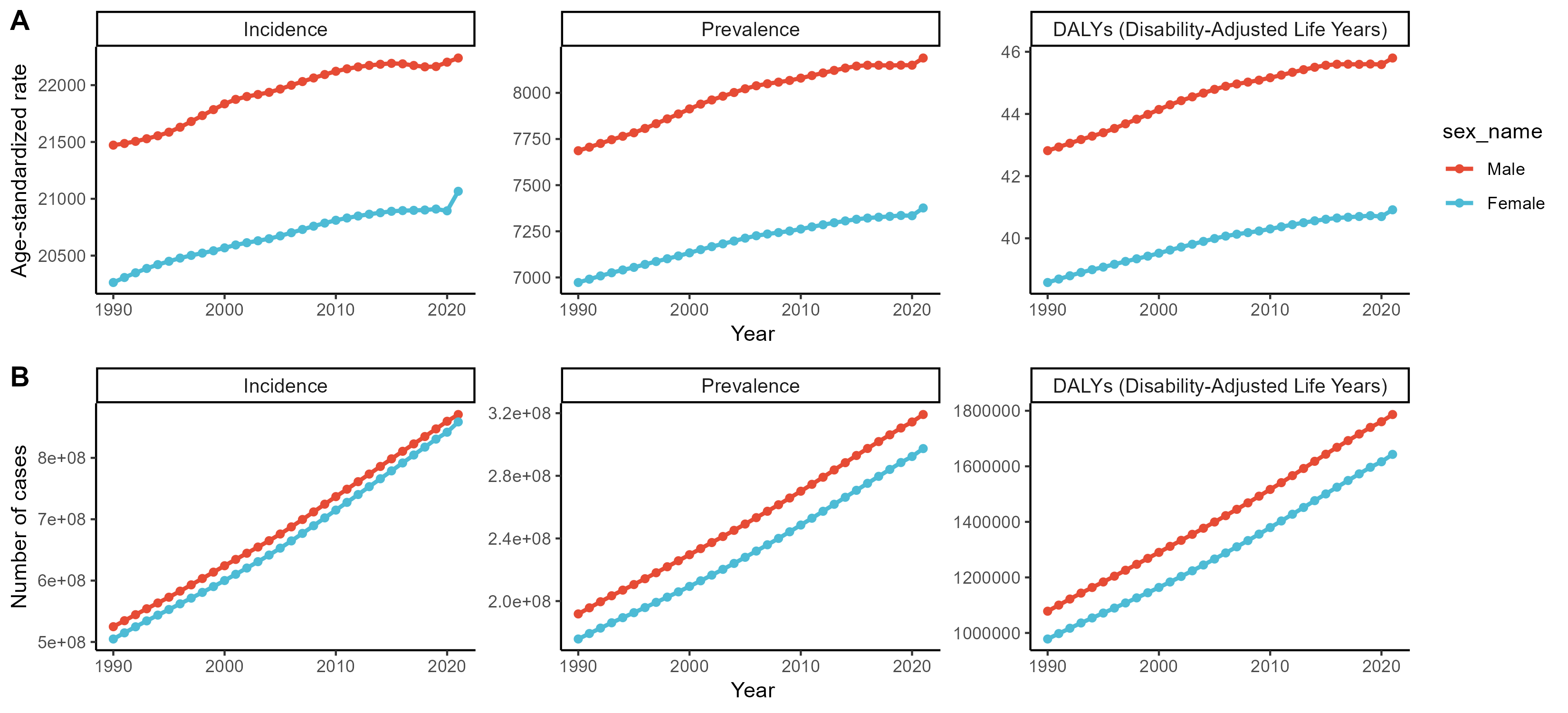

Supplement: Supplementary file 1 [file Data_Sheet_1.ZIP › Supplementary information/SFig. 8 90-21sex.png]

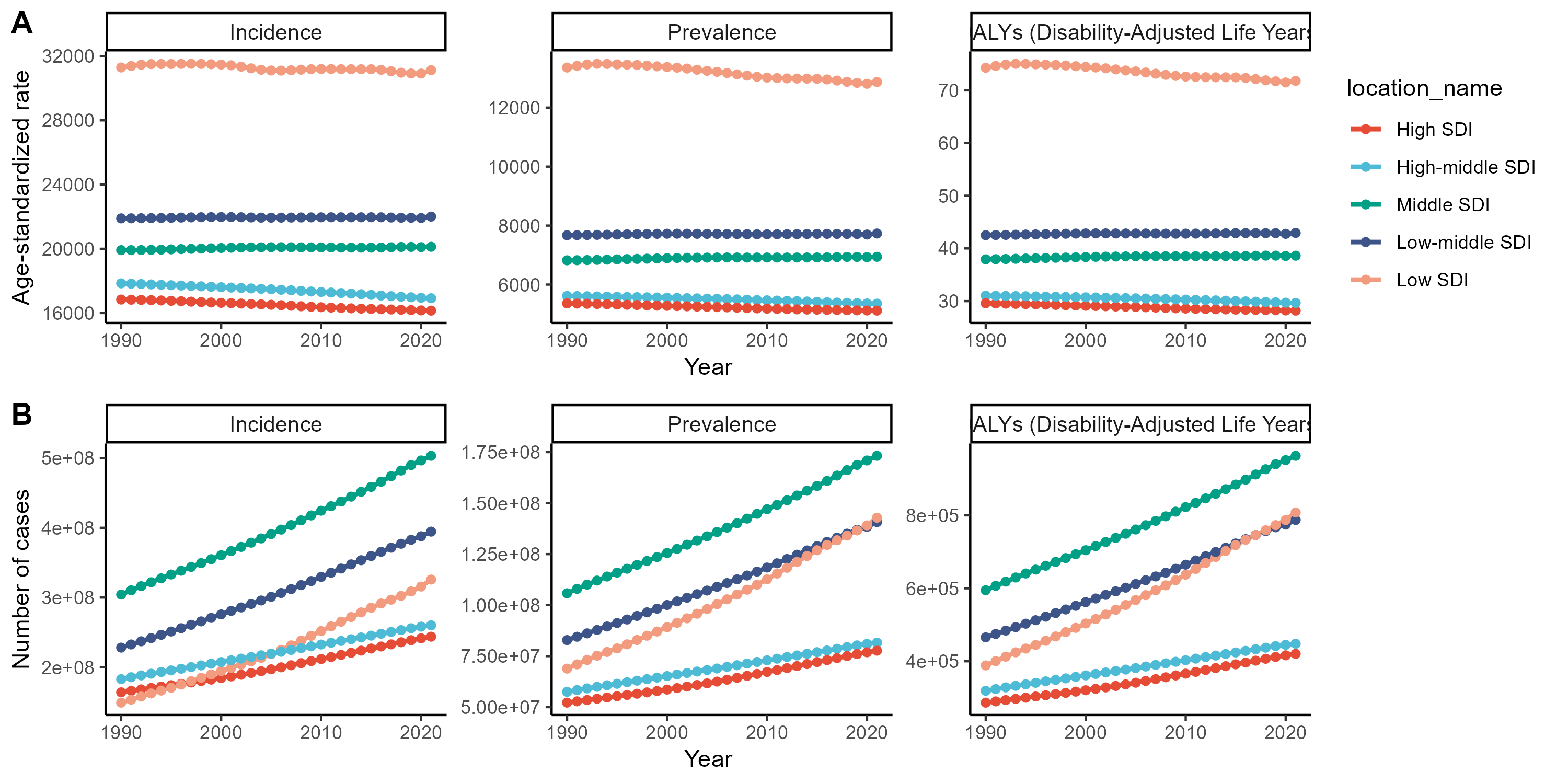

Supplement: Supplementary file 1 [file Data_Sheet_1.ZIP › Supplementary information/SFig. 9 90-21SDI.png]
